# Revisiting genetic artifacts on DNA methylation microarrays exposes novel biological implications

**DOI:** 10.1186/s13059-021-02484-y

**Published:** 2021-09-21

**Authors:** Benjamin Planterose Jiménez, Manfred Kayser, Athina Vidaki

**Affiliations:** Erasmus MC, University Medical Center Rotterdam, Department of Genetic Identification, Rotterdam, the Netherlands

**Keywords:** DNA methylation microarrays, Genetic artifacts, Monozygotic twins, meQTL

## Abstract

**Background:**

Illumina DNA methylation microarrays enable epigenome-wide analysis vastly used for the discovery of novel DNA methylation variation in health and disease. However, the microarrays’ probe design cannot fully consider the vast human genetic diversity, leading to genetic artifacts. Distinguishing genuine from artifactual genetic influence is of particular relevance in the study of DNA methylation heritability and methylation quantitative trait loci. But despite its importance, current strategies to account for genetic artifacts are lagging due to a limited mechanistic understanding on how such artifacts operate.

**Results:**

To address this, we develop and benchmark UMtools, an R-package containing novel methods for the quantification and qualification of genetic artifacts based on fluorescence intensity signals. With our approach, we model and validate known SNPs/indels on a genetically controlled dataset of monozygotic twins, and we estimate minor allele frequency from DNA methylation data and empirically detect variants not included in dbSNP. Moreover, we identify examples where genetic artifacts interact with each other or with imprinting, X-inactivation, or tissue-specific regulation. Finally, we propose a novel strategy based on co-methylation that can discern between genetic artifacts and genuine genomic influence.

**Conclusions:**

We provide an atlas to navigate through the huge diversity of genetic artifacts encountered on DNA methylation microarrays. Overall, our study sets the ground for a paradigm shift in the study of the genetic component of epigenetic variation in DNA methylation microarrays.

**Supplementary Information:**

The online version contains supplementary material available at 10.1186/s13059-021-02484-y.

## Background

DNA methylation is the most studied epigenetic biomarker. Particularly, 5-methylcytosine (5m-C) embedded within CpG sites in mammalian genomes has stricken epigeneticists for its abundance and core involvement in biological processes such as X-inactivation, imprinting, aging, and disease. From the wide range of methods that exist to detect CpG methylation, those relying on bisulfite conversion are particularly popular [1]: under basic conditions, unmethylated cytosines within single-stranded DNA molecules react with bisulfite and are deaminated to uracils; in contrast, 5m-C deamination is two orders of magnitude slower [2]. Bisulfite translates methylation information into sequence changes, for which standard genomic analytical methods can be deployed. In combination with next-generation sequencing, it yields whole-genome bisulfite sequencing (WGBS). Albeit nowadays considered the gold standard in methylomics, WGBS propagation has been hindered due to its high time and budget costs. Consequently, DNA methylation microarrays have gain popularity since they provide a more affordable alternative and hence are better suited for applications that require a large number of samples, such as epigenome-wide association studies (EWAS). Four generations of products have established Illumina’s hybridization-based microarrays as the leading platforms in human methylomics. We here focus on the previous Illumina Infinium HumanMethylation450 (450K) and current Illumina HumanMethylationEPIC (850 K), which cover over 450,000 and 850,000 CpG sites, respectively [3, 4].

**Fig. 1:**
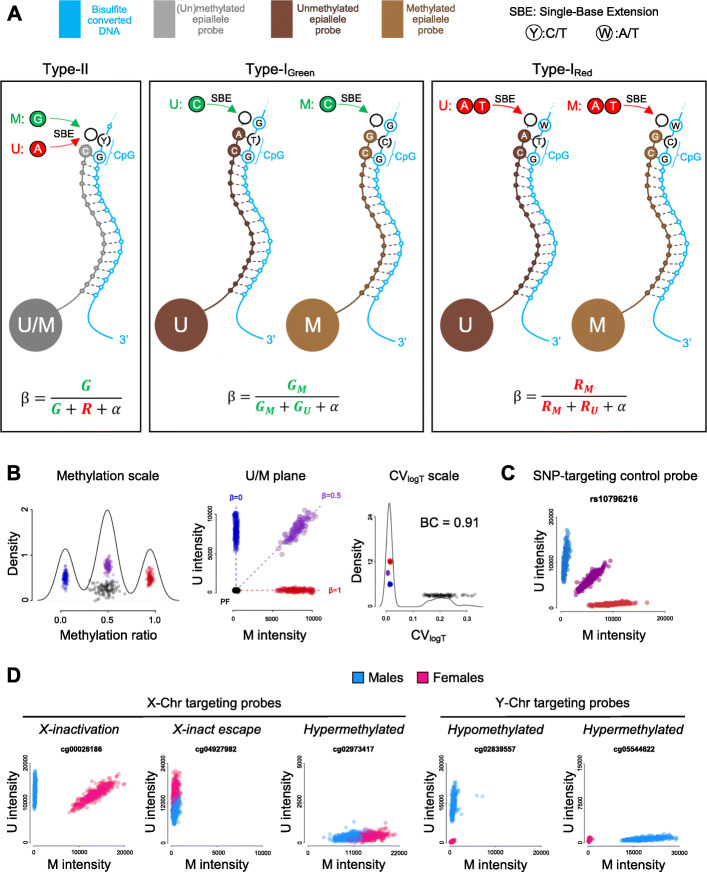
Overview of Illumina DNA methylation microarray probe design and general principles of UMtools. **A** Probe design and details on how DNA methylation ratios are quantified per Infinium probe type (type I^R^, type I^G^, or type II). **B** Correspondence between DNA methylation ratio (U/(U + M)), U/M plots, and CV_logT_ (≈ σ_log(U + M)_/μ_log(U + M)_) scales. DNA methylation ratio distribution is plotted as a kernel density estimation **C** U/M plot for a representative example of a 450K control probe targeting a high MAF bi-allelic SNP. Three clusters are formed corresponding to heterozygotes and homozygotes for each allele. **D** U/M plot for representative examples of sex chromosome-targeting probes. Males and females are highlighted in blue and pink, respectively

On these DNA methylation microarrays, hundreds of thousands of 50-nucleotide-long probes cover 3 μm silica beads that randomly self-assemble on a microarray’s substrate interspaced by 5.7 μm. The experimental protocol can be broken down to bisulfite conversion of the target genomic DNA, whole-genome amplification, enzymatic fragmentation, hybridization to the microarray, washing, staining, bead decoding, and fluorescence scanning. Detection is based on a single-base extension (SBE) step with labelled dideoxy-nucleotides triphosphate (ddNTPs): ddATP and ddTTP labelled with dinitrophenol (DNP) while ddCTP and ddGTP labelled with biotin, followed by an incubation with Cy5-labelled anti-DNP and Cy3-labelled streptavidin [5]. Fluorescence acquisition occurs in two separate channels corresponding to fluorophores Cy5 (Red, A/T) and Cy3 (Green, C/G). Concerning detection, three classes of probes simultaneously coexist on Illumina microarrays (Fig. 1A). Infinium type II (T-II) target both epialleles with a single oligonucleotide probe; the probe outstretches its 3′-end until one nucleotide before the targeted cytosine. As a result, SBE occurs at the target cytosine position and is informative in both fluorescence channels: green and red channels correspond to methylated (M) and unmethylated (U) epialleles, respectively. Besides, Infinium type I green (T-I^G^) and Infinium type I red (T-I^R^) target each epiallele with two different oligonucleotides probes. The 3′-end of T-I^G^ and T-I^R^ probes reaches the targeted cytosine and as a result, SBE occurs one nucleotide after the targeted cytosine. In this case, SBE for T-I^G^ or T-I^R^ is informative either on the green or the red channel, respectively. It is also important to note that Illumina probes may target a cytosine either at the plus or minus strand depending on the CpG site under consideration.

As with any probe-based approach, the inexorable abundance of genetic diversity in human populations, such as single-nucleotide variants (SNPs) or insertions and deletions (indels), poses a huge challenge in the design and in the application of DNA methylation microarrays. To face the potential impact of genetic artifacts in DNA methylation microarrays, early studies compiled probe exclusion lists by cross-referencing genomic coordinates targeted by the microarray probes and those of nearby genetic variants [6–8]. Nonetheless, these lists were crafted with limited mechanistic understanding of the DNA methylation assay and close to no empirical validation. Also, generic probe exclusion lists do not take into account population- or dataset-specific differences in allele frequencies [9]. Finally, genetic databases are constantly evolving and have limitations of their own, such as blind spots towards large indels like copy-number variations (CNV) or structural variants (SV), arising from the limitations of variant calling with short reads [10]. As result, probe exclusion lists are deemed to contain false positives and false negatives. Despite these limitations, there are currently no alternatives for dealing with genetic artifacts in the data preprocessing of DNA methylation microarrays that can ensure artifact-free data for the subsequent outcomes.

Discerning meaningful DNA methylation measurements from genetic artifacts can become a real challenge when additionally considering the strong influence that genetic variation can exert over the epigenome. This distinction is crucial in studies dedicated to the estimation of the heritability of DNA methylation variation [11, 12] or the discovery of methylation quantitative trait loci (meQTL) [13, 14]. In addition, DNA methylation microarrays are popular in cancer research—for example, employed for The Cancer Genome Atlas (TCGA)—even though tumoral genetic alterations have been found to alter the performance of the DNA methylation microarrays [15]. More recently, the 450K microarray has been repurposed in comparative genomic studies in apes [16, 17]; for this application, other alternative microarray platforms exist such as the novel Illumina HorvathMammalMethylChip40, able to target a wide range of mammalian species. However, since the same microarray technology is employed, it is equally susceptible to genetic artifacts [18]. Also, rare epigenetic variation may be confused for rare genetic artifacts [19]. Additionally, CpGs artifactually affected by underlying frequent genetic variants may display high inter-individual variation, and hence interfere in the search for variably methylated CpGs [20, 21]. Finally, genetic artifacts can provide counterfeit correlation between tissues; thus, they may interfere in the discovery of saliva/blood-brain proxy CpGs in epigenetic psychiatry [22, 23], between-tissue correlated CpGs [24, 25] and metastable epialleles [26]. In summary, understanding how genetic variants influence a popular DNA methylation assay affects a wide range of research fields and applications.

Last but not least, prior attempts to study genetic artifacts direct their analysis on the resulting DNA methylation ratio (e.g., beta-value). However, such analysis can well mask the effects of genetic artifacts; for example, probe failure is indistinguishable from intermediate methylation in the methylation ratio scale [15]. Also, prior attempts to understand and confirm the identity of genetic artifacts relied on scarce datasets including matched DNA methylation and genetic variant data [27]; this strategy can result in a large number of uncontrolled genetic variants due to variant calling and imputation limitations, largely depending on the chosen genotyping platform.

In this study, we aimed to contribute towards the increase in quality of DNA methylation data interpretation by proposing a novel strategy to assess genetic artifacts in methylomics. Our main objectives were (1) to develop and benchmark tools towards the quantification and qualification of genetic artifacts from fluorescence intensity signals, (2) to annotate the probes affected by genetic artifacts using genetic databases, (3) to deploy these tools on DNA methylation data on monozygotic (MZ) twins, acting as genetic controls, (4) to build a working understanding on the interference of genetic artifacts on the DNA methylation assay, (5) to challenge current practices that over-rely on probe exclusion lists, and (6) to develop a novel data-driven strategy that can discern between genetic artifacts and genuine genomic influence.

## Results

### UMtools: moving from DNA methylation ratios to raw fluorescence intensities

We consider that a genetic artifact in the Infinium assay has occurred when the measured methylation status of a targeted genomic region is biased by underlying genetic variants on the employed DNA template. This is counterpoint to genuine genetic effects in which genetic variants actually influence the methylation status of a genomic locus. However, due to the many intricacies involved in the assay (Fig. 1A), and the lack of analytical tools to validate hypotheses, our current understanding on how genetic artifacts operate and how they can be distinguished from genuine genetic influence has remained vague. Towards shedding light on this particularly elusive topic, we created UMtools, an R-package containing several data-driven tools for the analysis of raw fluorescence signals of Illumina DNA microarray data. Firstly, we introduce U/M plots, where U (unmethylated signal) is plotted against M (methylated signal), which are very suitable for exploratory purposes as they provide a quick visualization of the behavior of Illumina microarray probes. The analysis of DNA methylation microarray data on the original fluorescence U/M plane cannot only be as intuitive as in the DNA methylation ratio scale but can offer additional advantages in the study of genetic artifacts. Large DNA methylation microarray datasets suffer from between-array variation in the total fluorescence intensity, most likely introduced during the steps of staining and washing. As a result, data points corresponding to fully methylated or unmethylated samples for a given CpG tend to arrange as vertical and horizontal lines in the U/M plane, respectively (Fig. 1B). Intermediately methylated data points on the other hand encompass blurring on both channels in a dependent way, forming diagonal lines (Fig. 1B), only obscured by background fluorescence (T-I and T-II probes), differences in probe properties (T-I probes), or differences in fluorophores properties (T-II probes). Diversely, probe failure, occurring when solely background fluorescence is acquired, is evidenced as clumping of points near the origin (Fig. 1B). Though such signals are considered to be noise, if used to compute a methylation ratio typically result in intermediate methylation, since fluorescence backgrounds tends to be on similar ranges for both channels.

Secondly, to assign samples to clusters in a U/M plot, we adopted a bivariate Gaussian mixture model (bGMM) strategy. If the cluster-genotype correspondence is known, or simply predicted by examining the probe design and the alleles of genetic variants giving rise to artifacts, minor allele frequencies (MAF) can additionally be estimated from cluster counts. We can include a genetic control by taking into account in the computation only genotypes in agreement between MZ twins.

Furthermore, to move from a targeted scale towards a more systematic evaluation of probes at an epigenome-wide scale, we developed additional tools. We first devised the coefficient of variation of the logarithm of the total signal (CV_logT_), a new parameter that estimates noise-to-signal ratio per CpG and per sample (Fig. 1B). Its computation is based on the standard deviation of the intensity channels across beads (SD_Green_ and SD_Red_) stored on every raw microarray file (e.g., IDAT), but to the best of our knowledge has never been previously employed or discussed in the literature. While examining CV_logT_ distributions across individuals, one can observe that bimodality arises when a probe fails in some samples but not others; for example, a probe fails on a homozygote for a genetic variant that deters SBE but not on heterozygotes or homozygous for the other allele. Hence, the ambivalence in probe failure at an epigenome-wide scale can be quantified with our third tool, the bimodality coefficient of CV_logT_, BC(CV_logT_) [28]. We can also provide a genetic control to the ambivalence in probe failure, by computing the Pearson correlation of CV_logT_ between monozygotic twins, cor_MZ_(CV_logT_). Finally, we developed the K-caller, a computational approach that automatically assigns the number of clusters encountered in a U/M plot from the aggregation of samples in the U/M plane, based on density-based spatial clustering of applications with noise (dbscan) algorithm [29]. Here, the K-caller was calibrated using an independent set of markers (more details on section “Methods” and Additional file 1: Fig S1). Having a general-purpose K-caller at hand, it is now possible to systematically detect genetic artifacts beyond probe failure.

To benchmark our developed tools, we chose the publicly available dataset from the E-risk twin cohort that includes 450K-based DNA methylation data derived from whole blood samples from 426 British MZ twins at age of 18 [11]. Using the E-risk dataset allows us to control for genetics via agreement between MZ twin pairs, while minimizing aging-related methylation variation since study participants are equally aged. In addition, for our benchmarking, we targeted control SNP and sex chromosome-targeting probes, as their behavior has been well documented at the DNA methylation scale [30] (Fig. 1C, D). Extending this knowledge to the U/M plane, control SNP probes targeting high MAF bi-allelic SNPs form three clusters corresponding to homozygotes (AA, BB) and heterozygotes (AB). Secondly, probes targeting the Y-chromosome (Y-probes) tend to form exclamation mark-like shapes as they fail on females, while detecting either fully methylated or unmethylated in males (Fig. 1D). Thirdly, sex differences on probes targeting the X-chromosome (X-probes) are often promoted via X-inactivation: to compensate for the doubling dosage of genes in the X-chromosomes in females, one of the copies is randomly inactivated via large-scale targeted methylation. As a result, X-probes are often intermediately methylated in females (X^M^X^U^) and either 0 or 100% methylated in males (X^U^ or X^M^); hence, separating males and females in two distinct V-shape clusters in the U/M plane (Fig. 1D). In contraposition, some regions are fully hypo- or hypermethylated in both females and males (X^U^X^U^/X^U^ or X^M^X^M^/X^M^). However, despite the X-chromosome copy-number difference, such regions do not present full separation between males and females in the large E-risk cohort because of the spread caused by batch effects (Fig. 1D). Full separation though can be observed in smaller datasets, which are less affected by batch effects (Additional file 1: Fig S2). After excluding some known problematic probes [6, 7], X-probes were segmented into X-inactivation, escapees, and hypermethylated categories with the help of the previously published classification [30]. Small- and large-scale tools performed greatly on sex chromosome and SNP-targeting probes, here summarized as a set of scores (Table 1).

**Table 1:** Benchmarking of UMtools on sex chromosome- and SNP-targeting probes

*UMtools*					
**Tool**	**Scale**	**Purpose**			
U/M plot	Targeted	Cluster visualization			
bGMM	Targeted	Cluster assignment for a target number of clusters			
BC(CV)	Epigenome-wide	Ambivalence in noise-to-signal ratio detection			
cor_MZ_(CV)	Epigenome-wide	Genetic control for noise-to-signal ratio			
K-caller	Epigenome-wide	Cluster counting			
*Benchmarking*					
**Markers**		**ChrY**	**ChrX**_**inact**_	**ChrX**_**hypermeth + escape**_	**SNP probes**
**# probes**		266	3,981	3,028	65
**Expected K**		2	2	1 (large n)	3
**Probe failure in females**		Yes	No	No	No
*bGMM* *(K = 2 or 3)*	*Twin cluster assignment agreement*	0.994	0.991	0.479^a^	0.997
*BC(CV*_*logT*_*) and cor*_*MZ*_*(CV)*	*Genetics-related probe failure*	0.951	0.001	0.001	0.000
*K-calling*	*Correct # clusters predicted*	0.977	0.902	0.999	1.000

^a^ Full separation between males and females is not observed in a large cohort as E-risk (Fig. 1D); it can be seen though in smaller datasets (Additional file 1: Fig S2A) that are not so strongly affected by batch effects

We also aimed to compare the performance of our newly developed tools with previously published tools designed for DNA methylation microarray data that employ the methylation ratio scale. On the one hand, we compared BC(CV) with the detection *p* value of negative control probes (pNC), the *p* value with out-of-band array hybridization (pOOBAH) [15], and the *p* value with non-specific fluorescence (pNSF) [31]; all of which are used to evaluate successful probe performance. While detection *p* values allow to get a black-or-white picture, BC(CV) can reflect quantitively noise fluctuations in fluorescence signals (Additional file 1: Fig S3). On the other hand, we also compared K-caller with the existing published tools. We first identified the MethylToSNP tool [32], which uses tri-modality in beta-values as evidence for confounding by polymorphisms. However, we discarded this approach: not only does it not discern from genuine methylation influence that often gives rise to tri-modality, but it also ignores the vast majority of genetic artifacts which generate bimodal distributions. In addition, Gaphunter relies on gaps in DNA methylation profiles as a signature for genetic variant confounding [27]. When testing Gaphunter using default parameters, it correctly predicted the number of clusters for 16.9 % of ChrY probes and 55.9 % of ChrX probes subject to X-inactivation, a substantially worse performance in comparison to the K-caller (Additional file 1: Fig S4). Finally, we aimed to also test the univariate Gaussian mixture model clustering [33], but its source code was unavailable.

### Annotating genetic variants for 450K probes using dbSNP151

Having a set of newly developed benchmarked tools ready, we firstly annotated SNPs and indels associated to probes in the 450K and EPIC platforms based on dbSNP151 in six groups: SNP or indels at CpG sites, at SBE sites (for type I probes), and at other probe hybridizing positions (Additional file 1: Fig S5A). As an overview, we ran the epigenome-wide tools on all CpGs associated to genetic variants in the E-risk cohort based on 450K data. Difference in distributions of BC(CV_logT_), cor_MZ_(CV_logT_), and number of clusters are evident at this stage, concordant with the appearance of genetic artifacts (Additional file 1: Fig S5B-C). From this point onwards, we will dive deeper into the different subcategories.

### SNPs at CpG/SBE sites offer a wide manifestation of genetic artifacts

**Fig. 2:**
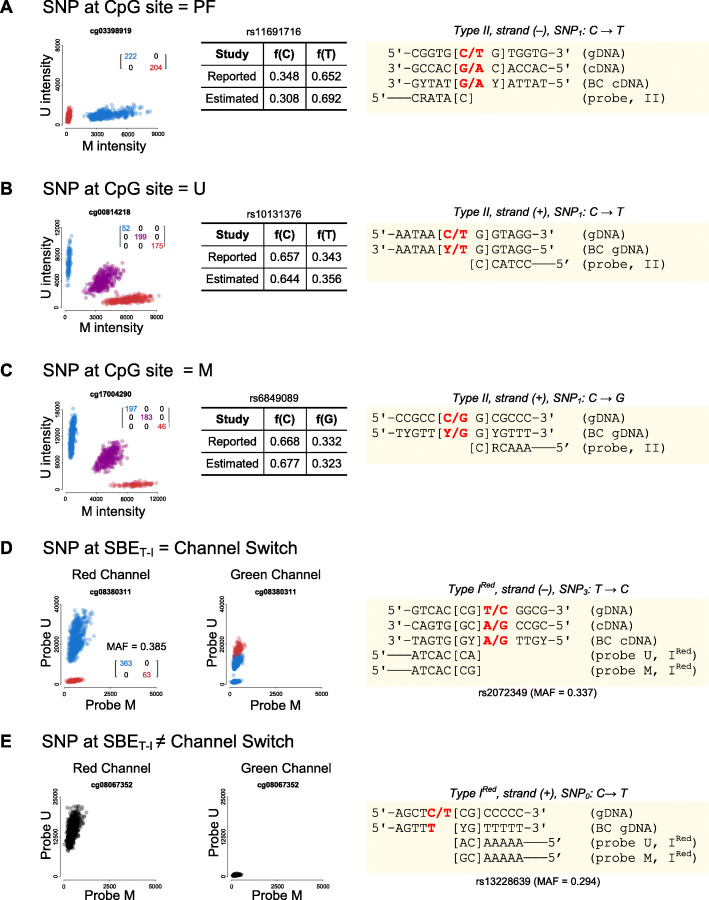
Manifestation of how SNPs at CpG/SBE sites can affect the Infinium assay. U/M plots, MAF estimation, and probe design of a SNPs disguising as **A** U (SNP = U) or **B** M epiallele (SNP = M), **C** of a SNP causing probe failure (SNP = PF) and **D** of a SNP at an SBE site of a type I probe causing detection channel switch or **E** not. Cluster counts were estimated from U/M plots via bivariate Gaussian mixture models; only concordant monozygotic twin pairs were taken into account in the computation of MAF (genetic control); MZ twin-to-twin agreement matrices are also available on the plots employing the same color coding as the clusters in the U/M plot. Probe designs are highlighted in yellow boxes and SNPs are denoted in bold red. Reported MAFs correspond to 1000 genomes (phase 3) of EUR ancestry (*n* = 1006)

SNPs are the most frequent source of genetic artifacts on the 450K microarray fluorescence intensity signals (Additional file 1: Fig S5A). Particularly, SNPs at CpG/SBE sites are highly predictable and manifest themselves in a plethora of ways depending on the probe type (T-I^Red^, T-I^Green^, T-II), targeted strand (plus or minus), SNP position, and alleles [27]. Unlike T-II probes, for which SBE is performed on the targeted cytosine, T-I probes prime SBE on the position following the targeted cytosine. As a result, T-I probes are strongly susceptible to SNPs at three positions (CpG site and SBE positions) while T-II probes are only at two (CpG site positions only). Based on their expected manifestation, we subclassified 450K probes targeting CpG/SBE sites with known SNPs (dbSNP151) into 16 different categories (Table 2). Our classification is in close concordance to prior predictions [27], but greatly simplified. In summary, SNPs under a wide range of categories can cause probe failure when homozygous (Fig. 2A). In addition, a SNP can disguise as the U or M epiallele (Fig. 2B, C). In this case, whether the SNP manifests as a genetic artifact or not depends on the DNA methylation context of the genomic region: a CpG-SNP disguising as the U epiallele will cause a genetic artifact if it lies within a methylated region, and vice versa. Particularly for T-I probes, SNPs at SBE sites can also reverse the detection fluorescence channel or simply neutral towards the methylation estimation itself (Fig. 2D, E). Although T-I probes subject to no channel change display genuine detection, they are still included in EWAS probe exclusion lists, though some authors have offered strategies to rescue them [34].

**Table 2:**
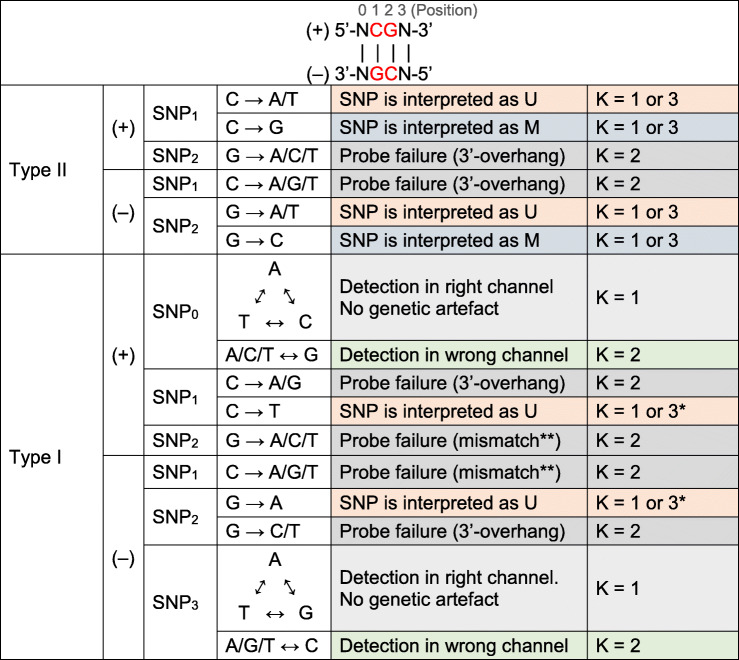
Sixteen categories of CpG/SBE-SNPs. Reference allele is assumed to be the targeted allele in Illumina’s probe annotation, which does not necessarily correspond to the major allele

^a^ If # internal CpGs ≫ 1 and locus is methylated, sometimes *K* = 2. ^b ^Mismatch at position prior to 3′-end of the probe

**Fig. 3:**
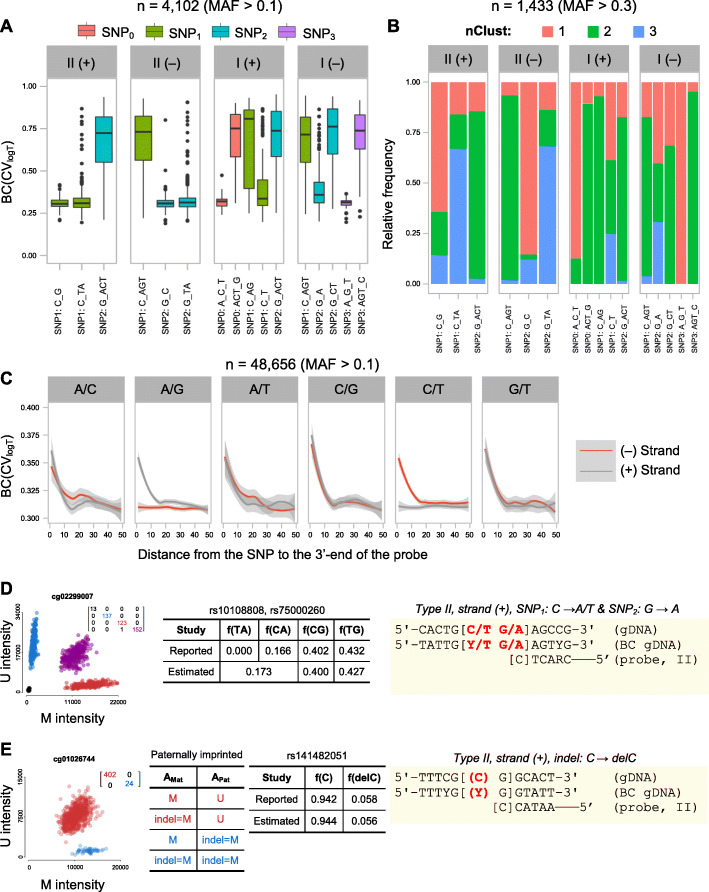
Systematic evaluation of CpG/SBE–SNPs and probe-SNPs and examples of CpGs subject to genetic artifacts with additional levels of complexity. **A** Ambivalence in noise-to-signal ratio, BC(CV_logT_), distribution across CpG/SBE-SNP categories (SNPs with MAF < 0.1 were excluded from this analysis). **B** Relative distributions of U/M plane cluster counts estimated by the K-caller across CpG/SBE-SNP categories (SNPs with a MAF < 0.3 were excluded from this analysis). **C** BC(CV_logT_) of bi-allelic probe-SNPs as a function of distance to the 3′-end of the probe and targeted strand (SNPs with a MAF < 0.1 were excluded from this analysis). The obtained curves have been smoothed via local regression (loess). **D** Genetic artifacts on a CpG site associated with two contiguous SNPs and the estimation of haplotype frequencies from cluster counts in the U/M plot via general-purpose optimization. Reported haplotype frequencies were obtained from LDhap. **E** Genetic artifact caused by an indel that additionally interacts with imprinting. A_mat_ and A_pat_ indicate maternal and paternal alleles, respectively. Reported MAFs correspond to 1000 genomes (phase 3) of EUR ancestry (*n* = 1006). Allelic frequencies were estimated from cluster counts estimated via bivariate Gaussian mixture models from U/M plots and only concordant monozygotic twin pairs were taken into account in the computation. Twin-to-twin agreement matrices are also available on the plots employing the same color coding as the clusters in the U/M plot. Probe designs are highlighted in yellow boxes and SNPs/indels are denoted in bold red

Having manually confirmed the manifestation of genetic artifacts in a handful of examples via SNP calling, MZ twin agreement, and MAF estimation in close consensus with the 1000 Genomes Project (phase 3) for European ancestry, we extended our analysis to the whole set of CpG/SBE-SNPs by using our newly developed epigenome-wide tools. Our expectations for BC(CV_logT_), cor_MZ_(CV_logT_) and K-calling closely matched our observations with the exception of type I (+) SNP1: C↔T and type I (−) SNP2: G↔A (Fig. 3A-B, Additional file 1: Fig S6-7). SNPs at the CpG/SBE sites of these probes were expected to disguise as the unmethylated epiallele; hence, form one or three clusters in the U/M plane, depending on whether the region was unmethylated or methylated, respectively. Instead, we observed that these probes were enriched for genetic artifacts forming two clusters and associated to probe failure, at a frequency that was too high to be explained by simply misclassifications of the K-caller. Interestingly, we also noticed that the failing type I probes were typically targeting methylated regions and contained a higher number of internal CpGs compared to non-failing probes (linear model, interaction, *p* value = 3.73 × 10^−8^, Additional file 1: Fig S8). Using this information, we propose the following model to explain the discrepancy: when the SNP is disguised as the unmethylated epiallele, neighboring CpGs also targeted by the type I probe remain methylated. As a result, neither type I probes targeting the fully methylated or fully unmethylated haplotypes can bind to initiate SBE at the target locus, hence resulting in probe failure (Additional file 1: Fig S8D).

### SNPs on the remaining probe binding sites can cause probe failure

Unlike genetic variants at CpG/SBE sites, SNPs at the sites beyond the CpG/SBE site may only manifest themselves as genetic artifacts via probe failure. As expected, the closer a genetic variant is to the 3′-end of the probe, the more likely it is to cause probe failure. However, Illumina microarrays are based on rather long 50-nt-long probe; hence, the interference of SNPs is quickly diluted the further it is located from the 3′-end of the probe [34]. With our epigenome-wide tools at hand, we tested BC(CV_logT_), cor_MZ_(CV_logT_) and K-calling dependencies on the distance to the 3′-end of the probe, strand, and SNP alleles (Fig. 3C, Additional file 1: Fig S9-10). In summary, SNP effects cannot be detected any longer after 15 bp from the 3′-end. More notably, we noticed that C/T and G/A SNPs did not cause probe failure at CpGs targeted in the plus and minus strand respectively, independently of its position from the 3′-end. Though it has not been reported before, it can be easily explained: bisulfite conversion makes the SNP indistinguishable from its fully converted DNA, except in the context of a methylated CpG site which remains non-converted. Outstandingly, probes affected by such SNPs are also excluded by EWAS studies, as this criterion was not considered when compiling existing probe exclusion lists.

### Indels can result in a wide range of genetic artifacts

During our annotation, we also discovered indels associated to CpG/SBE/probes sites that are also expected to alter the 450K fluorescence intensity signals in an artifactual manner. Unlike SNPs, however, not all probe exclusion lists used in EWAS contain probes potentially affected by indels. Although additional complications are entailed by variable lengths and positions with respect to the CpG site, CpG/SBE indels can manifest in the same ways as CpG/SBE-SNPs. Typically, indels remove the whole CpG site and cause probe failure when being in the homozygous state (Additional file 1: Fig S11A), although we also identified insertions disguised as the U/M epialleles (Additional file 1: Fig S11B-C), or insertions that maintain or reverse the detection fluorescence channel in type I probes (Additional file 1: Fig S11D-E). Finally, given that our epigenome-wide tools allow to detect probe failure without requiring genetic annotation, we explored putative unregistered DNA variants in our data. Stunningly, we found an example of a large unannotated indel affecting a total of six 450K probes (Additional file 1: Fig S12), which is possibly not registered in dbSNP yet due to the limitations of short-read sequencing technologies to variant-call large genomic re-arrangements.

### Higher-order genetic variants and joint interaction with genuine biological variation

In the process of analyzing the 450K set of probes, we also identified examples subject to additional levels of complexity. Firstly, we report for the first time the effect of triallelic SNPs on Illumina DNA methylation microarrays; additional consideration must be taken when dealing with them as, under the Infinium detection assay, two of the alleles are simply indistinguishable (Additional file 1: Fig S13). In addition, we found several instances of SNPs located at both C and G within CpG sites; these cases manifest as SNPs confused for the U/M epiallele over-imposed with probe failure, in total forming four clusters (Fig. 3D). To the best of our knowledge, these have never been reported before, probably because probe failure and intermediate DNA methylation are indistinguishable at the methylation scale. We hypothesize that haplotypic frequencies could be accurately estimated from the counts of samples at each cluster called by a bGMM. We employed general-purpose optimization to find parameters that minimize our theoretical expectations. This way, we obtained haplotypic frequency estimates in high agreement with those reported at LDhap for European ancestry (Fig. 3D) [35].

**Fig. 4:**
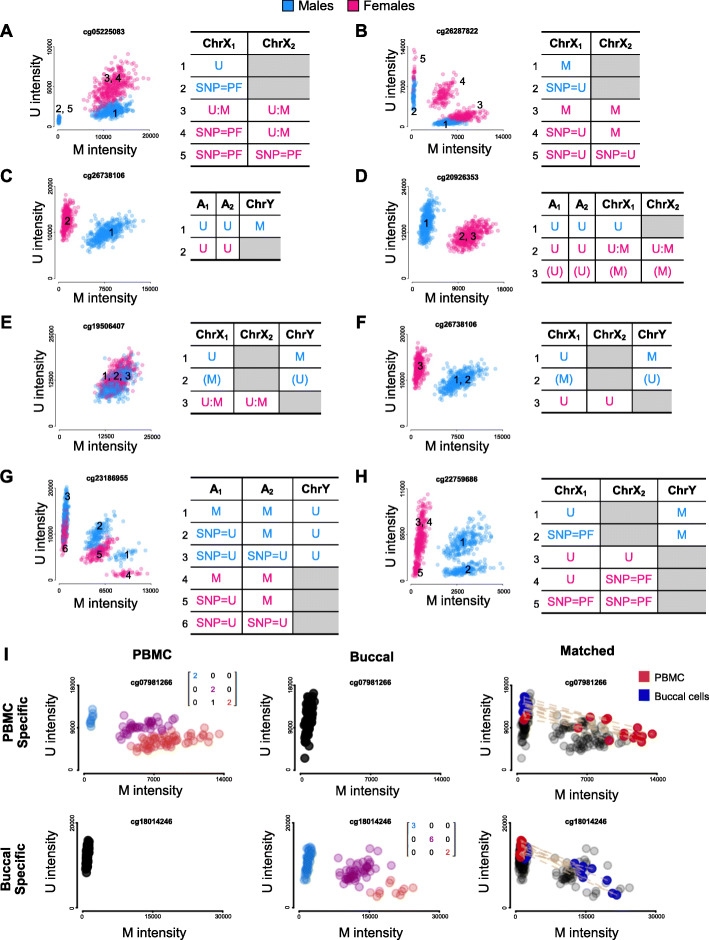
Genetic artifacts that interact with sex and probe cross-reactivity (cross-hybridizing at loci not initially targeted by the design). **A** Allosomal probe associated to the SNP rs56157110 causing probe failure (SNP=PF), additionally influenced by X-inactivation; U:M denotes that one epiallele is methylated randomly per cell. Males and females are highlighted in blue and pink, respectively. **B** Allosomal probe associated to the SNP rs7886395 that disguises as U (SNP=U), additionally interacting with X-hypermethylation. **C** Autosomal probe, cross-reactive (CR) towards ChrY. **D** Autosomal probe, CR towards ChrX; parentheses denote the less likely scenario. **E** Allosomal probe CR to both chrX/Y, additionally influenced by X-inactivation. **F** Allosomal probe, CR to ChrX/Y, additionally influenced by X-inactivation escape. **G** Autosomal probe associated to the SNP rs842416 that disguises U, additionally CR towards ChrY. **H** Allosomal probe, CR to ChrX/Y, associated to an undetermined variant that causes probe failure at ChrX, additionally influenced by X-inactivation escape. **I** Peripheral blood mononuclear cells (PBMC) and buccal cells tissue-specific genetic artifacts produced by SNPs rs28780111 and rs12720020, and on first and second row, respectively. First and second column correspond to methylation in PBMC and Buccal cells. Confusion matrices represent cluster correspondence between technical replicates within each tissue. On the third column, PBMC and buccal patterns are overlayed with arrows that connect matched PBMC-Buccal samples available on both tissues

Genetic artifacts are particularly concealed when interacting with non-artifactual biological variation. For example, we identified some examples of genetic artifacts interacting with imprinting and X-inactivation (Fig. 3E, Fig. 4A-B). Although straying from genetic artifacts per se, given the highly intuitive results obtained with our tools, we also considered extending our approach to other sets of troublesome probes in the 450K microarray. Particularly, cross-reactive (CR) or non-specific probes are promiscuous probes predicted to hybridize at several loci in the human bisulfite-converted genome. CR probes are hard to avoid in the design of the DNA methylation microarrays, not only given the high content in repetitive sequences of the human genome, but also because of the reduced sequence complexity resulting from bisulfite conversion. As expected, diagnosing cross-reactivity is subject to the same issues as predicting genetic artifacts in silico and some recent work sheds light into this [36]. We focused on autosomal probes cross-reactive towards chromosome X or Y, as well as allosomal probes targeting both sex chromosomes and we observed and explained a huge diversity in U/M plots (Fig. 4C–H).

Lastly, we hypothesize the existence of tissue-specific genetic artifacts: if a SNP confused as U happens to be in a methylated region in tissue-A, but is unmethylated in tissue-B, it will only cause a genetic artifact in tissue-A, but no in B (and vice versa). To test this idea, we employed existing matched data from both peripheral blood mononuclear cells (PBMC) and buccal epithelial cells (BEC) [24]. Conveniently, the employed dataset also includes technical replicates in both tissues, which we used as genetic controls. As a result, we successfully identified several examples of this hitherto unreported phenomenon (Fig. 4I).

### False positive and negative genetic artifacts in probe exclusion lists

We also aimed to examine the limitations of probe exclusion lists. However, this would require the insurmountable task of examining the intrusion of genetic artifacts and extrusion of healthy probes in the entire published epigenomic literature until now. Aiming to be as conservative as possible, we instead examined the study from van Dongen et al., a significant milestone in understanding the heritability of DNA methylation [12]. Though most studies deploy out-of-the-shelf probe exclusion lists assuming absence of population differences, the authors of this study alternatively implemented an improved population-specific probe exclusion scheme based on the Dutch population MAFs from the GoNL Project [37]. While we could confirm that the set of excluded probes was highly enriched in probes associated to genetic artifacts, we could still detect minor portions of genetic artifacts leaking into the heritability ranking of van Dongen et al. (Additional file 1: Fig S14-15). Despite only a few, these probes tend to be highly enriched at the top of the heritability ranking (Additional file 1: Fig S15B). For example, CpGs highlighted in Fig. 3E and Additional file 1: Fig S11A-C have been wrongly ranked with very high heritability estimates (0.98, 0.78, 0.98, and 0.97, respectively). Although the minor leaking of genetic artifacts does not challenge their overall conclusions, researchers aiming to follow-up their heritability outcomes would start from the top of the ranking and, hence, face a low validation rate. Counterpoint to this problem, while intending to exclude as many potentially artifactual probes as possible, a large number of false positives have also been removed, disabling the chance for new biological discoveries (Additional file 1: Fig S15E). With this lower bound in mind, we expect that numerous studies whose data analysis was executed with a more rudimentary approach may end up with a larger leakage of genetic artifacts.

### Discerning real genetic influence from genetic artifacts—the example of NINJ2-intron meQTL

**Fig. 5:**
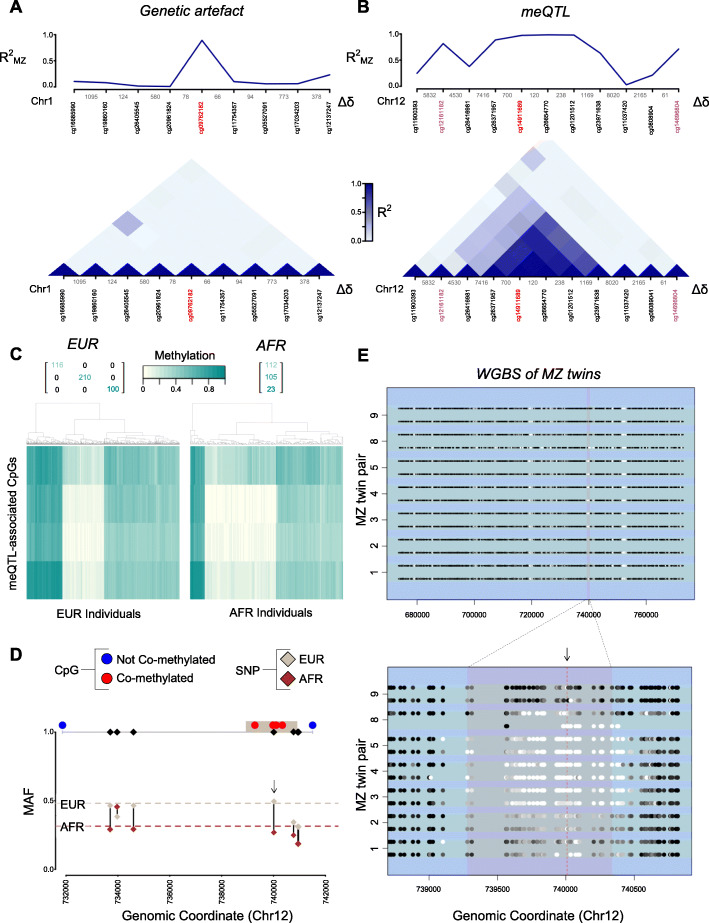
Distinguishing between genetic artifacts and genuine genetic influence on DNA methylation by co-methylation: an extensive follow-up on the meQTL of the first intron of the NINJ2 gene. **A** Squared correlation between the DNA methylation ratio of monozygotic co-twins and squared co-methylation across samples as a function of the genomic coordinate of a representative genetic artifact, and **B** the NINJ2 meQTL. *M*-values were used instead of methylation ratios in the computation of correlations. Plots are centered around the CpG highlighted in red. CpGs highlighted in purple correspond to those assessed to be potentially artifactual probes. Distances between CpGs targeted by contiguous probes (Δδ) are also highlighted in the co-methylation plot. **C** Methylation ratio heatmap of the meQTL at the first intron of NINJ2 in datasets of EUR and AFR ancestry. Counts for each of the three clusters are included, obtained from the shown dendrogram. For EUR ancestry, twin-to-twin agreement is represented as a matrix. For AFR, this is not shown as the data consists of unrelated individuals. **D** Employing frequencies in EUR and AFR estimated from U/M plot to map NINJ2 meQTL’s most likely *cis-*causal SNP (highlighted with an arrow). Only SNPs in the region with MAF > 0.2 are shown. CpGs and SNPs are represented as circles and rhombi, respectively. Red and blue CpGs correspond to those within or outside the expected co-methylation window (average genomic position across co-methylation CpGs ± 1 kb, colored as a brown rectangle). **E** Confirmation of meQTL co-methylation across the NINJ2 first intron region, inter-individual variation, and co-twin similarity in DNA methylation using whole-genome bisulfite sequencing data of MZ twins in whole blood. Twin pair numbering was kept as in the original dataset (E-MTAB-3549)

Detecting genetic variants that cause probe failure is possible with both detection *p* values (standard practice) and our newly presented BC(CV) approach. However, DNA variants that camouflage as the U or M epiallele display seemingly healthy fluorescence intensities that without information about underlying SNPs cannot be differentiated from a strong meQTL. To discriminate an meQTL from a genetic artifact, we propose the use of co-methylation, namely the tendency of nearby CpGs to pose similar DNA methylation levels in distance ranges of up to 1 kb [38]. More specifically, while a genetic artifact that manifests as the U/M epiallele causes high DNA methylation variation, this is not expected to be correlated with the surrounding genuine CpG sites (Fig. 5A). As a result, the presence of co-methylation with nearby CpGs can be employed as evidence for true biological variation.

To demonstrate this, we focus our validation on an example localized on chromosome 12 at the first intron of the ninjurin-2 (*NINJ2*) gene that had been previously included in lists of discovered meQTL [13], but so far lacked any follow-up (Fig. 5B). That being said, ultimate confirmation of an meQTL requires functional studies, in which in vivo genome editing is causally linked to DNA methylation changes in the region. Nevertheless, plenty of additional evidence can be gathered towards pinpointing a putative causal variant via an in silico approach. Both co-methylation and tri-modality were observed in populations of European (EUR) and African (AFR) ancestry, congruent with a *cis*-acting co-dominant genetic variant controlling the methylation status of the region (Fig. 5B, C, Additional file 1: Fig S16A). We estimated MAF across co-methylated sites of 0.48 and 0.31 for EUR and AFR ancestry, respectively (minor allele corresponding to the methylated epiallele). To additionally pinpoint potential *cis*-causal variants, we took advantage of the observed population-specific MAFs: only one variant within the co-methylation window, the C>G SNP rs34038797, displayed agreement between measured and reported MAF for both populations (Fig. 5D). The variant in question has been previously reported in GWAS to be strongly associated to Platelet/Lymphocyte/Monocyte count [39]. To confirm meQTL mapping observations and employing matched 450K and SNP array data enhanced with SNP imputation, we observed consistency between the DNA methylation status of the meQTL and the alleles of the putative causal variant (Additional file 1: Fig S17).

Additionally, given that meQTL mapping was performed on whole blood (a complex mixture of cell types), we wondered how the meQTL would behave in pure cell types. Indeed, isolated cell populations studied in whole blood and cord blood displayed the distinctive three-level methylation status, consistent across cell types of the same individual (Additional file 1: Fig S16B-E). Notably, this meQTL behavior was less clear in adipose tissue (Additional file 1: Fig S16F-G). This could be explainable by cellular infiltration being the source of the methylation pattern rather than local resident adipocytes or simply by methylation variation between tissues. Moreover, we aimed to assess whether the *NINJ2* meQTL appeared upon differentiation or was already present in early blood progenitors. We observed the same patterns of co-methylation in both early progenitors and differentiated cell types, setting the time of DNA methylation establishment prior to differentiation (Additional file 1: Fig S18). Finally, we confirmed our observations on the *NINJ2* meQTL by employing WGBS data on MZ/DZ twins and unrelated individuals in both whole blood and adipose tissue. Particularly, we verified that (a) all MZ twin pairs always shared equivalent methylation status in the meQTL in contrast to DZ twins, (b) inter-individual variation was apparent at the co-methylation window, but not outside, and (c) the meQTL was less striking in adipose tissue compared to whole blood (Fig. 5E, Additional file 1: Fig S19-20). On another note, building upon our evidence for this locus, we aimed to shed light on the putative mechanism of the *NINJ2*-intron meQTL. We employed motifbreakR [40] to predict the disruptiveness of the SNP on a potential transcription factor binding site (TFBS) against the DNA motif databases HOMOCO, HOMER, ENCODE, and FactorBook, together with the SNP2TFBS webtools [41] (Additional file 1: Fig S21, S22A-B). We also interrogated a large amount of chromatin immunoprecipitation (ChIP)-seq data with the help of ChIPSummitDB [42] and Unibind [43] (Additional file 1: Fig S22C-D). Integrating all this information, we predicted that the putative causal variant could very likely act as a switch for an Erythroblast Transformation Specific (ETS)-TFBS, granting rs34038797 the status of a putative regulatory SNP (rSNP). Additionally, we discovered that the putative meQTL-causal SNP was also a histone acetylation quantitative trait locus (haQTL) [44], an expression quantitative trait locus (eQTL) [45], a chromatin accessibility quantitative trait locus (caQTL) [46], and a transcript usage quantitative trait locus (tuQTL) [47]. All this information is conveniently integrated at QTLbase webtool [48].

Based on these observations, we finally compiled a mechanistic model (Additional file 1: Fig S23). For rs34038797>C, the ETS-TFBS is operational allowing the recruitment of an activating ETS-family TF that mediates the epigenetic activation of the locus (hypomethylation, H3K27ac and increase in chromatin accessibility). This coincides with the active transcription of an antisense long non-coding RNA (lincRNA) *NINJ2-AS1*. For this allele, the main transcript variant expressed in blood is *NINJ2-205*. On the other hand, for rs34038797>G, the ETS-TFBS has been disrupted and as a result the epigenetic state of the locus is inactive (hypermethylation, absence of H3K27ac, low chromatin accessibility). This coincides with the silencing of *NINJ2-AS1* and an exon inclusion event that replaces *NINJ2-205* expression for the longer *NINJ2-202* transcript. Exon inclusion correlating with hypermethylation has been previously described before [49]. For heterozygotes, co-dominance results from the *cis*-acting nature of the biological mechanism.

## Discussion

The Illumina 450K and subsequent EPIC platforms have allowed epigenome-wide DNA methylation analysis, vastly used for the discovery of novel DNA methylation variation in health and disease. But despite its huge popularity in research studies and clinical applications, challenges remain towards accounting for potential genetic artifacts arising from the huge genetic diversity of human populations that interfere with a probe-based hybridization methylation quantification approach. So far, the current strategy is to simply exclude probes with high risk for genetic artifacts as judged from the proximity of genetic variants to the microarray’s probes, disregarding any mechanistic insight. In this study, almost a decade since 450K microarray’s commercial distribution, we have revisited this topic once again but this time, by directly examining fluorescence intensities and using MZ twins as genetic controls. Though our characterization was based on 450K data, since there is no technological upgrade on the EPIC platform but simply an increase in the number of targeted sites, we expect that our conclusions are valid on both arrays.

Illumina DNA methylation microarrays were inspired on the GoldenGate platform, a two-channel fluorescence microarray initially developed for SNP genotyping. Even though it is standard practice in SNP array analysis to perform variant calling employing both fluorescence channels on the bivariate plane, this has not been the case for calling genetic artifact in CpG methylation microarrays. Given the analogy between both problems, our novel approach seems like a natural extension of this strategy towards DNA methylation. Moving from the one-dimensional DNA methylation ratio to the bi-dimensional U/M plane has not only led to the differentiation of probe failure from intermediate DNA methylation but has also taken SNP- and K-calling from DNA methylation data to yet unseen precisions. In addition, the use of MZ twins as genetic controls helped us clear the need for matched genetic and epigenetic data, resulting in large sample sizes, and enabling applications such as MAF estimation. Such controls are valid on artifactual probes as fluorescence signals are determined by genetics. However, this is not applicable on CpG sites under true epigenetic variation like meQTLs since MZ twins can be additionally influenced by environmental variables. Additionally and for the first time, we have described the use of DNA methylation microarray data to estimate SNP and haplotype MAF, to understand the effect of unannotated large indels and triallelic CpG-SNPs, to predict how internal CpGs can cause probe failure in type I (+) SNP1: C↔T and type I (−) SNP2: G↔A or how SNP alleles are relevant for probe binding sites as well as to characterize the interaction between genetic artifacts with X-inactivation, imprinting and tissue-specific methylation. Lastly, we also developed a novel strategy to differentiate meQTLs from genetic artifacts based on co-methylation. In definite, we have provided an atlas to aid researchers navigate through the huge diversity of genetic artifacts encountered on Illumina methylation probe-based microarrays.

These analyses were only possible based on the novel low- and high-throughput data analysis tools we developed first as part of UMtools. Since a wide range of R-packages have already been developed to analyze data from Illumina’s DNA methylation microarray platforms (minfi [50], watermelon [51], RnBeads [52], ChAMP [53], to name a few), UMtools focuses on the analysis of raw fluorescence intensities and may serve as a supplement to the standard libraries in tasks associated to quality control, exploratory, and post hoc analysis (suggested guidelines are provided in Additional file 2). We highlight our significant efforts towards not only benchmarking our new methods by contrasting the obtained predictions to real outcomes, but also by comparing them with existing tools and by making them available. This is not always the case for similar purpose tools in which either the source code is unavailable [33], or the benchmarking was performed over a handful of true positive examples, with less effort towards quantifying false positives and negatives [27]. Particularly, in our novel approach we highlight the potential of SD_Green_ and SD_Red_ matrices that are stored on every IDAT file but, to the best of our knowledge, have never been used before in the literature. We believe that this is due to the scarcity of information concerning Illumina’s proprietary IDAT format and the current tendency of employing pre-normalized data for DNA methylation analysis. For further information, we recommend the documentation of the illuminaio R-package [54]. Also, we emphasize that there is no substitute for raw data, as certain information is lost during preprocessing, with the additional influence of preprocessing itself being considerable, but this has already been discussed elsewhere [55]. Future applications pursuing a better understanding of the microarray’s measurement error could greatly profit from considering SD_Green_ and SD_Red_; for example, those involved in the computation of detection *p* values.

Concerning the impact of genetic artifacts in the EWAS literature, except for CpGs affected by indels which are not always excluded in standard DNA methylation analysis, most CpGs under the influence of genetic artifacts can be found in previously published probe exclusion lists [6, 7]. However, we particularly raise alert on CpG sites affected by variants that yet remain unannotated in dbSNP, those influenced by several genetic artifacts or interacting with real biological variation whose manifestation can be particularly obscure. Overall, any in silico-predicted probe exclusion list will eventually contain false negatives, and hence, relying on them will not guarantee the complete exclusion of artifactual probes. This is particularly critical in studies dedicated to the interaction between genetics and epigenetics. At the same time, these lists may also contain false positives and, hence, result in the systematic exclusion of otherwise “healthy” probes. Such lists were crafted with limited mechanistic understanding and may never reach completeness due to blind spots in genetic variant calling and population-specific MAFs; they are constantly evolving with every new release of dbSNP (Additional file 1: Fig S24). Given all the above, we question why to use them at all; nowadays, the prolific availability of DNA methylation microarray data presents a unique opportunity for data-driven strategies. Interestingly, others have reached the same conclusion based on different grounds [36]. The whole point of having a microarray is to systematize the set of CpGs to be assayed but the reality is that different authors employ different probe exclusion schemes, resulting in substantial variation in the analysis of DNA methylation microarray data, greatly worsened by the long-lasting tendency for distributing only pre-processed data. Therefore, our final recommendation is to avoid excluding probes from epigenome-wide studies performed on Illumina DNA methylation microarrays, but instead to flag and heavily verify post hoc with data-driven tools such as UMtools, including raw data and annotations. Despite sacrificing statistical power due to the raised multiple testing burden, we believe that no information should be discarded when available, since it opens the door for unexpected discoveries. Also, including artifactual probes in EWAS can serve not only as a negative control but also as a GWAS proxy: sometimes, a genetic artifact- or meQTL-affected CpG may pop up as a hit in EWAS simply because the responsible genetic variant is in linkage equilibrium with a variant that is genuinely associated to the phenotype in question. For example, the methylation of cg01097406 and the nearby SNP rs154657 have both been found to be significantly associated with homocysteine levels via EWAS [56] and via GWAS [57], respectively (Additional file 1: Fig S25A). Subsequently, via meQTL mapping, these were also found to be significantly associated to each other [13]. In this case, the meQTL cg01097406 is likely acting as an allele-reporter for the putative *cis*-acting causal SNP rs8059821 (the only variant with matching MAF in AFR and EUR at chr16:89675000-89675250), which is in turn under linkage disequilibrium with rs154657 (Additional file 1: Fig S25B-E).

Additionally, we find important to discuss that none of the genetic variants discussed that disrupt a CpG site and disguise as U should be considered as genuine DNA methylation. It may be tempting to interpret that in these cases the Infinium assay measures a real outcome, since the targeted site cannot be methylated if it does not exist. However, being unable to distinguish whether a cytosine of interest is unmethylated or inexistent is of concern to any researcher. To solve this conundrum, we advocate for a locus-centric point of view: we define a genuine DNA methylation measurement when the methylation read-out is faithful to the expected methylation status of the region independently of the genetic template assayed. This way, if a CpG site is lost to a SNP and disguises as the unmethylated epiallele, the measured methylation status may not agree with the true methylation status of the region and, hence, can be considered as an artifact. Finally, we introduced a novel strategy to differentiate meQTLs from genetic artifacts based on co-methylation, which has been a common and recurrent issue in the literature [32, 58]. Though, our co-methylation strategy is based on two underlying assumptions: (i) nearby CpGs are available in the microarray at a reasonable distance to encounter co-methylation and (ii) nearby CpGs are not influenced by genetic artifacts themselves. Regarding the first assumption, and even though co-methylation drastically reduces after 1 kb, 70.8% of the 450K probes and 64.2% of the 850K probes have at least one neighbor within 500 bp (Additional file 1: Fig S26A). On the other hand, the second assumption is dramatically violated at regions enriched for SNPs, such as human leukocyte antigen (HLA) genes. At these regions, CpG/SBE-SNPs are so frequent that extensive networks of co-methylation are apparent not because of real biological correlation between the methylation at CpG sites, but in fact due to linkage disequilibrium between the SNPs causing the artifacts (Additional file 1: Fig S26B). In fact, we can observe this co-methylation between genetic artifacts also on the meQTL co-methylation plot outside the boundaries of the co-methylation window (colored in purple, Fig. 5B). Though we only validated one example meQTL here, we aim to automate our validation pipeline in a future meQTL-curating study.

Despite these limitations, our approach successfully in silico validated the meQTL at the *NINJ2* gene, for which we proposed a mechanistic model to explain the behavior at this locus. Identifying the particular ETS-TF involved would shed light to the biological mechanism, but this will be a hard task given the similarity in TFBSs between the 12 subfamilies of ETS-TF [59]. More importantly, we wonder whether the meQTL *cis*-causal variant identified, SNP rs34038797, is also a causal variant for associations identified in GWAS such as platelet/monocyte/lymphocyte counts. Fine-mapping results via the CausalDB database [60] highlight its potential as a trait-causal variant (Additional file 1: Fig S27). For the time being, we can only speculate about its potential mechanism. Albeit not much is known about *NINJ2*, its paralog ninjurin 1 (*NINJ1*), with whom it shares more than 50% identity, has important functions in axon regeneration upon nerve injury. NINJ1 is a membrane receptor with homophilic binding for which stable transfection results in the formation of large cellular aggregates [61]. We have shown that rs34038797 mediates an exon inclusion event that results in the extension of the N-terminal. Via transmembrane hidden Markov model (TMHMM) [62], we predicted that this N-terminal is extracellular and, hence, may mediate part of homophilic binding activity (Additional file 1: Fig S28). Thus, it is possible that the extension of NINJ2’s N-terminal is aberrant and that this is the mechanism by which GWAS-associated trait materialize. Particularly, *NINJ2* transcripts are 4.3 times more abundant in megakaryocytes than in erythroblasts [63]. Therefore, it is not farfetched to hypothesize that *NINJ2* may take a role in platelet function, explaining its association to decreased platelet characteristics observed in GWAS. However, we cannot discard that *NINJ2* may be involved in cellular communication and that lower counts in platelets, monocytes, and lymphocytes arise via alterations in the differentiation process itself.

To sum up, we have provided detailed classifications and examples that will aid researchers navigate through the huge diversity of genetic artifacts encountered on Illumina DNA methylation microarrays. Although aiming to uncover genetic artifacts, we have encountered a surprising amount of biological knowledge throughout this study, including sex differences, X-inactivation, imprinting, inter-tissue DNA methylation variation, co-methylation, and linkage disequilibrium. Albeit the richness in biological information of such probes, these are systematically excluded from current DNA methylation analysis. Based on our observations, we aim to inspire other researchers to explore innovative ways of using these probes in future microarray analysis. Lastly, we show that large-scale genetic variant calling from raw DNA methylation data is possible, which has noteworthy ethical implications, especially when combined with phenotypic/disease information; therefore, we invite close examination from bio-ethical experts.

## Conclusions

Our objective in this study was to build and validate a mechanistic understanding on how genetic artifacts influence DNA methylation quantification in Illumina DNA methylation microarrays as part of challenging current practices based on in silico-predicted probe exclusion lists. To achieve this, we created new data analysis tools to fully assess and characterize the presence of artifacts at the level of raw fluorescence data and we introduced monozygotic twins as genetic controls in our analyses. With our approach, we have provided detailed classifications and examples that will aid researchers navigate through the huge diversity of genetic artifacts encountered on Illumina DNA methylation microarrays. We additionally proposed a novel strategy based on co-methylation that can further discern between genetic artifacts and genuine genomic influence. Overall, our study sets the ground and proposes a paradigm shift on how to account for artifactual or genuine genomic influence on DNA methylation data; most notably, with implications for research dedicated to the heritability of DNA methylation and meQTL mapping.

## Methods

### Datasets

In this study, the following datasets were employed:

- (E-risk) Environmental Risk (E-risk) Longitudinal Twin Study (British, 450K (IDAT), GSE105018 (GEO), 426 MZ twin pairs, whole blood, samples collected at age 18 y, 48.6% females) [11, 64].
- (Small sample size dataset) Chinese children (Chinese, 450K (IDAT), GSE104812 (GEO), 48 samples, whole blood, mean age 9.04 y, 39.6% females) [65, 66].
- (C3ARE & GECKO) Cleaning, Carrying, Changing, Attending, Reading and Expressing (C3ARE) and Gene Expression Collaborative Kids Only (GECKO) cohorts (Canadian, 450K (IDAT), GSE124366 (GEO), 215 samples in total, of which 105 PBMC and 110 buccal cells, mean age 7.1 y and 47.9% females) [24, 67]. It includes 16 matched samples (same individual for both tissues) and technical replicates: 11 and 7 individuals were sampled in duplicates for buccal and PBMC, respectively.
- (ENID) Early Nutrition and Immune Development (ENID) Trial children cohort (Gambian, 450K (IDAT), GSE99863 (GEO), 240 children aged 2 years, whole blood, 48.6% females) [68, 69].
- (Isolated blood cell types). We combined the FACS-sorted blood profiles from 3 studies:
  - FlowSorted.Blood.450k (Swedish, 450K (IDAT), GSE35069 (GEO) 60 samples derived from whole blood of 6 healthy individuals, mean age 38 y, 100 % males) [70, 71].
  - FlowSorted.CordBlood.450k (American, 450K (rgSet), not available on GEO (solely via the R-package), 104 samples derived from cord blood of 17 individuals, 52.9 % female) [72].
  - FlowSorted.CordBloodNorway.450K (Norwegian, 450K (rgSet), not available on GEO (solely via the R-package), 77 samples derived from cord blood of 11 individuals, 54.5% females) [73].
- (MZ-Adipose*)* MuTHER cohort (British, 450K (GenomeStudio, M/U/detP), E-MTAB-1866 (ArrayExpress), 97 MZ twin pairs, subcutaneous adipose tissue, mean age = NA, 100% females) [74, 75].
- (Hematopoietic progenitors) Hematopoietic stem/progenitor cells (American, 450K (IDAT), GSE63409 (GEO), 74 samples derived from 20 individuals, variety of early hematopoietic progenitors in healthy and AML-individuals, mean age = NA, 40 % females) [76, 77].
- (Matched SNP/450K/WGBS) Matched genetic-epigenetic dataset [78].
  - SNP array data: GSE31438 [79], 14 samples
  - 450K data (GenomeStudio, M/U/detP): GSE33233 [80] and GSE30870 [81], 59 samples
  - WGBS data: GSE31263 [79], 3 samples. Same extra controls were also extracted from 7 non-CLL B-lymphocyte samples from GSE113336 [82, 83].
- (Twins WGBS) MuTHER study (British, whole-genome bisulfite sequencing (bed file), E-MTAB-3549 (ArrayExpress), 52 whole blood and adipose tissue samples belonging to 9 MZ and 8 DZ twin pairs, mean age 57.3 y, 100 % female) [75, 84]. The distribution of samples is the following: MZ-AT: MZ_1-7_; MZ-WB: MZ_1-5_, MZ_8-9_; DZ-AT: DZ_1-6_ and DZ-WB: DZ_1-4_, DZ_7-8_. Singletons were discarded.

### Data analysis

All data analysis was performed in R (https://www.r-project.org/) version 3.6.3 (“Holding the Windsock”) running on Ubuntu 18.04.4 LTS. Figures were created with R-base, lattice, ggplot2, and plotly R-packages. HiC-like co-methylation plots were generated by adapting scripts from the Sushi R-package. Bimodality coefficients were computed with functions from the modes R-package. The fitting of bivariate Gaussian mixture models was performed with functions from the EMCluster R-package. K-calling was performed with the dbscan algorithm implemented in the dbscan R-package. 450K and 850K information on positions and probes were obtained from the IlluminaHumanMethylation450kanno.ilmn12.hg19 and IlluminaHumanMethylationEPICanno. ilm10b4.hg19 R-packages. Cross-referencing of probes to dbSNP151 was performed with bedtools (v 2.29.2); more details can be found at Additional file 2. Phenotypic information was parsed from GEO with the GEOquery R-package. Raw intensity means and standard deviations were extracted from IDAT files with functions from the minfi and illuminaio R-packages.

### UMtools

The UMtools R-package containing all tools developed in this study and series of functions to ease the analysis of Illumina DNA methylation microarray raw fluorescence intensities will be available at Github and installable via devtools::install_github(“BenjaminPlanterose/UMtools”) together with a tutorial (https://github.com/BenjaminPlanterose/UMtools). Details on the definition and implementations on the employed tools can be found at Additional file 2. Briefly, U/M plots were simply the result of plotting the unmethylated against the methylated fluorescence intensity. Assignment of samples to clusters in the U/M plane (when the number of formed clusters is known) was performed with Gaussian mixture models via bGMM. CV_logT_ is measure of noise-to-signal ratio that was derived from the standard deviation of fluorescence across beads stored in the IDAT raw fluorescence intensity files as in:

$$ {CV}_{logT}\stackrel{\scriptscriptstyle\mathrm{def}}{=}\frac{1}{\log \left({\upmu}_{\mathrm{T}}\right)/\mathrm{R}-\mathrm{R}/2};\hat{R}=\frac{{\hat{\upsigma}}_{\mathrm{M}}+{\hat{\upsigma}}_{\mathrm{U}}+100}{\hat{\mathrm{U}}+\hat{\mathrm{M}}+100} $$

A bimodality coefficient was quantified from the sample skewness and kurtosis of CV_logT_ across samples for a given CpG. As a rule of thumb, BCs higher than 5/9 (the expected value of BC in a uniform distribution) point towards a bimodal or a multimodal distribution [28]. For genetic control, we also quantified CV_logT_ correlation between MZ twins for a given CpG. We established a conservative threshold of cor_MZ_(CV_logT_) = 0.8 for epigenome-wide genetic control purposes. Finally, K-calling was employed to automatically count the number of clusters in the U/M plane. This was performed via preprocessing of the signal and using the non-parametric clustering algorithm dbscan. However, dbscan requires calibration of two parameters: *minPts* and *eps*. To find the parameters *eps* and *minPts* that display the best performance at the E-risk cohort’s sample size, we calibrated dbscan in an independent training set composed of a total of 943 CpGs, forming one (*n* = 516), two (*n* = 205), three (*n* = 212), or four (*n* = 10) clusters. This set of markers was built by manually curating U/M plots from random CpGs. We then selected parameters to optimize K-calling, written as a multi-class classification machine learning task scored by a macro F_1_-score using categories *K* = [1–3]. The final parameters employed were *minPts* = 12 and *eps* = 0.035.

### UMtools benchmarking

In the benchmarking of UMtools, we aimed to include probes targeting the Y-chromosome (*n* = 416), the X-chromosome (*n* = 11,232), and control probes targeting SNPs (*n* = 65). We excluded, however, known cross-reactive probes [6, 7], probes containing SNPs at the CpG/SBE site, and probe-SNPs with MAF > 0.01, based on the SNP.147CommonSingle annotation file available at the IlluminaHumanMethylation450kanno.ilmn12.hg19 R-package. Also, to quantify the performance of UMtools, we employed the following set of conservative scores: correct assignment coefficient: $\frac{1}{\mathrm{n}}\sum \limits_{\mathrm{i}=1}^{\mathrm{n}}{\uprho_i}_{\mathrm{MZ}\ \mathrm{assigned}\ \mathrm{cluster}}^2$, genetics-related probe failure: $\frac{1}{\mathrm{n}}\sum \limits_{\mathrm{i}=1}^{\mathrm{n}}{\mathbf{1}}_{{\mathrm{BC}}_i\left({\mathrm{CV}}_{\mathrm{logT}}\right)>5/9}\cdotp {\mathbf{1}}_{{\mathrm{cor}}_i\left({\mathrm{CV}}_{\mathrm{logT}}\right)>0.8}$ and correct cluster number prediction:$\frac{1}{\mathrm{n}}\sum \limits_{\mathrm{i}=1}^{\mathrm{n}}{\mathbf{1}}_{{\mathrm{k}}_{\mathrm{i}}={\mathrm{K}}_{\mathrm{Exp}}}$, where **1**_*condition*_ is the indicator function, equal to one when condition is met. The logic of each score is further discussed in detail at Additional file 2. In the comparison with existing tools, we computed detection *p* values with minfi::detectionP, EWAStools::detectionP and sesame::pOOBAH. Also, we deployed gaphunter with minfi::gaphunter using default parameters: threshold = 0.05, keepOutliers = FALSE and oneCutoff = 0.01.

### 450K microarray and WGBS data preprocessing

Throughout analysis, we deliberately kept the preprocessing to the minimum to showcase that raw data of Illumina DNA methylation microarrays can be as interpretable as highly processed data. To this end, U/M plots, CV_logT_ and BC(CV_logT_) computations, K-calling, and MAF estimation were performed with unnormalized fluorescence signals, as registered in the IDAT files. However, at some stages, preprocessing was necessary. For R^2^_MZ_ vs genomic coordinate and co-methylation plots, we first computed *M*-values as in ${\log}_2\left(\frac{M+1}{U+1}\right)$; we preferred *M*-values to methylation ratio as they are unbounded and, hence, better equipped for correlation computation. Subsequently, we used preprocessCore::normalize.quantiles to perform quantile normalization (QN) on the *M*-value matrix, as MZ twin pairs in the E-risk cohort were placed on the same chip. Without QN, spurious correlations generated by batch effects raised the background R^2^_MZ_ substantially; this effect is well known, and it actually motivated the adaptation of QN in microarray analysis [85]. For methylation heatmaps, we computed methylation ration as in $\beta =\frac{M}{M+U+100}$, which we also pre-processed with QN. In the case of the NINJ2 meQTL confirmation via WGBS data on MZ twins, we followed the same minimum-preprocessing logic. We displayed the DNA methylation status of all positions at the windows chr12:673461-772946 and chr12:739280-740338, regardless of the coverage.

### Statistical analysis of genetic artifacts

The identification of examples and the computation of MAF is described in great detail on Additional file 2. For the epigenome-wide statistical analysis of CpG/SBE-SNPs, we began from the .vcf files outputted by bedtools; filtering out indels, the following CpG (*n* = 16,724) and SBE sites (*n* = 562) remained. We filtered variants with MAF < 0.05, excluded triallelic SNPs and classified a total of 7722 CpGs into the 16 categories registered at Table 2. Additionally, to reduce the noise of other sources of variation, we removed CpGs in these lists that were also associated to CpG/SBE/probe-indels, probe-SNPs at a distance of ≤ 5 bp from the 3′-end, CpGs with multiple CpG/SBE-SNPs, CpGs mapping to chromosome X or Y, and probes known to be cross-reactive. Finally, given that our epigenome-wide assessment tools have different detection sensitivities and that these also depend on sample size, we limited the analysis to variants with MAF > 0.1 for BC(CV_logT_) and cor_MZ_(CV_logT_) resulting in 4102 probes, or with MAF > 0.3 for the K-caller resulting in 1433 probes. Differences in BC(CV_logT_) and cor_MZ_(CVl_ogT_) between groups were assessed with linear models. K-calling differences were assessed via ternary plots.

For the epigenome-wide statistical analysis of probe-SNPs, we began from the .vcf file outputted by bedtools for probe-genetic variants (*n* = 103,728). We removed CpGs associated to indels, associated to triallelic SNPs or SNPs with MAF < 0.01. Additionally, to reduce the noise of other sources of variation, we removed CpGs in these lists that were also associated to CpG/SBE/probe-indels, CpG/SBE-SNPs, CpGs associated to multiple probe-SNPs, those mapping to chromosome X or Y, and probes known to be cross-reactive. Again, we also limited the analysis to variant with MAF > 0.1 for BC(CV_logT_) and cor_MZ_(CV_logT_), resulting in 48,656 probes, or with MAF > 0.3 for the K-caller, resulting in 8640 probes. BC(CV_logT_) and cor_MZ_(CVl_ogT_) as a function of the distance to the 3′-end was assessed with a generalized linear model of the gamma family with a log link function. To quantify potential bleed through of artifactual probes in the published literature, we had to build an artifactual set of probes via a pipeline as highlighted in Additional file 1: Fig S14, aiming to be as conservative as possible.

### Verification of the NINJ2 meQTL

Co-methylation was computed with the cor function with method = “pearson” and visualized with the plotHic function from the Sushi R-package. To compute the MAF of the meQTL, we employed all CpGs within the meQTL. To do so, we performed hierarchical clustering on the matrix of Euclidean distances with the hclust and dist functions and cut the dendrogram with the function cutree with *k* = 3. Also, in the analysis of matched 450K and SNP data, given that our target SNP was not included in the SNP array design, we had to impute it from nearby SNPs, by making use of Impute2 v2.3.2 [86]. Finally, TFBS discovery was performed with the motifbreakR R-package [40]. We ran motifbreakR for rs34038797 against DNA motif databases HOMOCO, ENCODE, HOMER, FactorBook, with arguments filterp = T, threshold = 1e−4, method = “ic” and bkg = c(A = 0.25, C = 0.25, G = 0.25, T = 0.25). The motifbreakR output was visualized with plotMB, with arguments rsid = “rs34038797” and effect = “strong.” Additionally, we consulted the SNP2TFBS (SNPviewer) [41], ChIPSummitDB [42] QTLbase [48], CausalDB [60], and TMHMM [62] web services.

## Supplementary Information

**Additional file 1.** Supplementary figures.

**Additional file 2.** Supplementary methods.

**Additional file 3.** Review history.

## Data Availability

All datasets employed in this study are publicly available. Accession identifiers are listed here: E-risk (GSE105018, GEO) [11], Small sample size (GSE104812; GEO) [65], C3ARE & GECKO (GSE124366; GEO) [24], ENID (GSE99863; GEO) [68], Isolated blood cell types (FlowSorted.Blood.450k [70], FlowSorted.CordBlood.450k [72], FlowSorted.CordBloodNorway.450K [73]; R-packages), Adipose tissue in MZ twins (E-MTAB-1866; ArrayExpress) [74], hematopoietic progenitors (GSE63409; GEO) [76], Matched SNP/450K/WGBS and additional controls (GSE31438, GSE33233, GSE30870, GSE31263, GSE113336; GEO) [78, 82] and Twins WGBS (E-MTAB-3549; ArrayExpress) [84]. The UMtools R-package is available under an MIT license together with a tutorial at GitHub (https://github.com/BenjaminPlanterose/UMtools) [87]. A current release has also been deposited at the Zenodo digital object identifier-assigning repository (10.5281/zenodo.5055529) [88].
