# Supplementary material for: Revisiting genetic artifacts on DNA methylation microarrays exposes novel biological implications
Source: Genome Biol. 2021 Sep 21;22:274. doi: 10.1186/s13059-021-02484-y (PMC8454075; doi:10.1186/s13059-021-02484-y)
Supplement: Supplementary file 1 — Additional file 1. Supplementary figures. [file 13059_2021_2484_MOESM1_ESM.pdf]

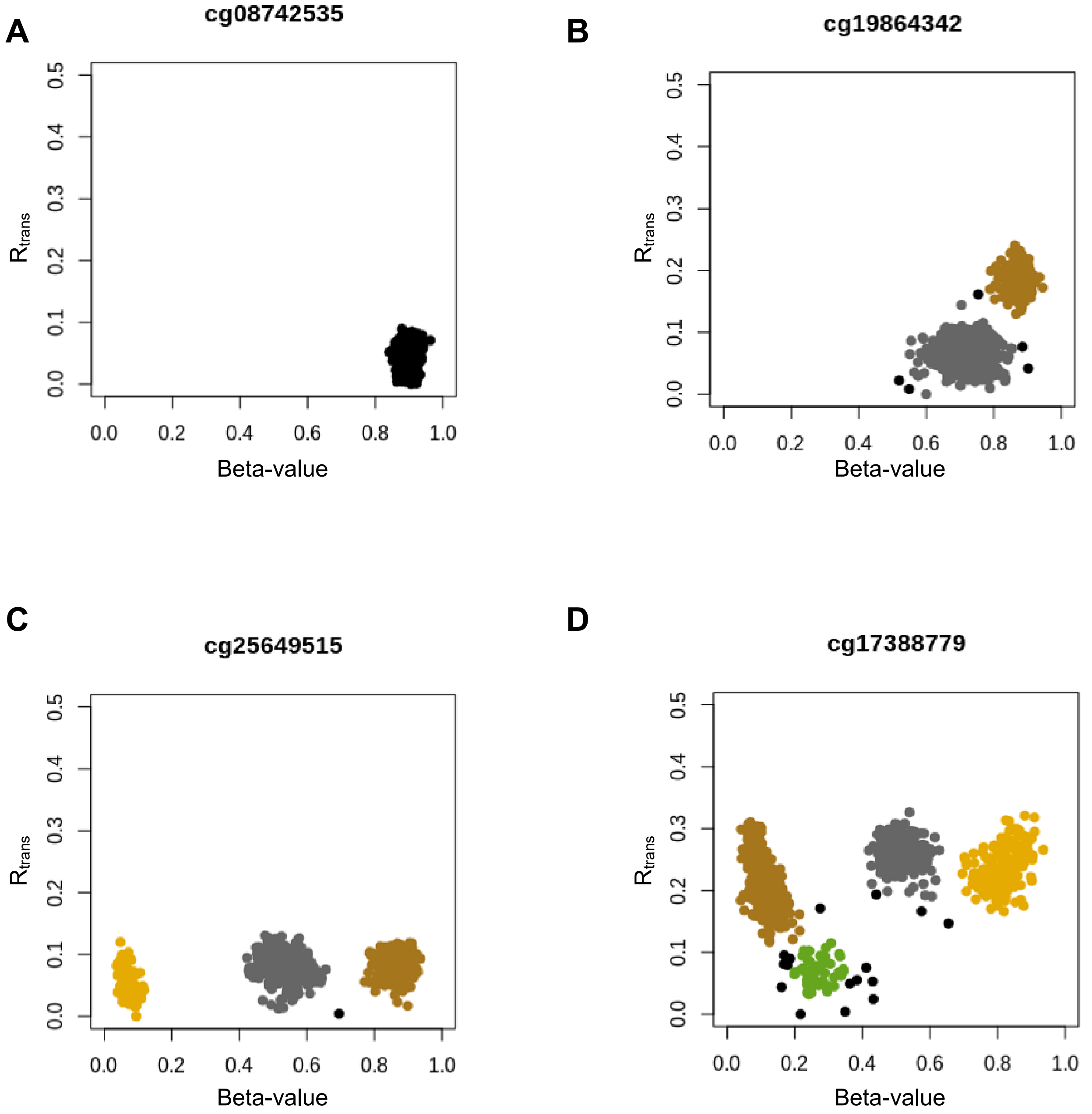

Figure S1. K-caller output visualization for a set of representative examples forming varying number of clusters in the U/M plane on the E-risk cohort. (A) K=1. (B) K=2. (C) K=3. (D) K=4. Black points in B, C and D represent outliers, samples that do not belong to any cluster as per dbscan criterion. The coordinate transformation employed is described in detail on Additional file 2.

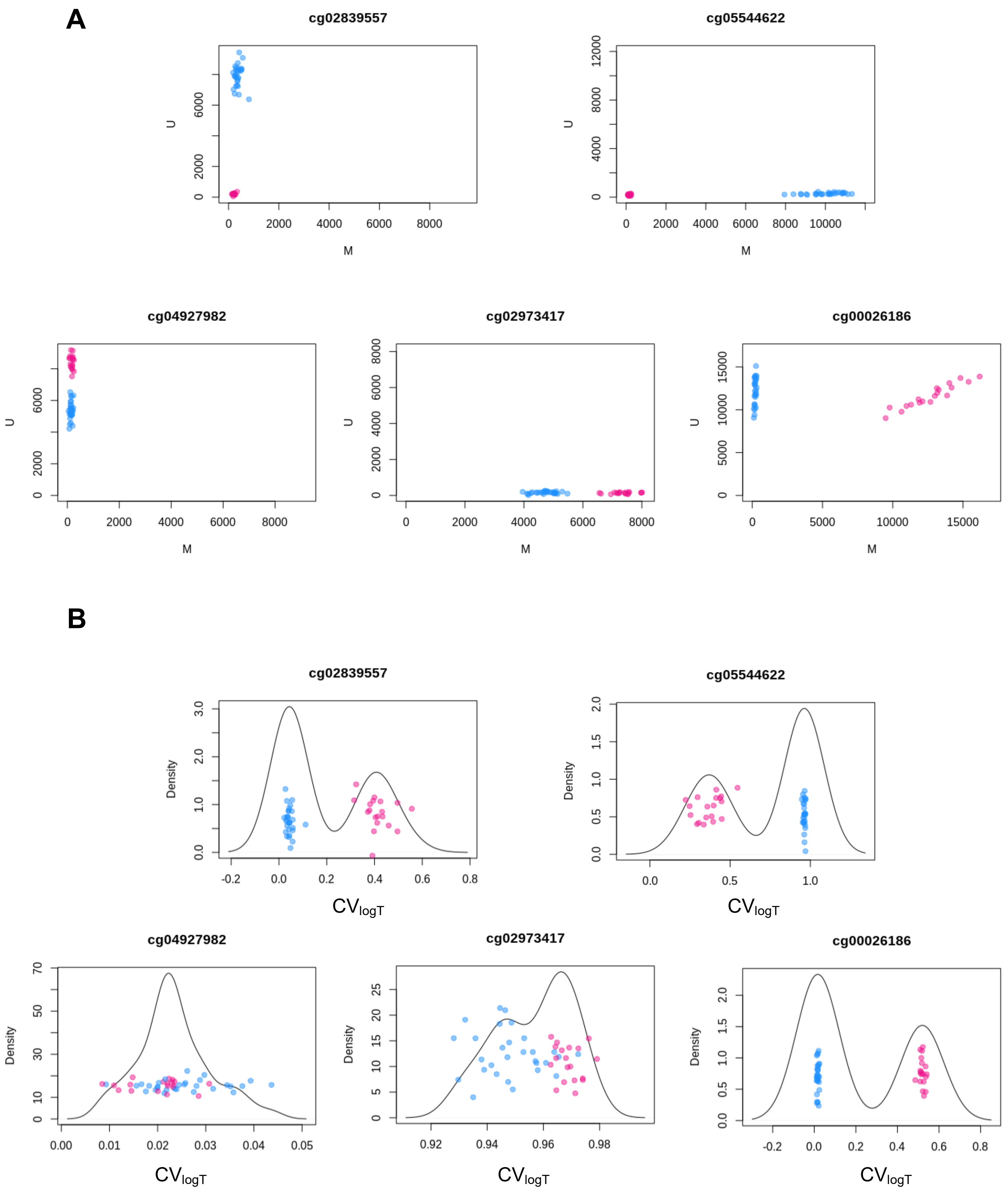

Figure S2. Behavior of sex-chromosome targeting probes at a cohort with a low sample size (n = 48). (A) U/M plots and (B) CV_{logT} distribution for examples highlighted in Figure 1D.

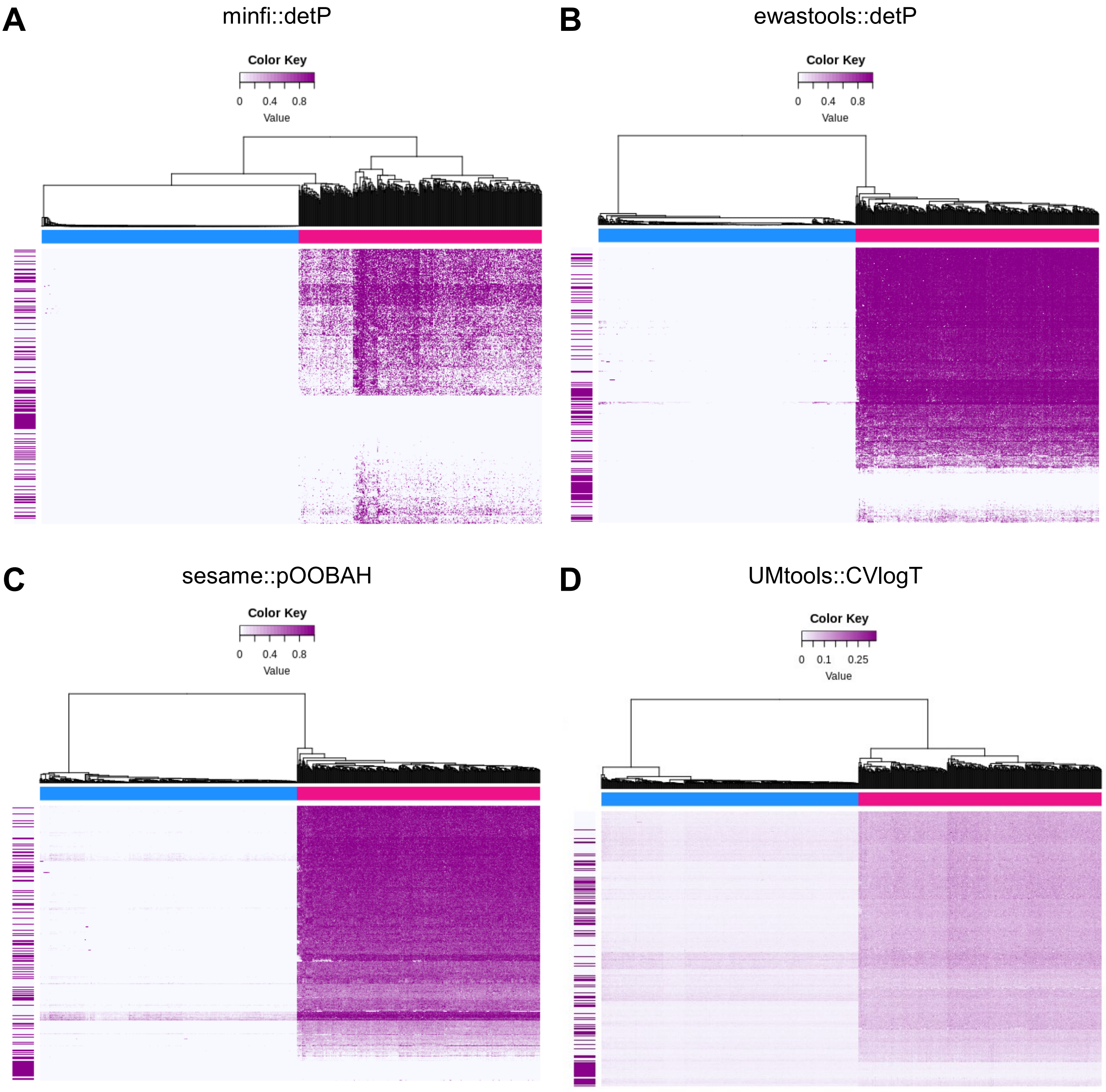

Figure S3. Comparison between detection p-values and BC(CV_{logT}). Heatmap for (A) minfi::detP, (B) ewastools::detP, (C) sesame::pOOBAH and (D) UMtools::CVlogT. As column colours, males and females are highlighted in blue and pink, respectively. As row colours, known cross-reactive probes have been highlighted in purple.

A

Training set

minfi::gaphunter, thr = 0.05, outCutoff=0.01,						
	**PREDICTED**					
**TRUE**	**K = 1**	**K = 2**	**K = 3**	**K = 4**	**K = 5**	**K = 6**
**K= 1**	515	1	0	0	0	0
**K= 2**	75	85	38	7	0	0
**K = 3**	2	28	131	45	5	1
**K = 4**	1	4	3	2	0	0

For K = {1, 2, 3}
macroPrecision = 0.797
macroRecall = 0.747
macroF1 = 0.756

UMtools::Kcaller, minPts = 12; eps = 0.035				
	**PREDICTED**			
**TRUE**	**K = 1**	**K = 2**	**K = 3**	**K = 4**
**K= 1**	515	1	0	0
**K= 2**	9	193	3	0
**K = 3**	0	8	204	0
**K = 4**	0	0	3	7

For K = {1, 2, 3}
macroPrecision = 0.975
macroRecall = 0.967
macroF1 = 0.971

B

Testing set

		ChrY	$ChrX_{inact}$	$ChrX_{hypermeth + escape}$	SNP-probes
Markers	# probes	266	3,981	3,028	65
	Expected K	2	2	1 if high sample size	3
	Probe failure in fe-males	Yes	No	No	No
UMtools					
K-calling	Correct # clusters predicted	0.977	0.902	0.999	1.000
Gaphunter	Correct # clusters predicted	0.169	0.559	0.996	N/A

Figure S4. Comparison between Gaphunter and K-caller. Performance metrics on (A) the training set and (B) the testing set. Comparison should be made based solely on the testing set as the K-caller has been optimized on that given training set. As K-caller employs raw intensity signals, we supplied gaphunter with the output of minfi::preprocessRaw for fair comparison.

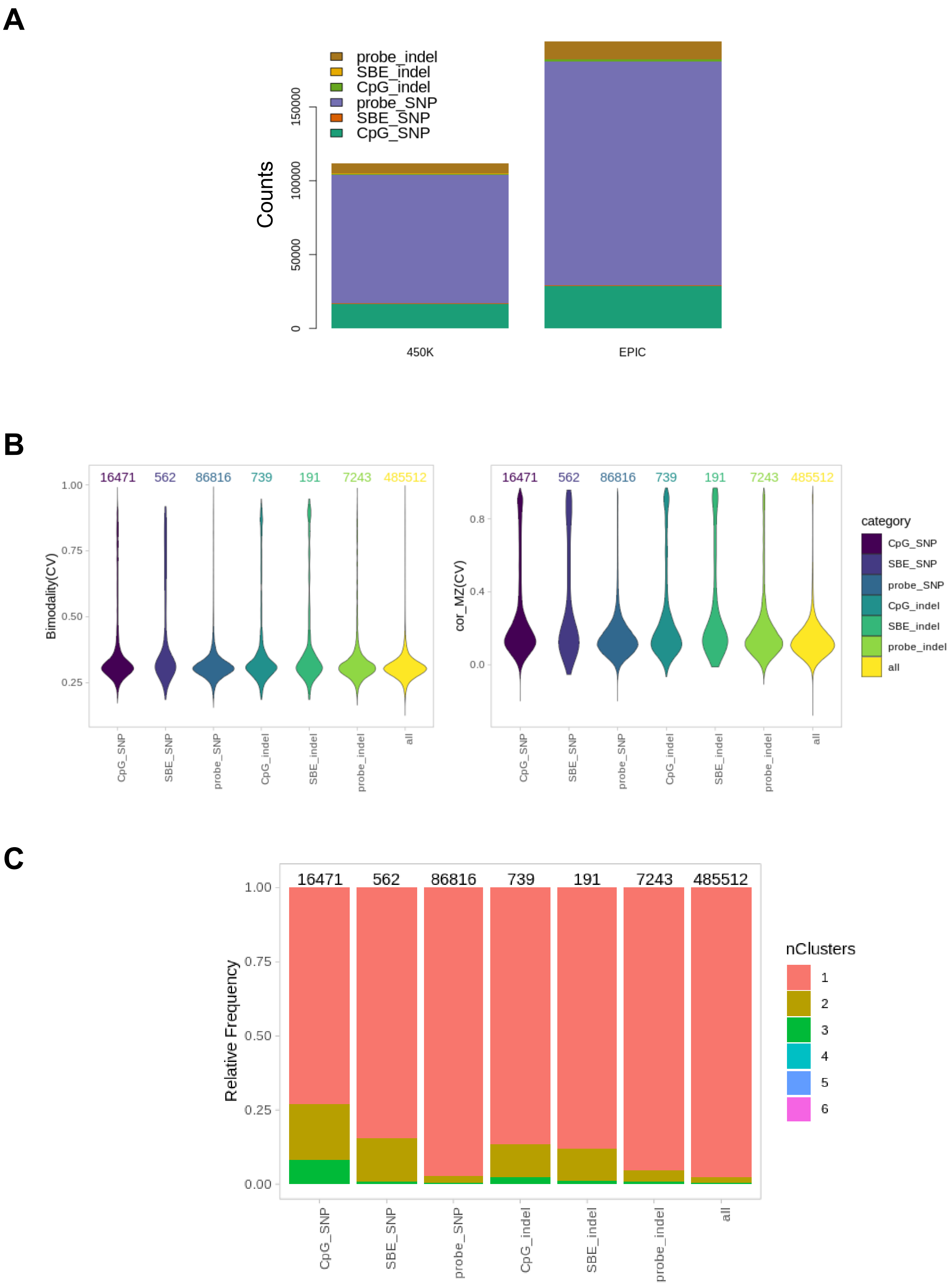

Figure S5. Overview of annotated genetic variants in dbSNP151. (A) Probe count associated to genetic artefact categories in the 450K and the EPIC. (B) $BC(CV_{logT})$ and $cor_{MZ}(CV_{logT})$ distribution and (C) number of clusters called by K-caller across categories in the E-risk cohort. Total number of probes included in each category is highlighted on top of each violin or bar plot.

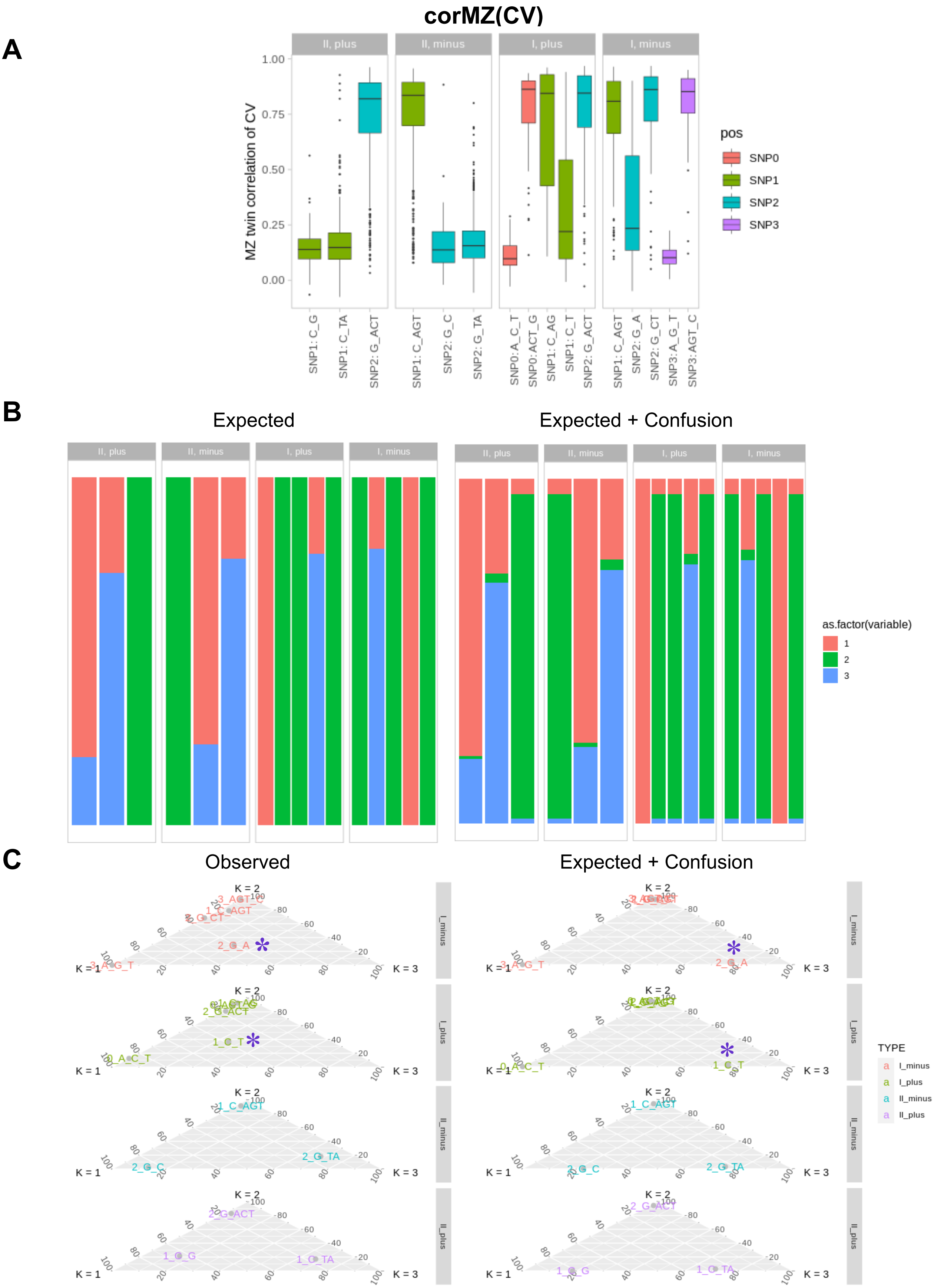

Figure S6. Additional controls for CpG/SBE-SNPs. (A) Distribution of $cor_{MZ}(CV_{logT})$ across categories. (B) Expected distribution of K-calls with and without K-calling confusion correction. (C) Ternary plot representation of K-calling distribution for observed distribution and expected distribution corrected for confusion of the K-caller. The two categories that violate expectations are highlighted with a purple asterisk.

A

	**Estimate**	**Std. Error**	**t value**	**Pr(>|t|)**
(Intercept)	0.307946	0.016934	18.185	< 2e-16 ***
L1I_minus_SNP1_C_AGT	0.362207	0.020213	17.919	< 2e-16 ***
L1I_minus_SNP2_G_A	0.090616	0.019831	4.569	5.03e-06 ***
L1I_minus_SNP2_G_CT	0.397874	0.024337	16.348	< 2e-16 ***
L1I_minus_SNP3_A_G_T	-0.001648	0.029799	-0.055	0.956
L1I_minus_SNP3_AGT_C	0.391824	0.026140	14.989	< 2e-16 ***
L1I_plus_SNP0_A_C_T	0.012914	0.033123	0.390	0.697
L1I_plus_SNP0_ACT_G	0.384054	0.025539	15.038	< 2e-16 ***
L1I_plus_SNP1_C_AG	0.353048	0.027653	12.767	< 2e-16 ***
L1I_plus_SNP1_C_T	0.099491	0.021120	4.711	2.55e-06 ***
L1I_plus_SNP2_G_ACT	0.390025	0.020357	19.159	< 2e-16 ***
L1II_minus_SNP1_C_AGT	0.370059	0.017775	20.819	< 2e-16 ***
L1II_minus_SNP2_G_C	0.004964	0.021839	0.227	0.820
L1II_minus_SNP2_G_TA	0.012474	0.017404	0.717	0.474
L1II_plus_SNP1_C_TA	0.010493	0.017830	0.588	0.556
L1II_plus_SNP2_G_ACT	0.366566	0.017696	20.715	< 2e-16 ***

REFERENCE: "II_plus_SNP1_C_G"
Residual standard error: 0.1365 on 4086 degrees of freedom
Multiple R-squared: 0.6231, Adjusted R-squared: 0.6218
F-statistic: 450.4 on 15 and 4086 DF, p-value: < 2.2e-16

B

	**Estimate**	**Std. Error**	**t value**	**Pr(>|t|)**
(Intercept)	0.14701	0.02161	6.803	1.18e-11 ***
L1I_minus_SNP1_C_AGT	0.59024	0.02580	22.881	< 2e-16 ***
L1I_minus_SNP2_G_A	0.19035	0.02531	7.521	6.63e-14 ***
L1I_minus_SNP2_G_CT	0.61005	0.03106	19.641	< 2e-16 ***
L1I_minus_SNP3_A_G_T	-0.03892	0.03803	-1.023	0.306
L1I_minus_SNP3_AGT_C	0.63494	0.03336	19.032	< 2e-16 ***
L1I_plus_SNP0_A_C_T	-0.03410	0.04227	-0.807	0.420
L1I_plus_SNP0_ACT_G	0.61697	0.03259	18.930	< 2e-16 ***
L1I_plus_SNP1_C_AG	0.5464	0.03529	15.485	< 2e-16 ***
L1I_plus_SNP1_C_T	0.18359	0.02695	6.811	1.11e-11 ***
L1I_plus_SNP2_G_ACT	0.61938	0.02598	23.840	< 2e-16 ***
L1II_minus_SNP1_C_AGT	0.60521	0.02269	26.679	< 2e-16 ***
L1II_minus_SNP2_G_C	0.01121	0.02787	0.402	0.688
L1II_minus_SNP2_G_TA	0.02157	0.02221	0.971	0.332
L1II_plus_SNP1_C_TA	0.01798	0.02276	0.790	0.430
L1II_plus_SNP2_G_ACT	0.59762	0.02258	26.462	< 2e-16 ***

REFERENCE: "II_plus_SNP1_C_G"
Residual standard error: 0.1742 on 4086 degrees of freedom
Multiple R-squared: 0.7262, Adjusted R-squared: 0.7252
F-statistic: 722.5 on 15 and 4086 DF, p-value: < 2.2e-16

Figure S7. Linear models of (A) BC(CV_{logT}) and (B) cor_{MZ}(CV_{logT}) as a function of the CpG/SBE-SNP categories.

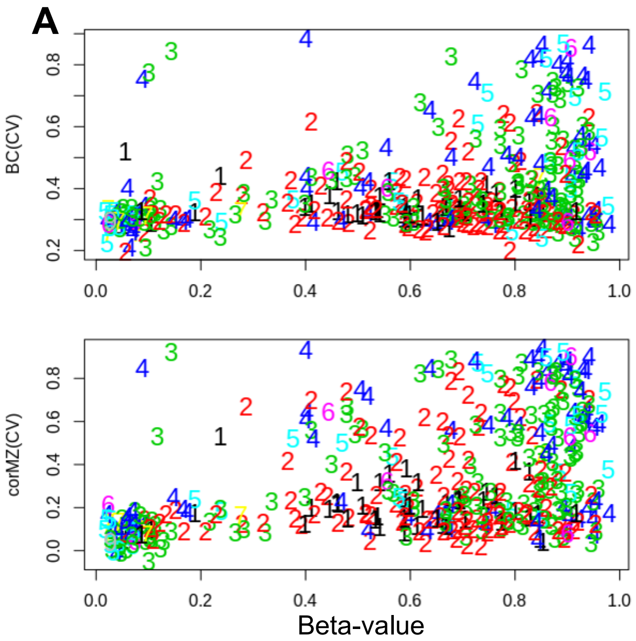

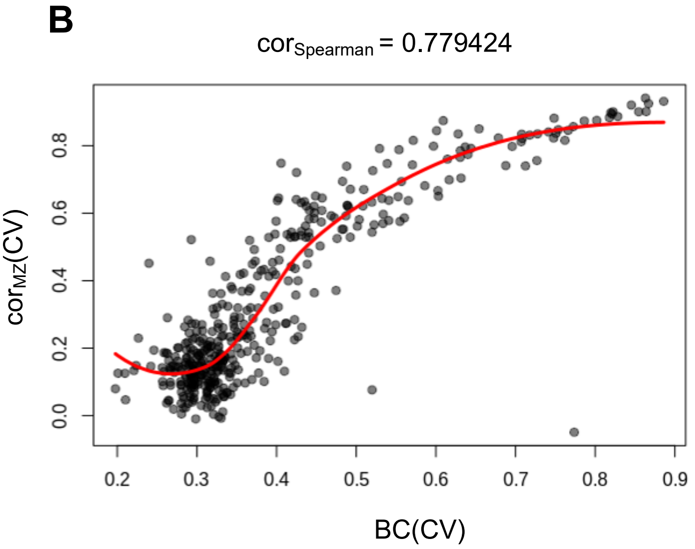

C

	Estimate	Std. Error	t value	Pr(>|t|)
(Intercept)	0.371384	0.027230	13.639	< 2e-16 ***
I_{meth}	-0.093340	0.033430	-2.792	0.00548 **
nCpG	-0.007638	0.007462	-1.023	0.30668
I_{meth}:nCpG	0.054812	0.009776	5.607	3.73e-08 ***

Residual standard error: 0.1297 on 421 degrees of freedom
Multiple R-squared: 0.1585, Adjusted R-squared: 0.1525
F-statistic: 26.43 on 3 and 421 DF, p-value: 1.11e-15

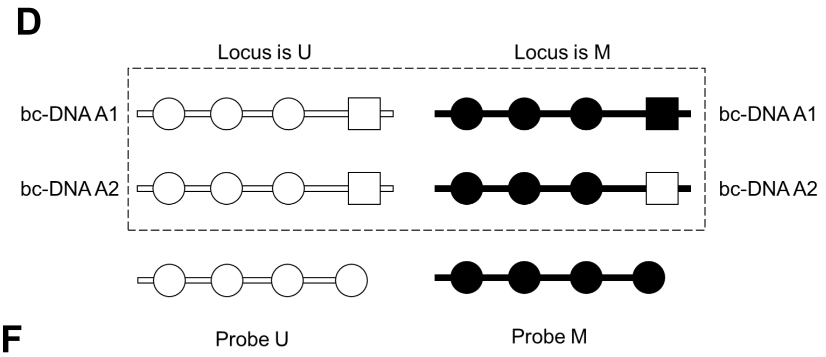

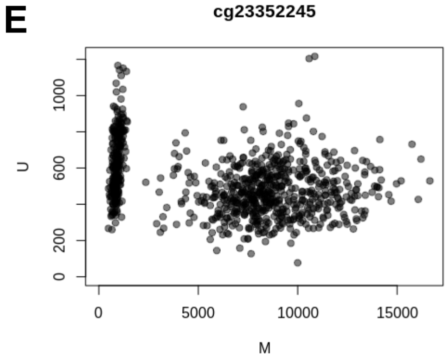

F

cg23352245

```
5'-TGGGTGGGCGCAGGCTTGGCGGGCCCCACAGTCGGAGCTGCCGTCAGG[C G/A]CCACCAGCC-3' (gDNA)
3'-ACCCACCCGCGTCCGAACCGCCCGGGGTGTCAGCCTCGACGGCAGTCC[G C/T]GGTGGTCGG-5' (cDNA)
3'-ATTTATTTGYGTTTGAATTGYTTGGGGTGTTAGTTTTGATGGYAGTTT[G Y/T]GGTGGTCGG-5' (bc-cDNA)

5'-TAAATAAACACAAACTTAACAAACCCCACAATCAAAACTACCATCAAA[C A] (probe T-I U)
5'-TAAATAAACGCAAACTTAACGAACCCCACAATCGAAACTACCGTCAAA[C G] (probe T-I M)
```

Figure S8. Modelling type-I (+) SNP1: C↔T and type-I (−) SNP2: G↔A categories (n = 425). (A) $BC(CV_{logT})$ and $cor_{MZ}(CV_{logT})$ as a function of the average methylation of nearby CpGs. Digits within the plot window symbolize the total number of CpGs assessed by the probe. (B) The non-linear correlation between $BC(CV_{logT})$ and $cor_{MZ}(CV_{logT})$ as evidence of genetic control. (C) Linear model testing the interaction between methylation status and number of CpGs targeted by the probe. (D) Graphical model on the behavior of these two categories. (E) A highlighted example and (F) its sequence context with 3 internal CpGs.

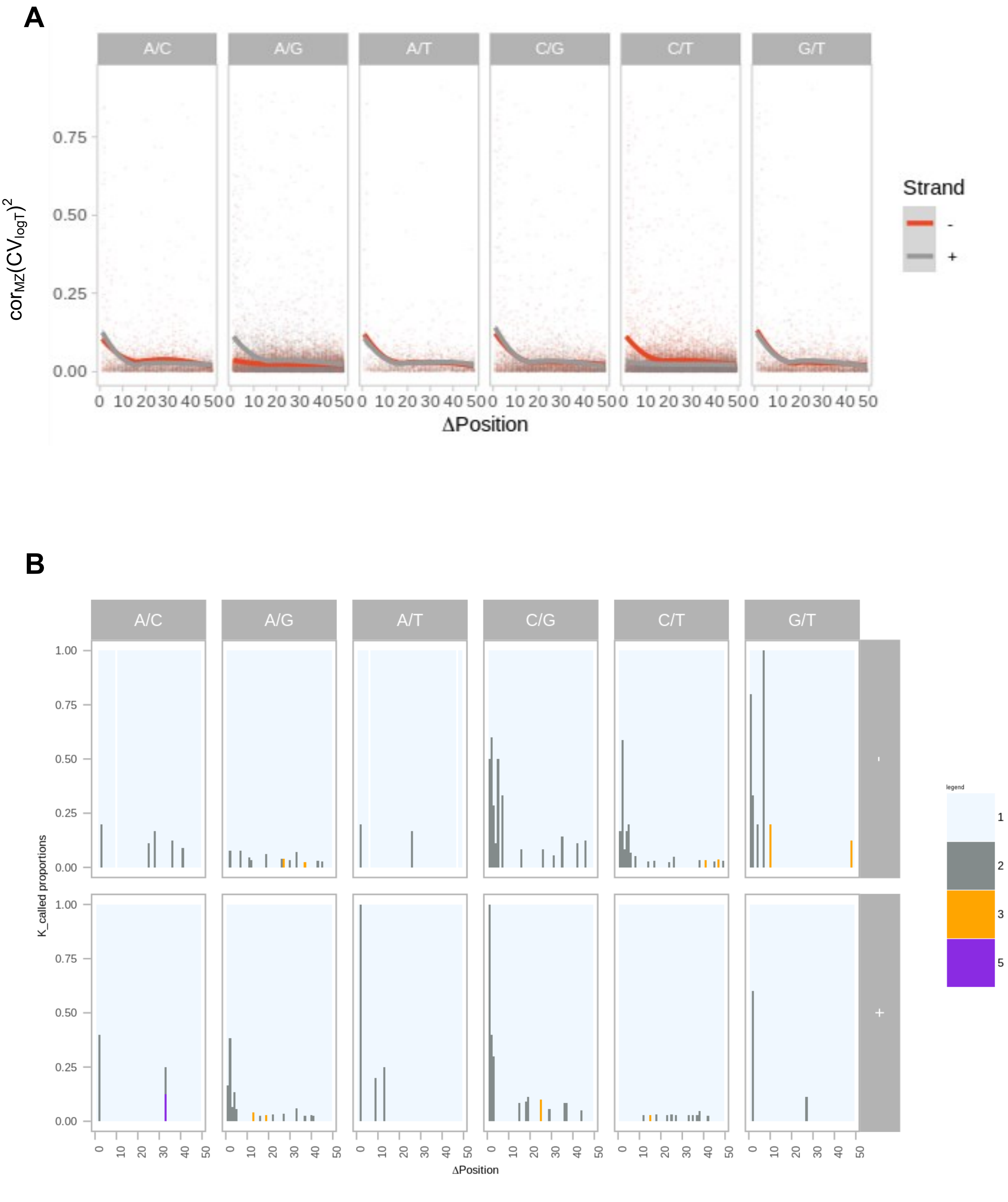

Figure S9. Additional controls for probe SNPs. (A) $cor_{MZ}(CV_{logT})^2$ and (B) number of clusters called by K-caller as a function of strand, SNP alleles and distance to the 3'-end of the probe.

A

BC(CV_{logT})

	Estimate	Std. Error	t value	Pr(>|t|)
(Intercept)	-1.120E+-00	5.20E-03	-215.406	< 2e-16 ***
delta	-9.96E-04	6.75E-05	-14.768	< 2e-16 ***
- A/G	-2.71E-02	5.38E-03	-5.033	4.85E-07 ***
- A/T	-6.05E-03	8.18E-03	-0.739	0.4597
- C/G	-5.92E-03	6.28E-03	-0.944	0.3452
- C/T	-1.61E-03	5.38E-03	-0.299	0.7652
- G/T	-2.11E-03	6.77E-03	-0.312	0.7551
+ A/C	-1.26E-02	6.67E-03	-1.891	0.0587
+ A/G	-6.38E-03	5.33E-03	-1.196	0.2315
+ A/T	-5.46E-03	7.74E-03	-0.705	0.4806
+ C/G	-5.09E-04	6.17E-03	-0.082	0.9343
+ C/T	-2.36E-02	5.34E-03	-4.426	9.61E-06 ***
+ G/T	-8.39E-03	6.82E-03	-1.231	0.2181

REF = "- A/C"
(Dispersion parameter for Gamma family taken to be 0.04075982)
Null deviance: 1453.0 on 48655 degrees of freedom
Residual deviance: 1438.7 on 48643 degrees of freedom
AIC: -147163
Number of Fisher Scoring iterations: 4

B

cor_{MZ}(CV_{logT})

	Estimate	Std. Error	t value	Pr(>|t|)
(Intercept)	-2.86643	0.046495	-61.65	<2e-16 ***
delta	-0.0173	0.000603	-28.685	<2e-16 ***
- A/G	-0.40052	0.048065	-8.333	<2e-16 ***
- A/T	-0.14337	0.073154	-1.96	0.05 .
- C/G	-0.0555	0.056094	-0.989	0.3225
- C/T	0.015951	0.04806	0.332	0.74
- G/T	-0.11202	0.060484	-1.852	0.064
+ A/C	-0.19166	0.059612	-3.215	0.0013 **
+ A/G	-0.01112	0.047666	-0.233	0.8156
+ A/T	-0.11947	0.069136	-1.728	0.084 .
+ C/G	-0.04753	0.055152	-0.862	0.3888
+ C/T	-0.40604	0.047723	-8.508	<2e-16 ***
+ G/T	-0.06528	0.060924	-1.071	0.284

REF = "- A/C"
(Dispersion parameter for Gamma family taken to be 3.25651)
Null deviance: 92428 on 48655 degrees of freedom
Residual deviance: 87412 on 48643 degrees of freedom
AIC: -246974
Number of Fisher Scoring iterations: 7

Figure S10. Generalized linear models of the Gamma family with log link function of (A) BC (CV_{logT}) and (B) cor_{MZ}(CV_{logT}) as a function of the probe-SNP categories.

indel = Probe failure

A

Type II, strand (–), delC

```
  5'-GACCA[(C) G]TTCGA-3'  (gDNA)
  3'-CTGGT[(G) C]AAGCT-5'  (cDNA)
  3'-TTGGT[(G) Y]AAGYT-5'  (BC cDNA)
5'——AACCA[ C]              (probe, II)
```

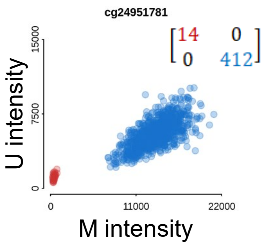

rs34833685

Study	freq (C)	freq (delC)
EUR, 1000 genomes n = 1006	0.856	0.144
E-risk n = 426	0.819	0.181

ins = U

B

Type II, strand (–), insA

```
  5'-GGCAC[C(A) G]TCACA-3'  (gDNA)
  3'-CCGTG[G(A) C]AGTGT-5'  (cDNA)
  3'-TTGTG[G(A) Y]AGTGT-5'  (BC cDNA)
5'——AACAC[C]                (probe, II)
```

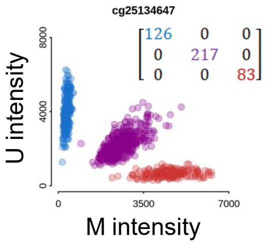

rs11382510

Study	del(A)	ins(A)
EUR, 1000 genomes n = 1006	0.430	0.570
E-risk n = 426	0.450	0.550

ins = M

C

Type I^{Green}, strand (-), delGCGACCCGCCG(TCC)$_2$CATCC / dupGCGACCCGCCG(TCC)$_2$CATCC

```
5'-CCATC[C(G]CGACCCGCCGTCCTCCCATCC)AC-3'   (gDNA)
3'-GGTAG[G(C]GCTGGGCGGCAGGAGGGTAGG)TG-5'   (cDNA)
3'-GGTAG[G(Y]GYTGGGYGGYAGGAGGGTAGG)TG-5'   (BC-cDNA)
——CCATC[C A]                              (probe U, I^Grn)
——CCATC[C G]                              (probe M, I^Grn)
```

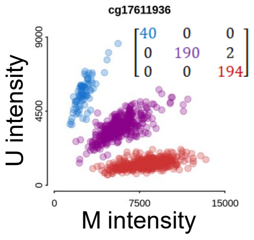

rs71827935

Study	freq (del)	freq (ins)
EUR, 1000 genomes n = 1006	0.245	0.755
E-risk n = 426	0.318	0.682

indel = change of detection channel

D

Type I^{Red}, strand (+), delGCACC

```
5'-GCGCAC[C(G]CACC)C-3'  (gDNA)
5'-GYGTAT[Y(G]TATT)Y-3'  (BC gDNA)
       [A C]ATAA A——     (probe U, I^Red)
       [G C]ATAA G——     (probe M, I^Red)
```

```
5'-GCGCAC[C]C-3'  (gDNA)
5'-GYGTAT[Y]T-3'  (BC gDNA)
   ACATA A A——    (probe U, I^Red)
   GCATA A G——    (probe M, I^Red)
```

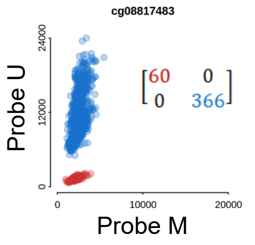

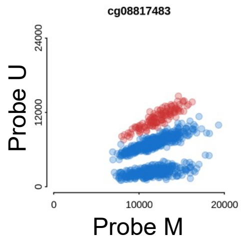

rs202145474

Study	freq (ins)	freq (del)
EUR, 1000 genomes n = 1006	0.633	0.367
E-risk n = 426	0.625	0.375

indel = no genetic artefact

E

Type I^{Red}, strand (+), delTC

```
5'-CCCY(T[C)G]CGCCC-3'  (gDNA)
5'-TTTY(T[Y)G]YGTTY-3'  (BC gDNA)
      [A C]ACAAA——      (probe U, I^Red)
      [G C]GCAAG——      (probe M, I^Red)
```

rs570527436, f(delTC) = 0.551

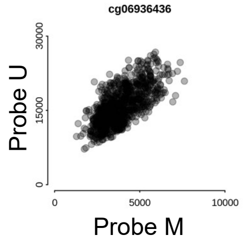

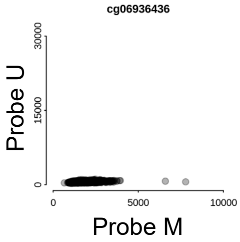

Figure S11. Examples of CpG-indel with a variety of manifestations. Indel disguising as (A) probe failure, (B) U epiallele and (C) M epiallele. (D) SNP at a SBE site of a type-I probe causing detection channel switch or (E) not. Allelic frequencies were estimated based on UM plots via bGMM; only concordant monozygotic twin pairs were taken into account in the computation. Confusion matrices are also available on the plots. Probe designs are highlighted in yellow boxes.

A

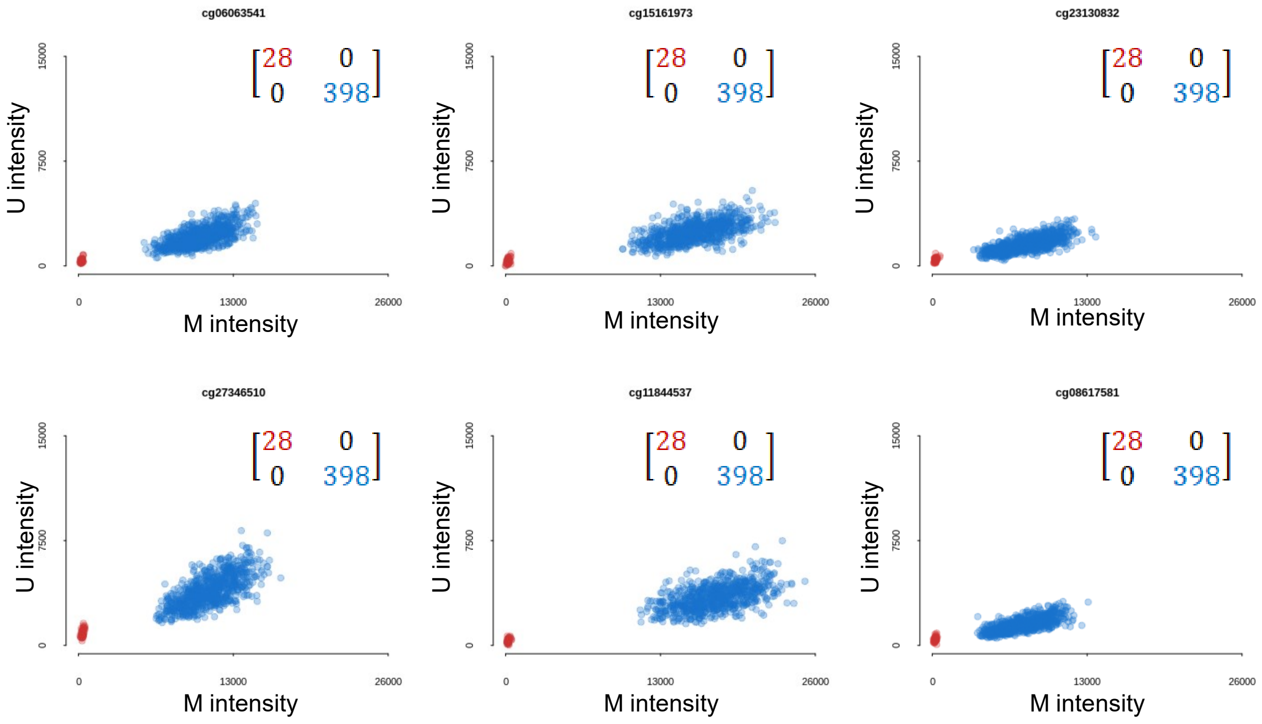

B

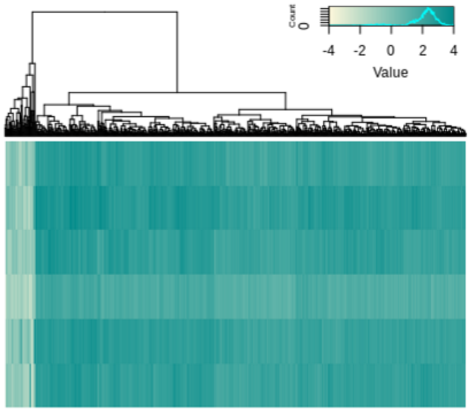

CG_ID	position
cg06063541	chr10:132909113
cg15161973	chr10:132909153
cg23130832	chr10:132909289
cg27346510	chr10:132911037
cg11844537	chr10:132911103
cg08617581	chr10:132911152

C

gssvL15561

Study	freq (ins)	freq (del)
DGV (Global)	0.633	0.367
E-risk n = 426	0.734	0.256

Figure S12. An example of an unannotated large indel that encompasses six contiguous 450K probes on chromosome 10. (A) U/M plots of all six probes. (B) Heatmap of M-values. (C) Observed and previously reported allelic frequencies of the variant. The variant gssvL15561 is reported as a copy number variation (CNV) of 6442 bp in the Database of Genomic Variants (which includes other ancestries apart from EUR). The observed differences in MAF estimation can be explained by population frequency differences.

A

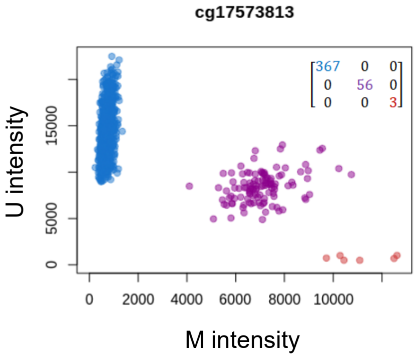

Type II, strand (+)

```
5'-GCCTA[C/G/T G]CCGGG-3'  (gDNA)
5'-GTTTA[Y/G/T G]TYGGG-3'  (BC gDNA)
             C ARCCC——— (probe, II)
```

rs9880846

Study	freq(C)	freq(T)	freq(G)
EUR, 1000 genomes n = 1006	0.797	0.147	0.056
E-risk n = 426	0.927		0.073

B

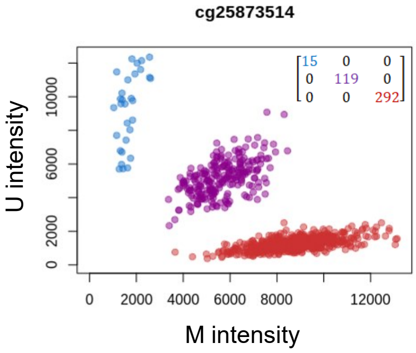

Type II, strand (+)

```
  5'-AGCTC[C G/C/A]GGGGG-3'  (gDNA)
  3'-TCGAG[G C/G/T]CCCCC-5'  (cDNA)
  3'-TTGAG[G Y/G/T]TTTTT-5'  (BC cDNA)
5'———AACTC[C]                (probe, II)
```

rs9915213

Study	freq(G)	freq(C)	freq(A)
EUR, 1000 genomes n = 1006	0.586	0.285	0.129
E-risk n = 426	0.825		0.175

Figure S13. Examples of triallelic CpG-SNPs. (A) cg27346510 and (B) cg25873514.

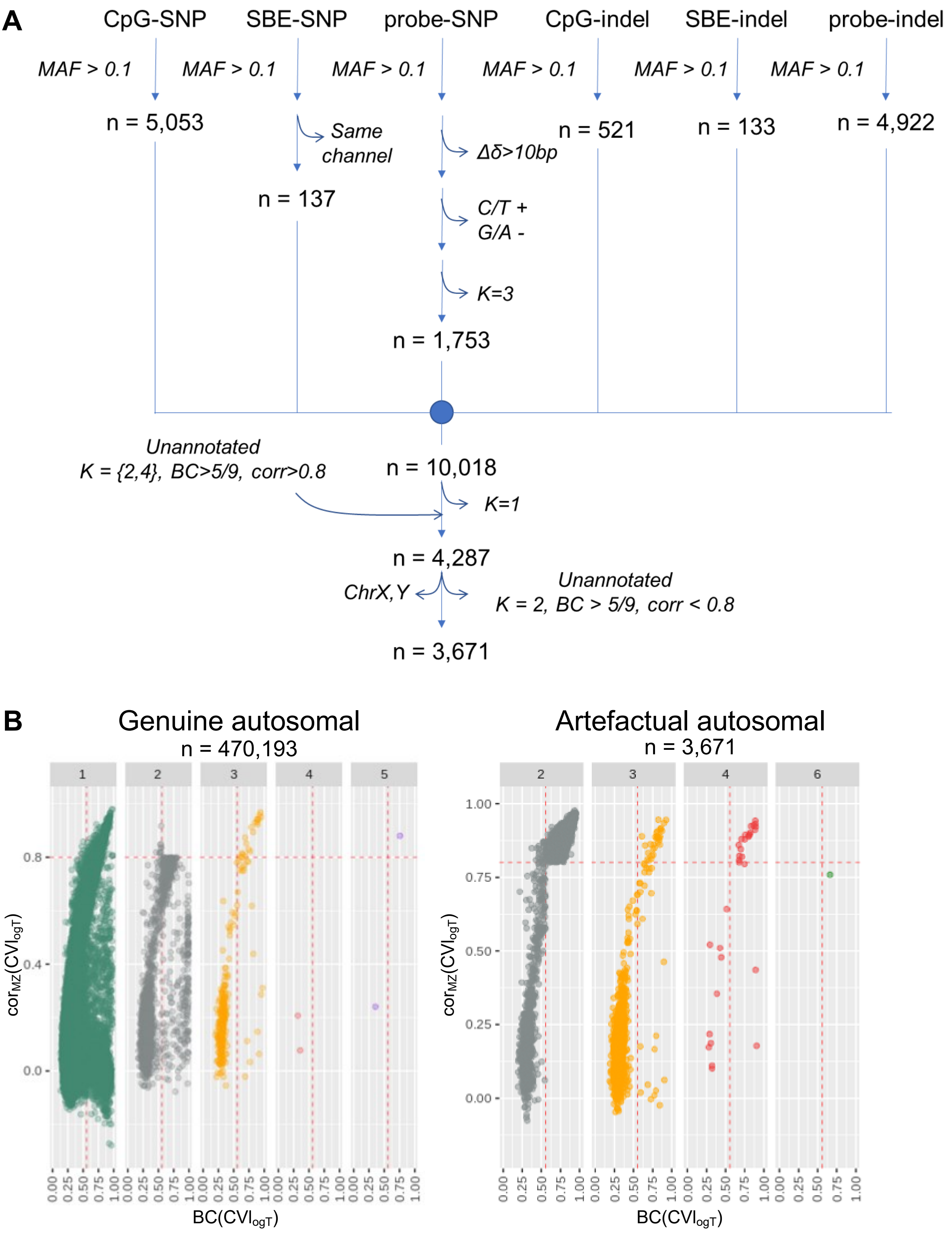

Figure S14. Set of predicted autosomal artefactual probes based on data-driven and *in silico* information. (A) Pipeline employed to obtain a list of autosomal artefactual probes. (B) Visualizations of the resulting sets (genuine-autosomal and artefactual-autosomal probes). Please note that allosomal probes (n = 11,648) are not included in this visualization.

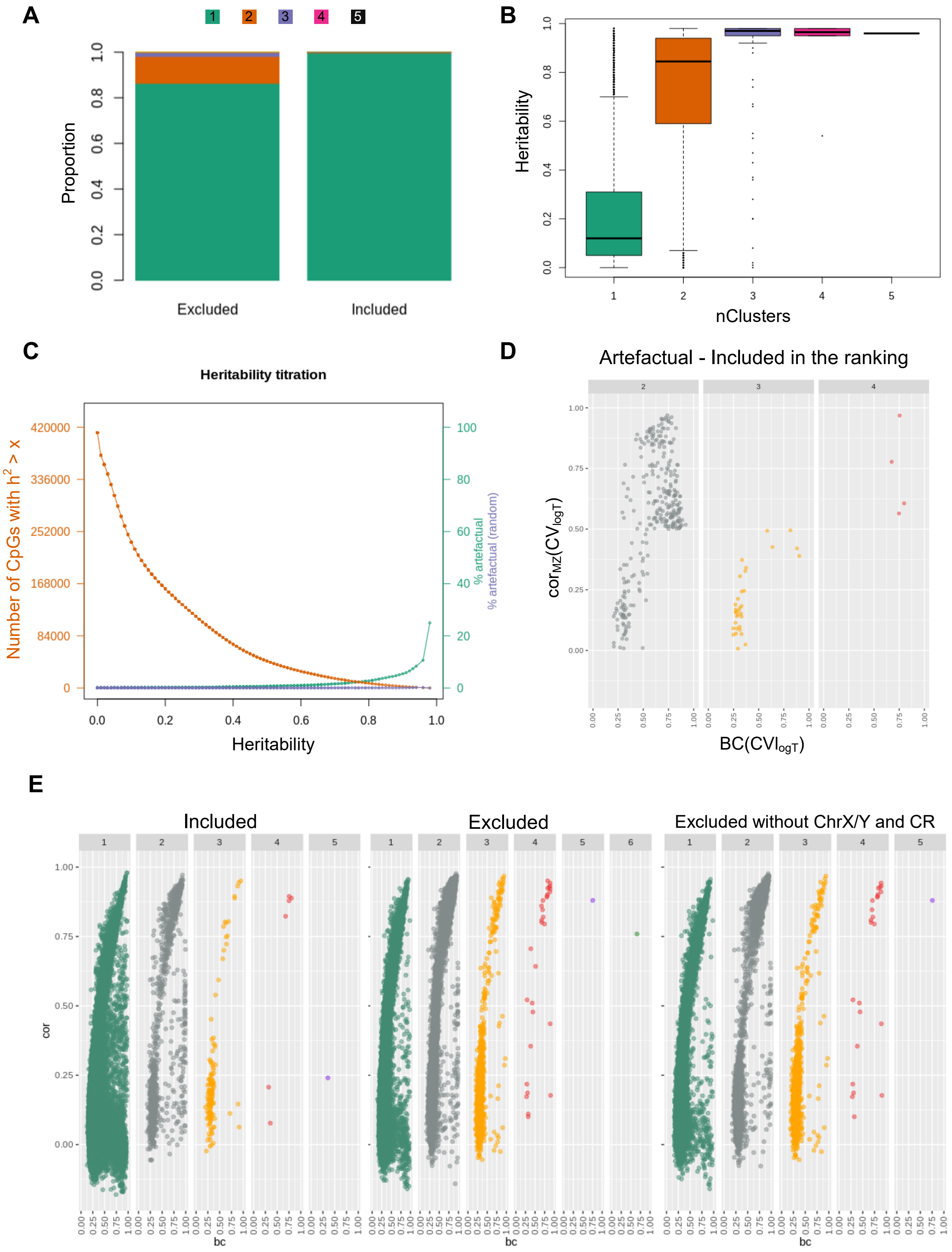

Figure S15. Quantification of the genetic artefact bleed-through in the heritability ranking of van Dongen *et al* (2016). (A) Distribution of nCluster called by K-caller in the excluded and remaining probes. (B) Correlation between heritability and nCluster (fueled by genetic artefacts and genuine meQTLs). (C) Heritability titration and quantification of % probes belonging to the artefactual set (green) and random set of probes (purple). The remaining number of CpGs is plotted in orange. (D) Members of the heritability ranking included in the artefactual set. (E) Visualization of the different sets (included in the ranking, excluded from the ranking, excluded from ranking but removing allosomal and cross-reactive (CR) probes)

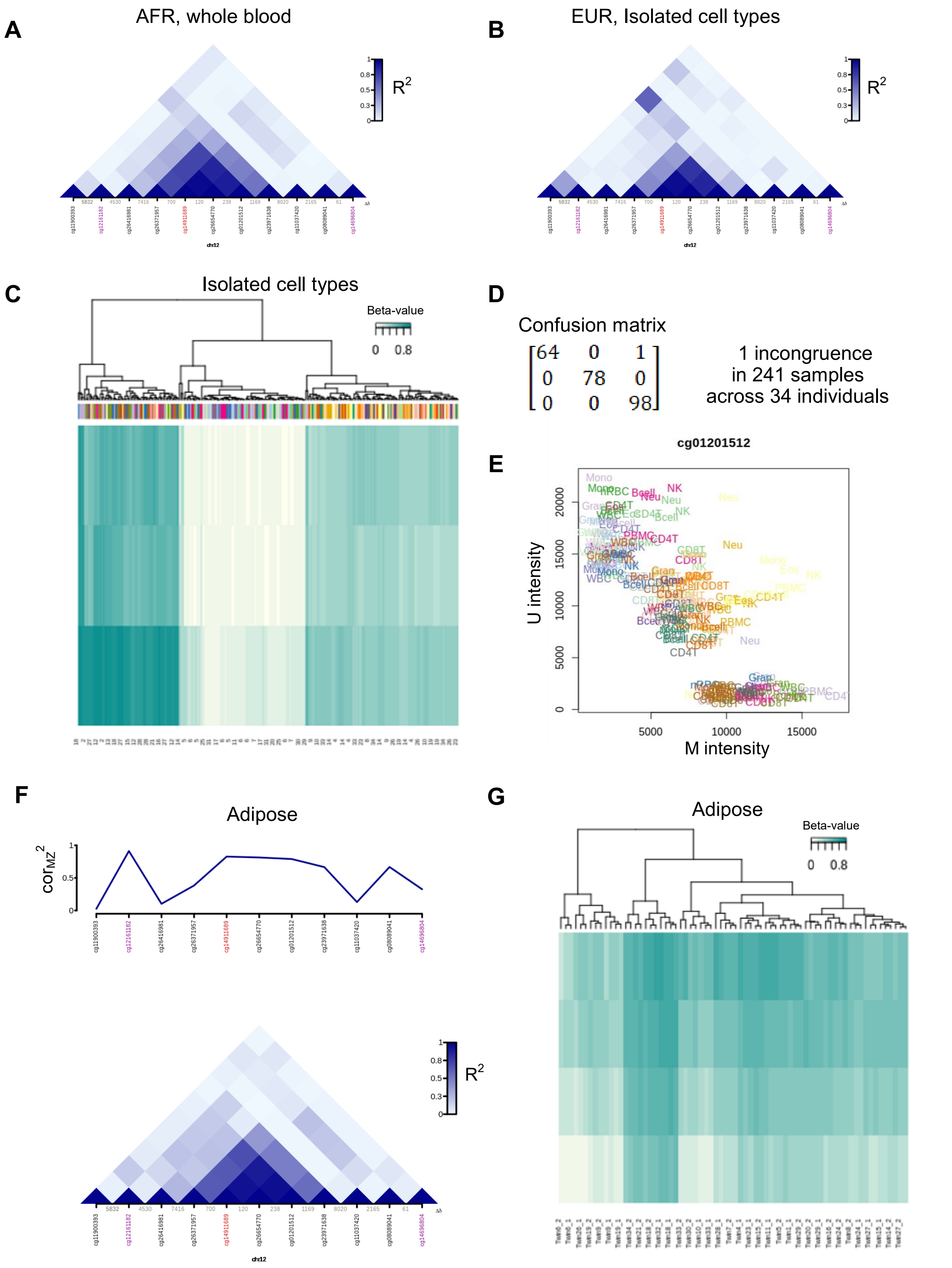

Figure S16. Validation of the NINJ2 meQTL across populations and tissues. (A) Co-methylation in blood of AFR ancestry and (B) in isolated blood cell types of EUR ancestry. (C) Heatmap of beta-values in isolated cell types. Each column colour represents a different cell type. (D) Quantification of methylation cluster incongruencies within references of the same individual. (E) Example of methylation patterns in one of the CpGs in the meQTL. (F) Co-methylation in adipose tissue of MZ twins. (G) Heatmap of methylation ratio in adipose tissue of MZ twins.

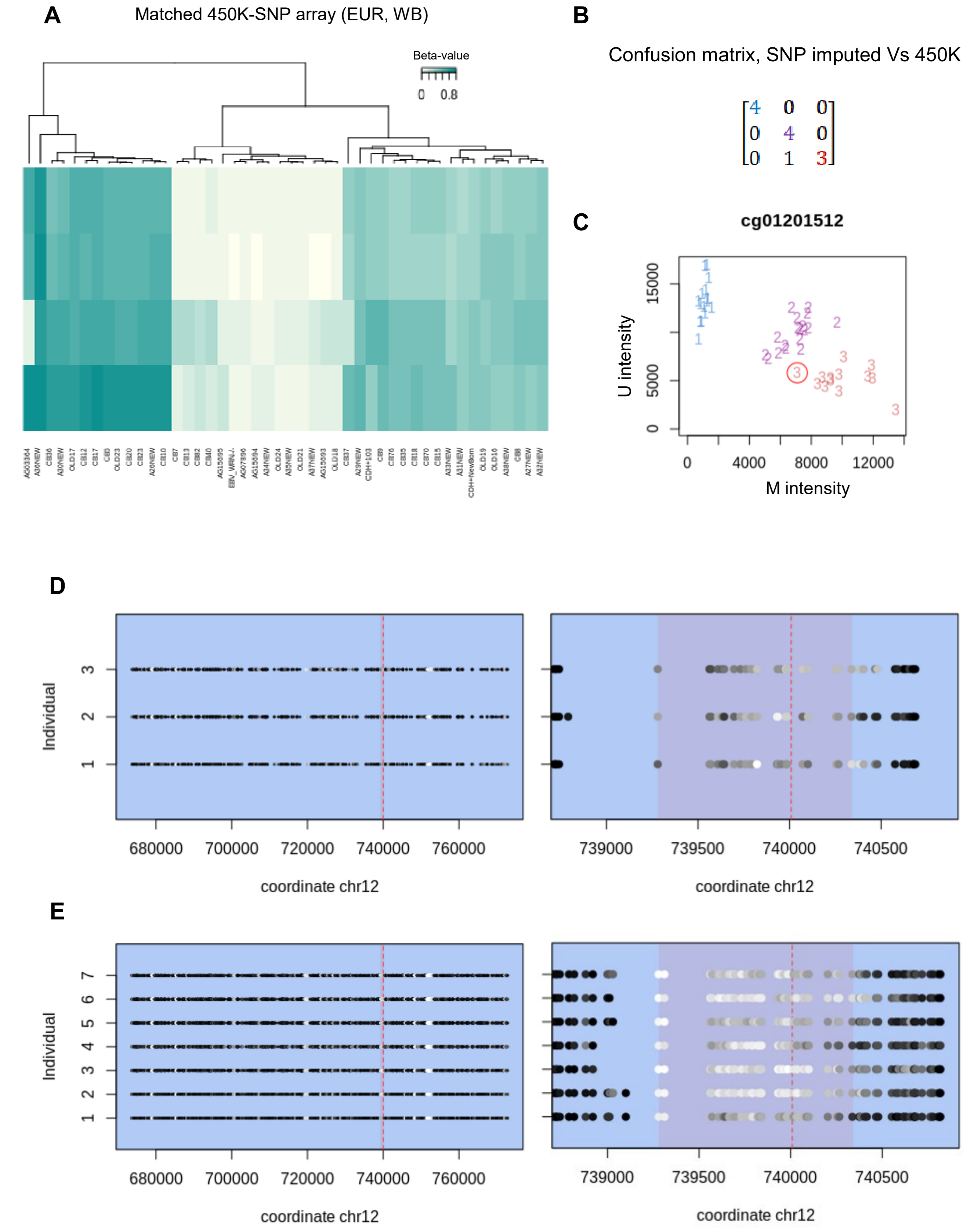

Figure S17. Analysis of the NINJ2-meQTL on matched 450K-SNP array and WGBS datasets. (A) Beta value heatmap of 450K samples. (B) Comparison between imputed genotype on the SNP array data and predicted genotype on the 450K platform. (C) An example CpG where the misclassification is highlighted. (D) Three matching WGBS samples from heterozygous individuals included in the 450K/SNP array datasets (newborn, middle-aged, 103 year old individual). The coverage seems low in the region. (E) Additional 7 WGBS samples from another study belonging to B-lymphocytes from healthy patients.

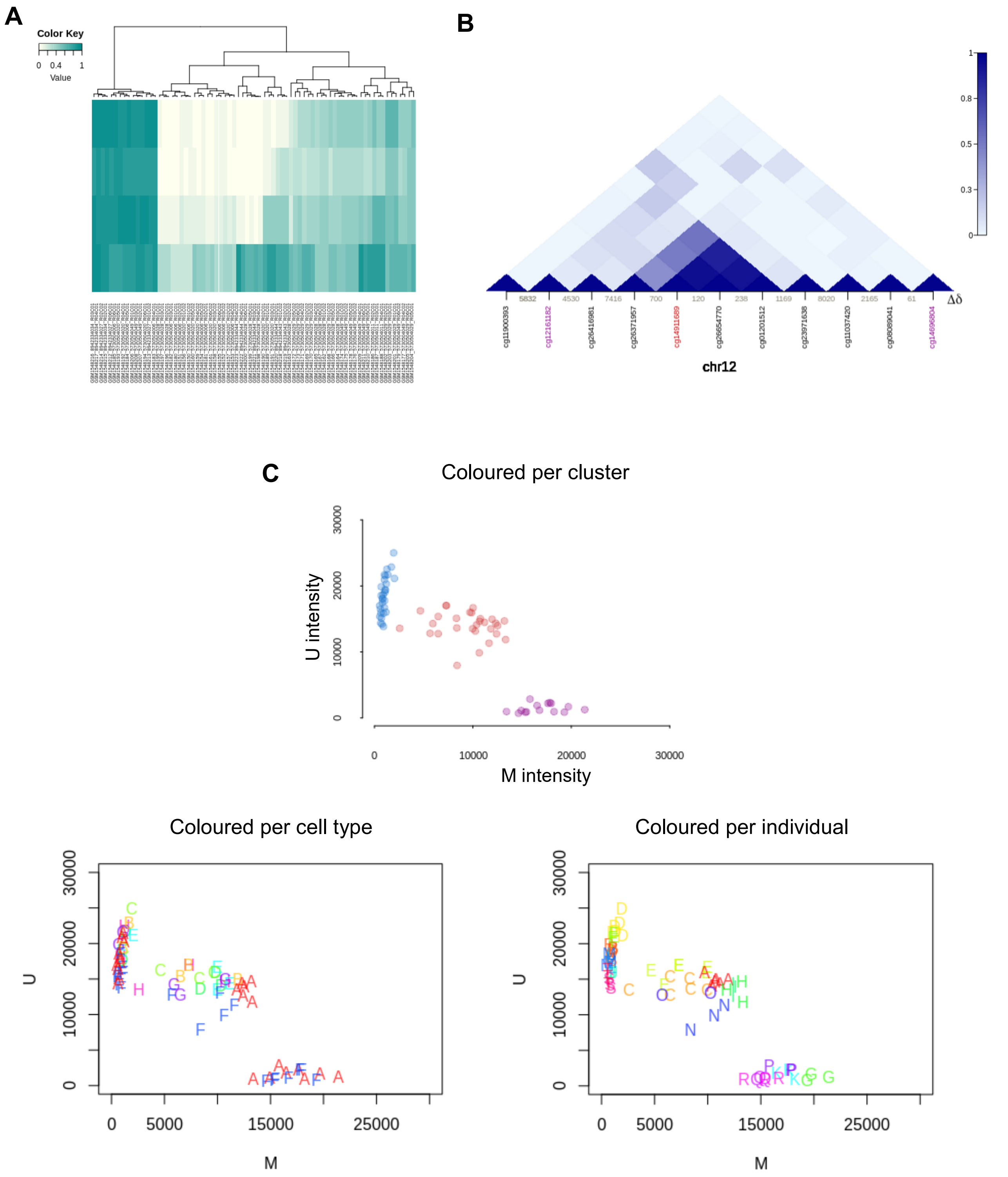

Figure S18. NINJ2 meQTL in early hematopoietic blood progenitors. (A) Methylation heatmap. (B) Co-methylation plot. (C) U/M plot of cg14911689 (within the meQTL) colour per cluster, per individuals (A-T, referring to individuals 1 to 20) and per cell type (A: blast cells; B: Common myeloid progenitor; C: Granulocyte-macrophage progenitor; D: Hematopoietic stem cells; E: Late-Multipotent progenitor cells; F: leukemia stem cells; G: Megakaryocyte-erythroid progenitor; H:MPP (Multipotent progenitor cells).

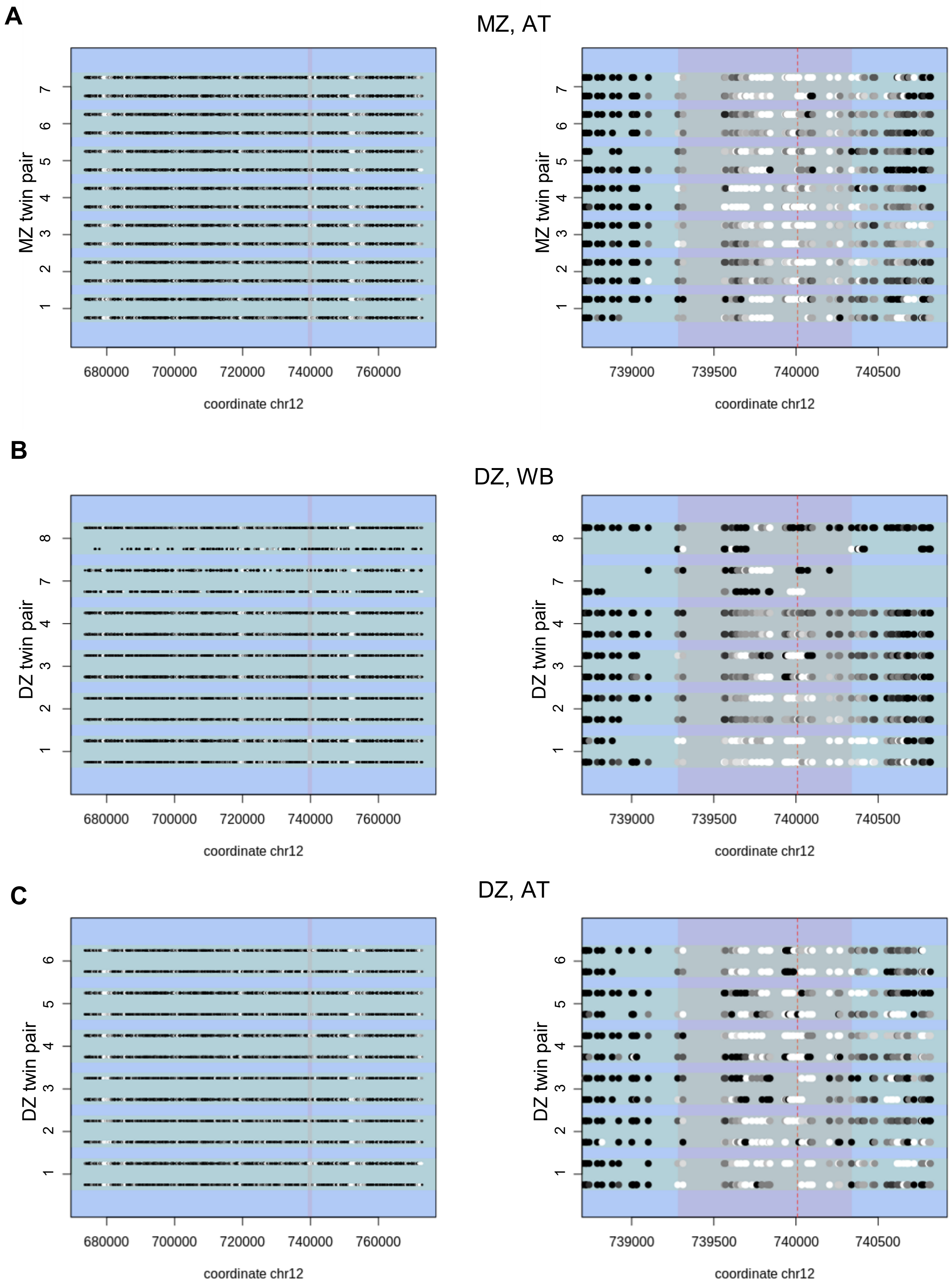

Figure S19. NINJ2 meQTL methylation in (A) Adipose tissue (AT) of monozygotic (MZ) twins, (B) whole blood (WB) of dizygotic twins (DZ) and (C) AT of DZ twins. Twin pair numbering was kept as in the original dataset (E-MTAB-3549).

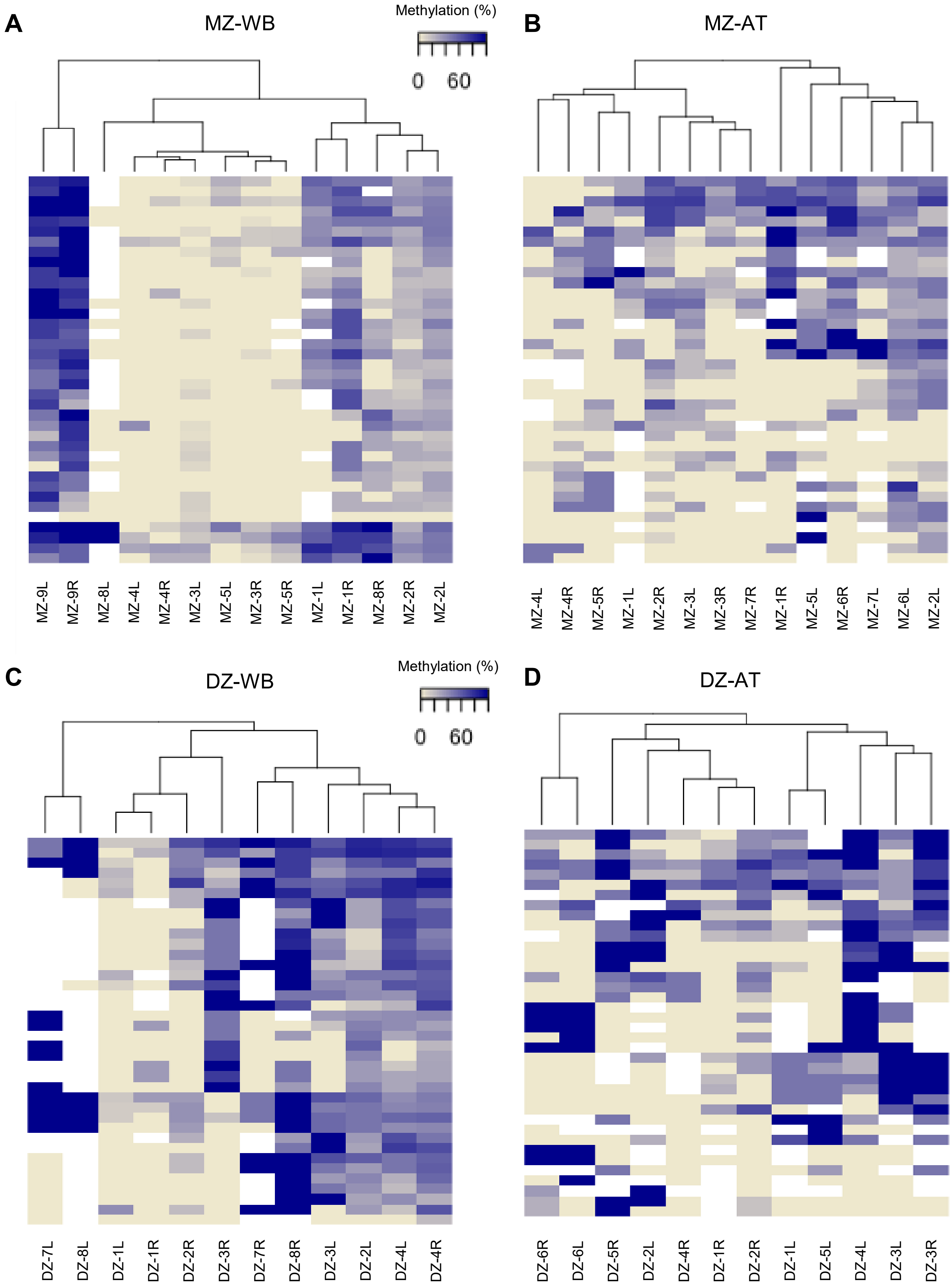

Figure S20. Heatmap of NINJ2 meQTL methylation in (A) Whole blood (WB) of monozygotic (MZ) twins (B) Adipose tissue (AT) of MZ twins, (C) WB of dizygotic twins (DZ) and (D) AT of DZ twins. Twin pair numbering was kept as in the original dataset (E-MTAB-3549). L and R stand for left and right and is simply one way to refer to each twin within a twin pair.

A

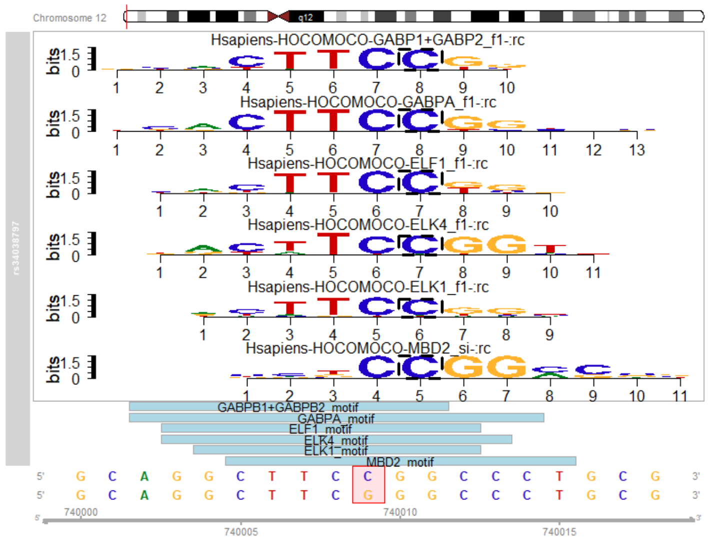

B

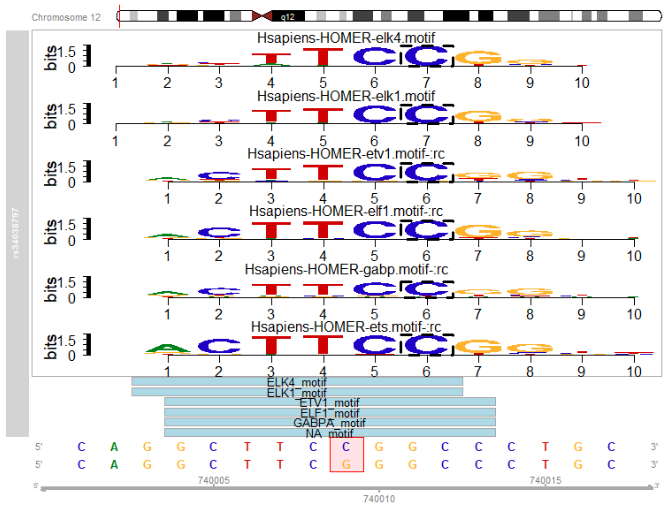

C

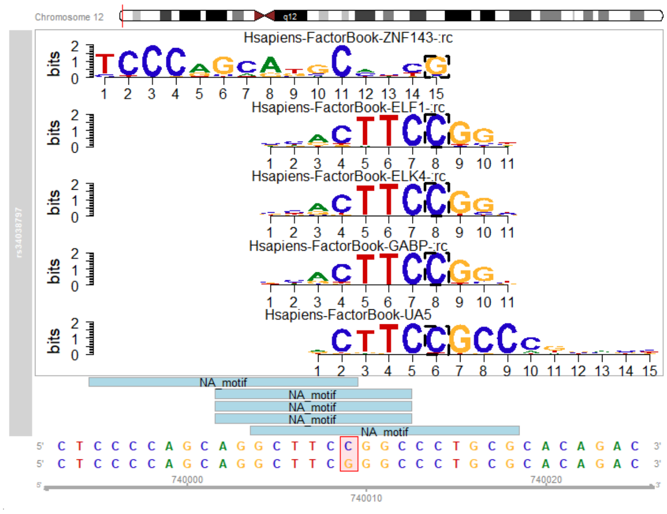

Figure S21. SNP-TFBS association. Output of motifbreakR analysis against (A) HOMOCO, (B) HOMER, and (C) FactorBook.

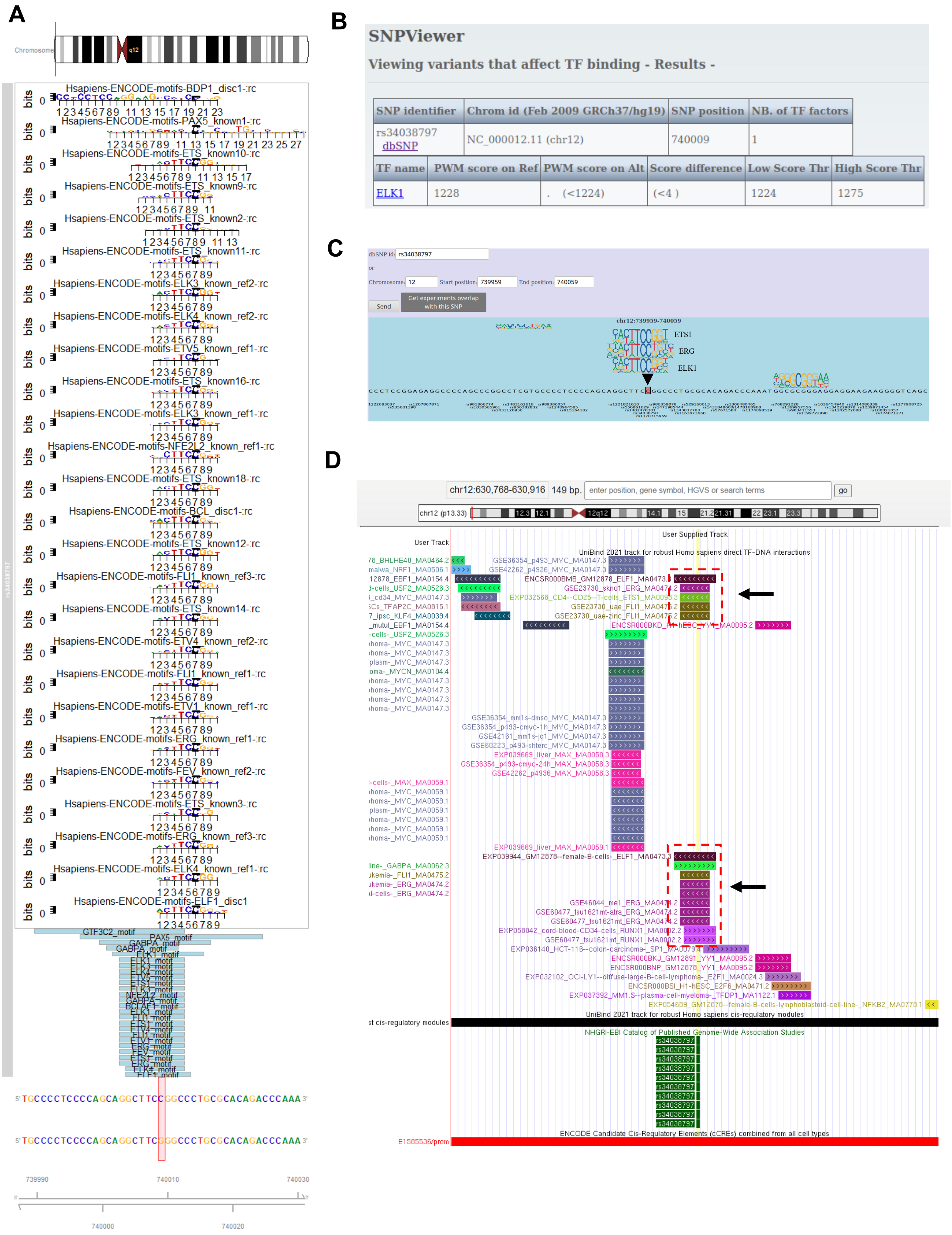

Figure S22. (A) Output of motifbreakR analysis against ENCODE. (B) Output of SNP2TFBS, SNPViewer (SIB) web tool analysis. (C) Output of ChIPSummitDB web tools analysis. (D) Unibind UCSC track focused on rs34038797, highlighted collocating TFBS detected via ChIP-seq in several different tissues (see legend on the left).

A

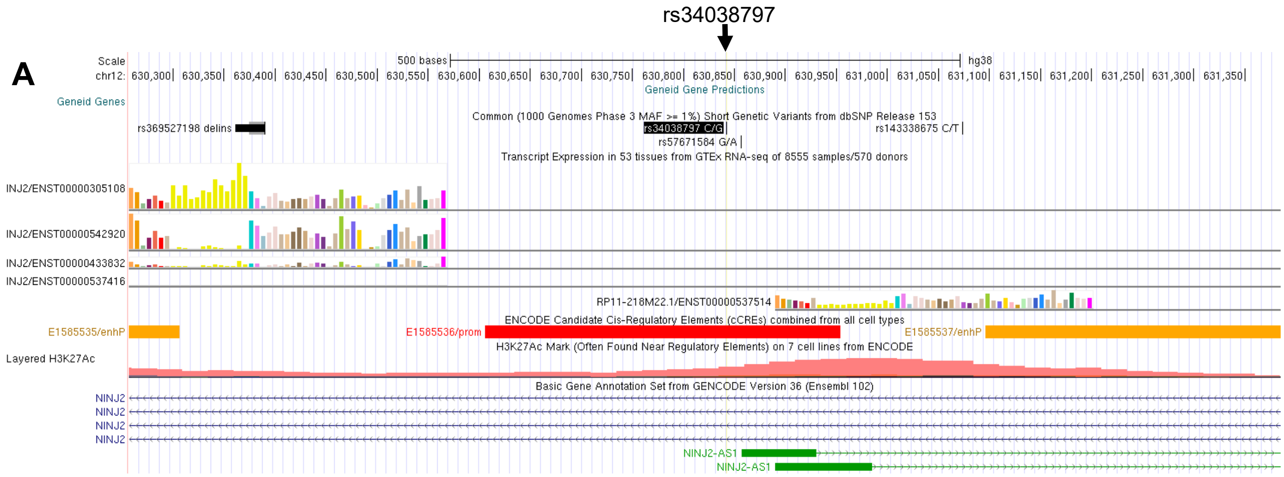

B

	TFBS	Methylation<br>mQTL	H3K27ac<br>haQTL	RNA Expression<br>eQTL	Chromatin Accessibility<br>caQTL	Transcript Usage<br>tuQTL	Phenotype
rs34038797-C	Active	Unmethylated	Acetylated	NINJ2-AS1 Expressed	Accessible	NINJ2-205 (CDS:106 aa)	Increase in Platelet/ Lymphocite/ Monocyte count
rs34038797-G	Inactive	Methylated	Non-acetylated	NINJ2-AS1 Not expressed	Not accessible	NINJ2-202 (CDS:135 aa)	Decrease in Platelet/ Lymphocite/ Monocyte count

C

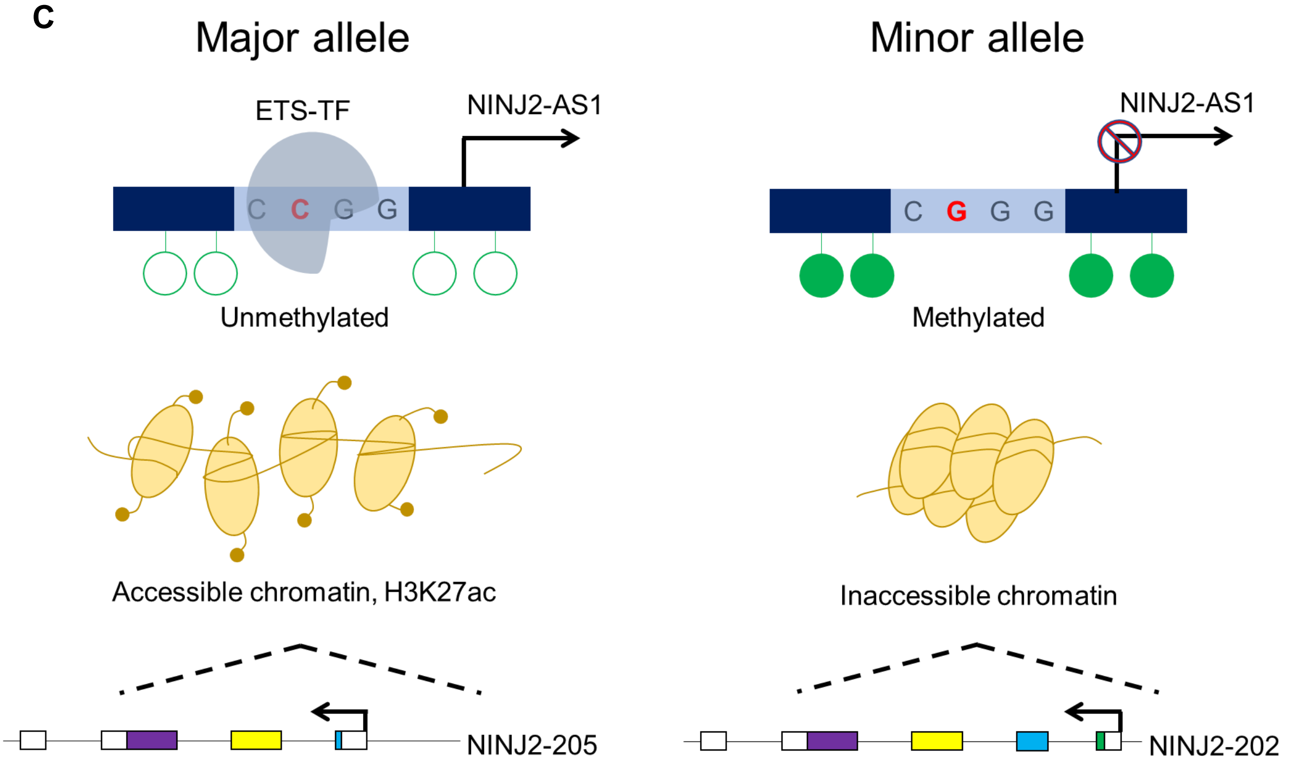

Figure S23. SNP-TFBS association and cross-omics approach. (A) Output of the UCSC browser at the region highlighting the position of the putative *cis*-causal variant, collocating with the promoter of NINJ2-AS1. (B) Summary of cross-omics annotation of the SNP rs34038797. (C) Graphical epigenomic model on this locus.

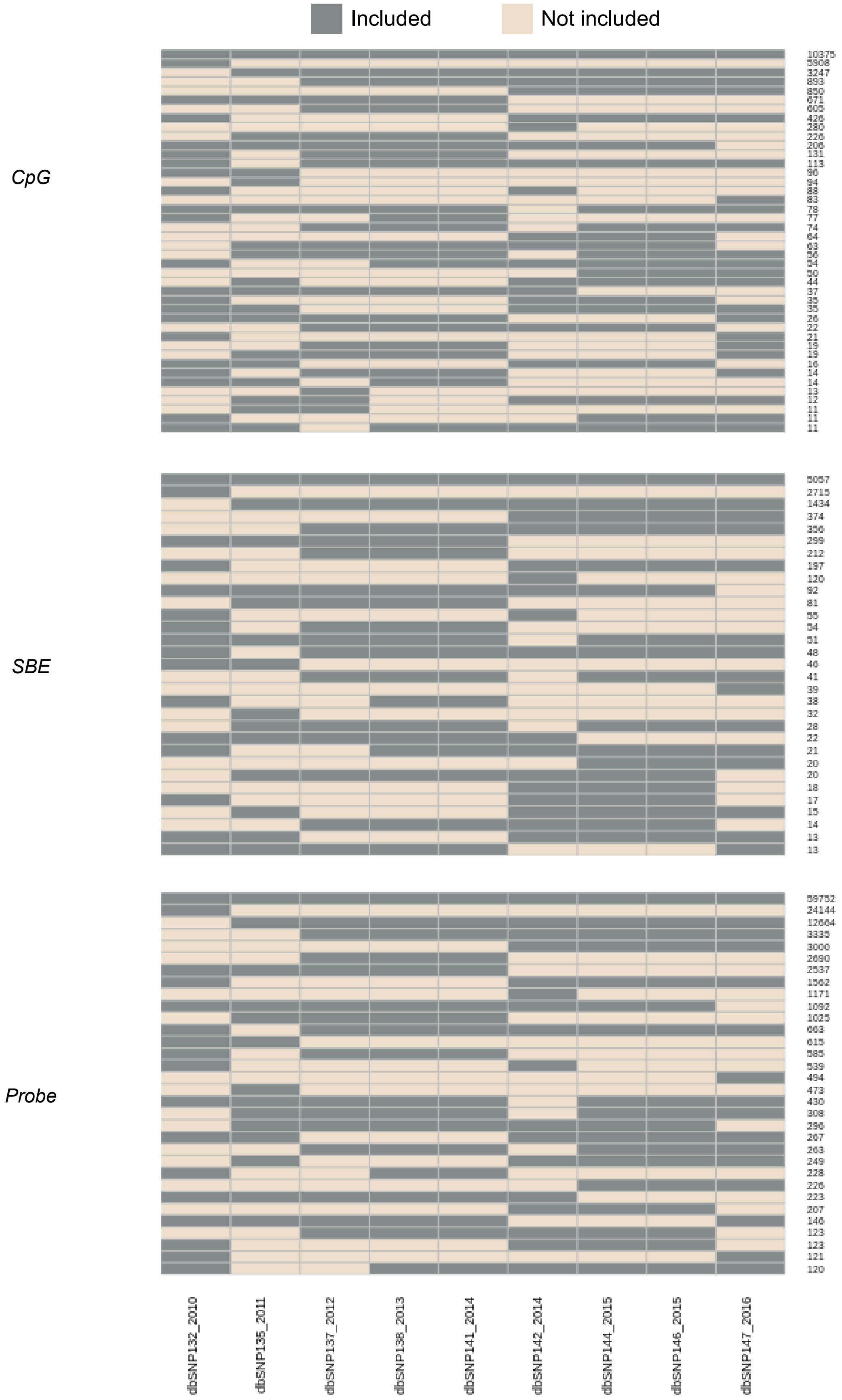

Figure S24. Variation probes associated to SNPs across several versions of dbSNP. The SNPs.CommonSingle files from the IlluminaHumanMethylation450kanno.ilmn12.hg19 offer an opportunity to examine the pattern of variation in dbSNP across the years. Either (A) CpG, (B) SBE and (C) Probe SNPs patterns across versions of dbSNPs are examined. All patterns of inclusion are scanned but only the top hits are displayed. The number of instances of each row behavior is displayed as a number on the far right row display.

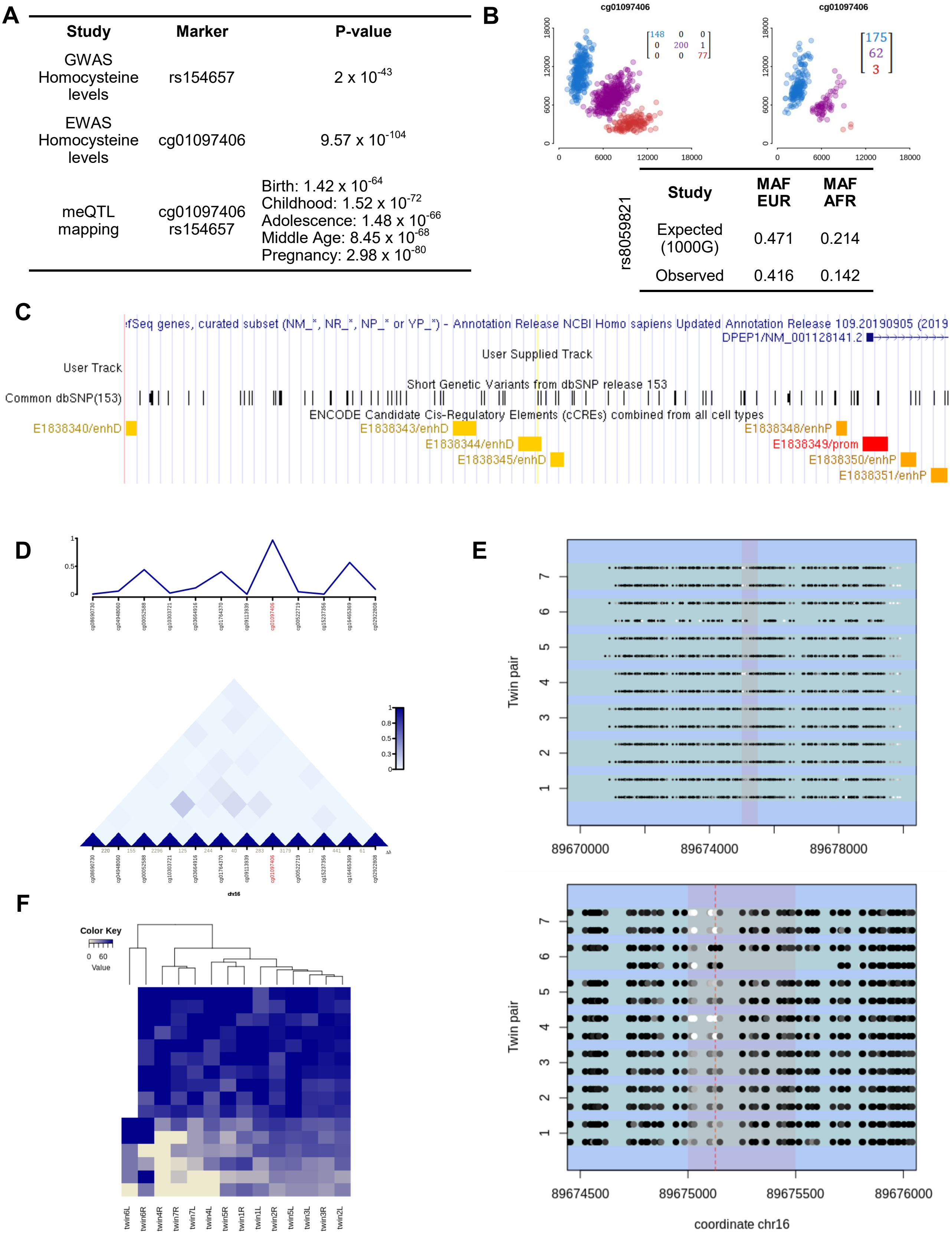

Figure S25. Putative example of a proxy-GWAS based on DNA methylation data. (A) Summary statistics for EWAS, GWAS and meQTL mapping. (B) MAF estimation and the identification of a putative *cis*-causal variant, rs8059821, performed like on our validation of the NINJ2 meQTL. (C) UCSC browser snapshot centered at rs8059821. (D) Lack of co-methylation of cg01097406. (E) WGBS on MZ twins centered on cg01097406-targeted CpG displaying inter-individual variation on a very short region that includes the putative *cis*-causal variant. (F) WGBS methylation heatmap across MZ twins in the region highlighted with a red window in E.

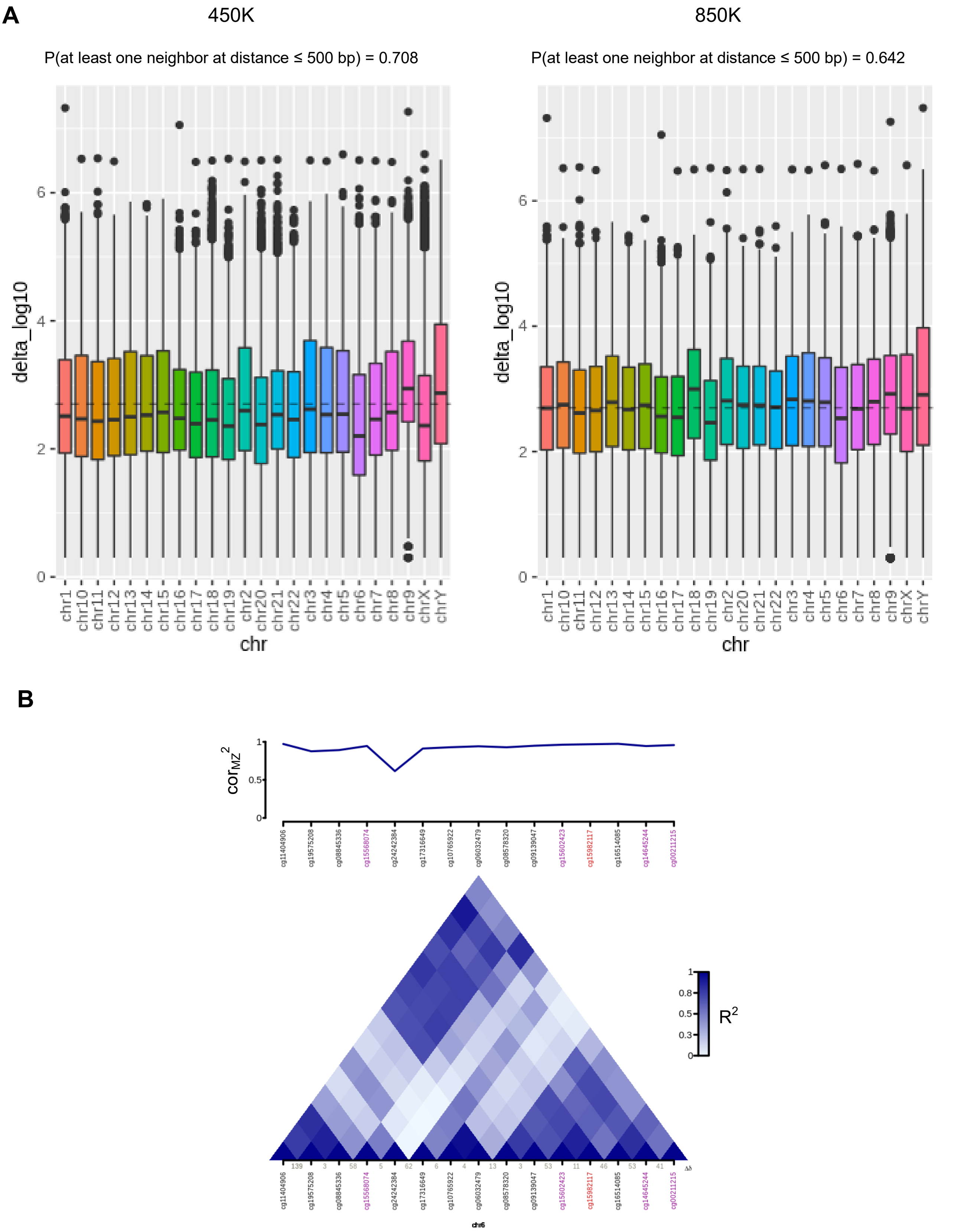

Figure S26. Assessing the limitations of the co-methylation strategy. (A) Distribution of distance of CpG neighbours in the 450K and the 850K DNA methylation microarrays. The line indicates $\log_{10}(500)$, as a threshold for observing co-methylation. (B) Co-methylation at the HLA-DRB1 locus due to networks of linkage disequilibrium between genetic artefacts.

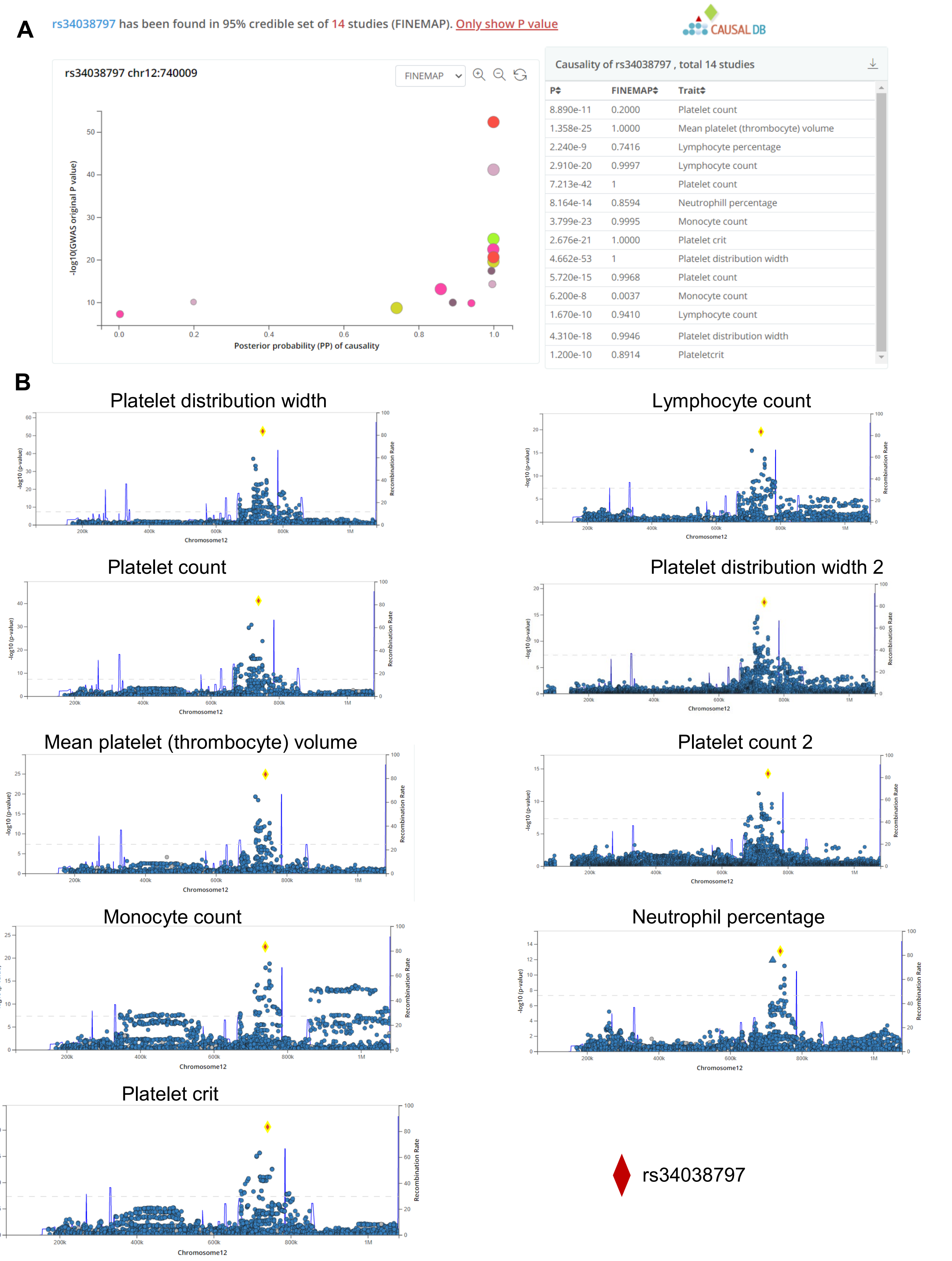

Figure S27. CausalDB Manhattan focusing on the NINJ2 region. (A) CausalDB (B) Highlighted variant corresponds to rs34038797. Only traits with p-val < 10^{-13} are displayed.

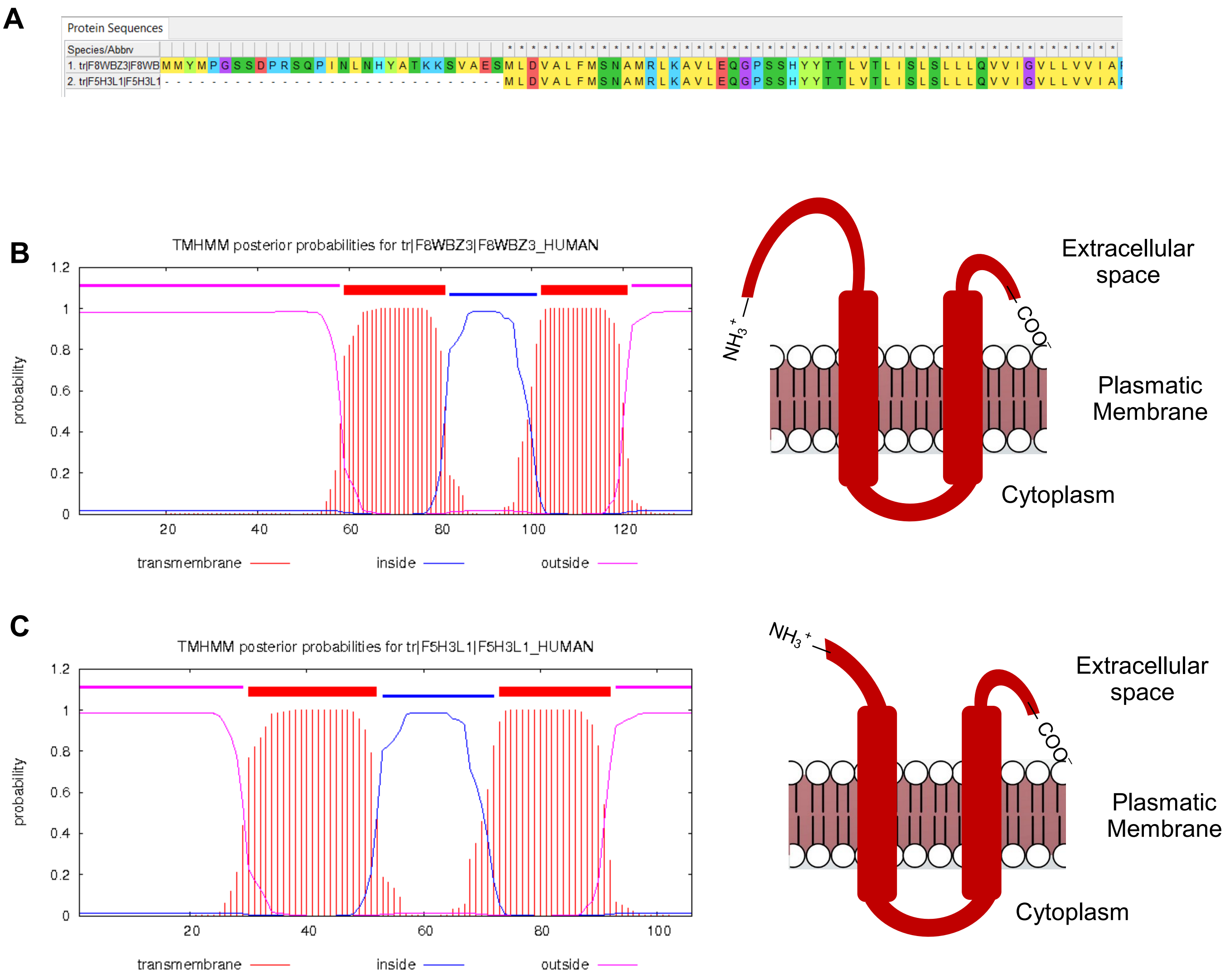

Figure S28. TMHMM output on the short and long version of NINJ2. (A) Alignment of the N-terminal in the short and long versions of NINJ2 protein (MEGAX). Output of TMHMM for the (B) long and (C) short version, with a simple schematics.
