# Supplementary material for: Revisiting genetic artifacts on DNA methylation microarrays exposes novel biological implications
Source: Genome Biol. 2021 Sep 21;22:274. doi: 10.1186/s13059-021-02484-y (PMC8454075; doi:10.1186/s13059-021-02484-y)
Supplement: Supplementary file 2 — Additional file 2. Supplementary methods. [file 13059_2021_2484_MOESM2_ESM.pdf]

Supplementary Methods

Revisiting genetic artefacts on DNA methylation microarrays exposes novel biological implications

Benjamin Planterose Jiménez[1], Manfred Kayser[1], Athina Vidaki[1,*]

[1] Erasmus MC, University Medical Center Rotterdam, Department of Genetic Identification, Rotterdam, the Netherlands

[*] Correspondence: Dr. Athina Vidaki, E-mail: a.vidaki@erasmusmc.nl

Table of contents

1. Technical details on the Illumina 450K DNA methylation assay.. 3
2. U/M plot: slopes and methylation ratios.. 5
3. Detection p-values and the definition of CV_{logT}, $BC(CV_{logT})$ and $cor_{MZ}(CV_{logT})$ 6
4. K-calling 9
5. Scoring benchmark performance 10
6. Annotation of 450K/850K probes dbSNP151...11
7. Variant calling and minor allele frequency estimation from methylation data 13
8. Identification of representative examples 17
9. SNP imputation 19
10. Guidelines on how to repurpose UMtools..19

1. Technical details on the Illumina 450K DNA methylation assay

The main DNA methylation microarray platforms have been developed by Illumina; in chronological order of development: Golden Gate Assay for methylation, Infinium HumanMethylation27 BeadChip (27K), Infinium HumanMethylation450 BeadChip (450K) and Infinium MethylationEPIC array BeadChip (850K). Though EPIC is the newest platform, 450K is still the platform with most data available. Also, there is no technological leap involved between 450K and 850K, simply addition-elimination of markers.

A) BeadChip technology

Initially developed for genotyping arrays, a BeadChip microarray consists of a silicon substrate with regularly interspaced micro-wells. Silicon micro-beads randomly self-assemble in the wells. Each bead is covered by hundreds of thousands of copies of the same 50-nucleotide long probe. During the manufacture, beads are pooled in equal ratio and then deposited on the array. As a result, the exact number of copies of each *beadType* is not controlled (varies from chip to chip). To assign the correspondence between microwells and bead types, decoding is required. This is done during manufacture via consecutive hybridizations with other sets of probes that target the address, a 23 nucleotide-long oligonucleotide handle (address) that links the bead to the probe in the form of a DMAP file [5].

B) Methylation assay protocol on the chip

The experimental protocol can be broken down to genomic DNA (gDNA) extraction, gDNA bisulfite conversion (unmethylated cytosines are converted uracils), whole-genome amplification, enzymatic DNA fragmentation, hybridization to the microarray, washing and staining via single-base extension (SBE). This last step is initialized via incubation with DNA polymerase and labelled dideoxynucleotides-triphosphate (ddNTPs): ddATP and ddTTP labelled with biotin, ddCTP and ddGTP labelled with dinitrophenol (DNP); upon SBE, elongation interrupts due to the dideoxy nature of the incorporated nucleotide. Staining is finally carried out by incubating with red-fluorescing Cy5-labelled anti-DNP (targeting ddA/T) and green-fluorescing Cy3-labelled streptavidin (targeting ddC/G). Fluorescence is acquired in two separate channels (Green and Red) with iScan/HiScan confocal laser microarray scanner and stored in two files (one per fluorescence channel). The .IDAT (Intensity Data) extension is Illumina's proprietary format for storage of microarray scanners' raw fluorescence output and is encrypted and non-human readable. After decrypting the two output files, it is possible to extract the number of beads per beadType (nBeads), the mean and SD in fluorescence intensity of each beadType across bead replicates in the green and the red channel (G, R, G_SD and R_SD) plus technical metadata [5, 54].

C) Infinium assays

Three distinct assays coexist simultaneously on the same chip. Infinium type II (TII) probes target both epialleles with one oligonucleotide probe; the probe outstretches its 3'-end until one nucleotide before the targeted cytosine and as a result, SBE occurs at the target cytosine position and is informative in both fluorescence channels: green and red channel correspond to methylated (M) and unmethylated (U) epialleles, respectively. Infinium type I green (TI^{Green}) and Infinium type I red (TI^{Red}) target each epiallele with two different oligonucleotides. The 3'-end of $T\text{-}I^{G}$ and $T\text{-}I^{R}$ probes reaches the targeted cytosine and as a result, SBE occurs one nucleotide after the targeted cytosine. In this case, SBE for TI^{G} or TI^{R} is informative either on the green or the red channel, respectively. Also note that, although TI^{R} and TI^{G} are informative on one sole channel, the fluorescence intensities of both channels can be found in the IDAT files. Finally, it is very important to note that Illumina probes target cytosines either at the plus or the minus strand depending on the site under consideration.

C) Methylation quantification per probe type

For each probe type, the methylation ratio (β) is computed as:

$$\beta = \frac{M}{M + U + \alpha}$$

where M is the mean fluorescence intensity corresponding to the methylated epiallele, U is the mean fluorescence intensity corresponding to the unmethylated epiallele and α is a small constant for numerical stability. But the fluorescence intensities M and U depend on the bead type in question:

$$\beta_{TII} = \frac{\mathbf{G_M}}{\mathbf{G_M} + \mathbf{R_U} + \alpha};\ \beta_{TI^{Green}} = \frac{\mathbf{G_M}}{\mathbf{G_M} + \mathbf{G_U} + \alpha};\ \beta_{TI^{Red}} = \frac{\mathbf{R_M}}{\mathbf{R_M} + \mathbf{R_U} + \alpha}$$

where G is the mean green fluorescence intensity across beads, where R is the red mean fluorescence intensity across beads. Also note that for TII probes, the number of beads for each channel is the same. This is not necessarily the case for TI^{R} and TI^{G}.

D) Probes included in the 450K

In the 450K, the methylation status of a total of 485,512 predefined cytosines are interrogated by 622,399 beadTypes. This excess in beadTypes is due to the presence of control beadTypes and the existence of type-I probes that consume two beadTypes per cytosine assayed. The exact count of *BeadTypes* is the following:

- Type I (n = 135,476 x 2)
    - Type-I Green (n = 46,289 x 2)

    - Type-I Red (n = 89,187 x 2)
- Type-II (n = 350,036)
- Control probes (n = 848)
    - Staining (n = 4)
    - Extension (n = 4)
    - Hybridization (n = 3)
    - Target removal (n = 2)
    - Bisulfite conversion I and II (n = 12 and 4, respectively)
    - Specificity I and II (n = 12 and 3, respectively)
    - Non-polymorphic (n = 4)
    - Negative control (n = 613)
    - Restoration (n = 1)
    - Normalization (n = 186)
- SNP-targeting probes (n = 90)
    - SnpI (n = 25 x 2) – SNP genotyping via Infinium I assay
    - SnpII (n = 40) – SNP genotyping via Infinium II assay
- Orphan probes (n = 473) - placed on the array for an unknown purpose

2. U/M plot: slopes and methylation ratios

In the presence of batch effects, U/M fluorescence signals are spread in the U/M plane forming rod-like structures. Assuming i) an absence of background fluorescence and ii) a linear dependence between U and M for each rod, we can write the slope, S, as:

$$S = \frac{\partial U}{\partial M} \overset{ii)}{\cong} \frac{\Delta U}{\Delta M} = \frac{U - U(0)}{M - M(0)} \overset{i)}{\cong} \frac{U}{M}$$

The slope S is related to the beta-value or methylation ratio as:

$$\beta = \frac{M}{U + M} = \frac{1}{\frac{U}{M} + 1} = \frac{1}{S + 1}$$

And as a result, we intuitively obtain the following results:

$$\lim_{S \to +\infty} \beta(S) = 0;\ \beta(S = 0) = 1;\ \beta(S = 1) = 1/2$$

Defining the angle of the slope, α, the beta-value is related to alpha as:

$$\alpha = \operatorname{atan}\left(\frac{U}{M}\right) \to \beta = \frac{1}{\tan(\alpha) + 1}$$

Counterintuitively, although 0, 50 and 100% methylation levels coincide with 0, π/4 and π/2 radians, the beta-value does not change linearly with α:

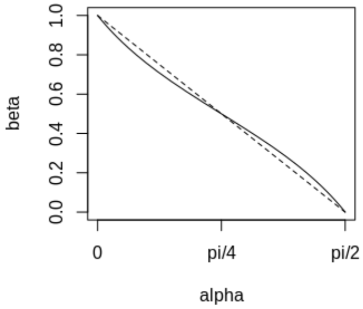

$$\frac{\partial\beta}{\partial\alpha} = \frac{-1/\cos^2(\alpha)}{\tan^2(\alpha) + 1 + 2\cdot\tan(\alpha)} = \frac{-1}{\sin^2(\alpha) + \cos^2(\alpha) + 2\cdot\sin(\alpha)\cdot\cos(\alpha)}$$
$$= \frac{-1}{1 + 2\cdot\sin(2\alpha)} \neq 0$$

3. Detection p-values and the definition of CV_{logT}, $BC(CV_{logT})$ and $cor_{MZ}(CV_{logT})$

In any fluorescence-based assay, it is very important to test whether a given fluorescence signal is simply background fluorescence. In the case of a DNA methylation ratio, we can express it as the following hypothesis contrast:

$$\begin{cases} H_0: U + M = \mu_{Bg} \\ H_1: U + M \neq \mu_{Bg} \end{cases}$$

As standard practice in epigenomics, we make the following strong assumptions:

$$(\mathrm{U} + \mathrm{M})_{H_0} \sim \mathrm{N}(\mu_{Bg}, \sigma_{Bg})$$

As a result, detection p-values can be estimated as:

$$\mathrm{detP_{ij}} = 1 - \phi\left(\frac{\mathrm{U}_{ij} + \mathrm{M}_{ij} - \hat{\mu}_{Bg}}{\hat{\sigma}_{Bg}}\right)$$

Where ϕ is the standard normal cumulative density function. To estimate background fluorescence mean and standard deviation, we expand per probe type as:

$$\mu_{TII} = Bg_G + Bg_R;\ \ \sigma_{TII} = \sqrt{\sigma_G^2 + \sigma_R^2 + 2 \cdot Cov(G,R)} \leq \sigma_G + \sigma_R$$

$$\mu_{TI_R} = 2 \cdot Bg_R;\ \ \sigma_{TI_R} = \sqrt{\sigma_R^2 + \sigma_R^2 + 2 \cdot Cov(G,R)} \leq 2 \cdot \sigma_R$$

$$\mu_{TI_G} = 2 \cdot Bg_G;\ \ \sigma_{TI_G} = \sqrt{\sigma_G^2 + \sigma_G^2 + 2 \cdot Cov(G,R)} \leq 2 \cdot \sigma_G$$

For the estimation of Bg_G and Bg_R, several approaches exist:

- minfi::detectionP: it uses negative control probes on each fluorescence channel to estimate the backgrounds.
- EWAStools::detection: it uses the unmethylated intensities for completely methylated probes and *vice versa* on each channel to model the backgrounds.
- sesame::pOOBAH: it uses out-of-band fluorescence (the red channel of TI_G or the green channel of TI_R) to estimate the backgrounds.

In any of the cases, using the ENmix::QCinfo criterion as an example, if for a given CpG 5 % of the samples show detection p-values larger than 0.000001, then the CpG is excluded from analysis. Similarly, if 5 % of the CpGs in a given sample exceed a detection p-value of 0.000001, then the sample is excluded.

Instead of trying to decide what is signal and what is noise, another strategy could consist in quantifying the signal-to-noise ratio. For that, we can use SD_{red} and SD_{green} (standard deviation across beads), stored on every IDAT file but never used or cited in the literature, probably due to the scarcity of information concerning Illumina's proprietary .IDAT format and the current preference in large cohorts to employ pre-normalized data.

Also, to account with the huge diversity in fluorescence dynamic ranges in the array, we will be log-transforming fluorescence intensities. We define the natural logarithm of the total signal of a given individual and a given CpG, logT, as:

$$\log(T) = \log\,(U + M)$$

We define the coefficient of variation of the logarithm of the total signal as:

$$CV_{logT} = \frac{\sigma_{logT}}{\mu_{logT}}$$

Where σ_{logT} is the standard deviation of logT across beads for a given CpG and individual.

To approximate CV_{logT}, it is necessary to make assumptions about the dependency relationships between U and M across beads:

$$\sigma_T^2 = \sigma_U^2 + \sigma_M^2 + 2 \cdot Cov(U,M)$$

This covariance term cannot be manually examined as only sample means and standard deviations across beads are stored in the .IDAT raw file. For type-I probes, it is expected to be independent (M and U probes lie in different beads) while for type-II probes, positive dependence is expected as probes lie on the same bead. Hence, $\rho \geq 0$:

$$\sigma_T \in \left[\sqrt{\sigma_U^2 + \sigma_M^2}, \qquad \sigma_U + \sigma_M\right]$$

We define CV_{logT} as the most pessimistic scenario, in which the maximum variance is obtained.

$$CV_{logT} \stackrel{\text{def}}{=} \max\left(CV_{logT} | U, M, \sigma_M, \sigma_U\right) \rightarrow \sigma_T = \sigma_U + \sigma_M$$

To estimate CV_{logT}, we also need to understand how the logarithm affects the mean and variance statistics. If T is a strictly positive random variable with mean μ_T and variance σ_T^2, we can estimate $\mu_{\ln(T)}$ and σ_{logT}^2 employing Taylor series expansion of the moments of a random variable:

$$E[g(X)] = g(\mu_X) + \frac{g''(\mu_X)}{2}\sigma_X^2 \quad (2^{nd}\text{ degree})$$

$$\mathrm{Var}\left(g(X)\right) = (g'(\mu_X))^2\sigma_X^2 \qquad (1^{st}\text{ degree})$$

Giving rise to the following expressions:

$$\mu_{logT} \approx \log(\mu_T) - \frac{\sigma_T^2}{2 \cdot \mu_T^2}; \qquad \sigma_{logT}^2 \approx \frac{\sigma_T^2}{\mu_T^2}$$

To examine how good this approximation is, we performed a simple simulation. We generated gamma distributions with μ = 5000 (typical fluorescence intensity in the microarray) and σ = R·μ, R varying from 0.01 to 1.01. We compared the true estimates for E[log(X)] and Var(log(X)) with the Taylor approximation in the range of σ/μ between 0 and 1. Though it generally seems to be a reasonable estimate, as we approach σ/μ =1, the approximation systematically underestimates CV_{logT}.

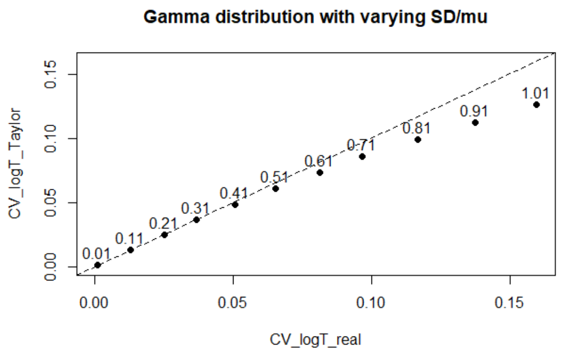

Accepting this approximation, we thus can write:

$$CV_{logT} \stackrel{\text{def}}{=} \max\left(\frac{\sigma_{\text{logT}}}{\mu_{\text{logT}}} \mid \text{U}, \text{M}, \sigma_{\text{M}}, \sigma_{\text{U}}\right) \approx \frac{\sigma_{\text{T}}/\mu_{\text{T}}}{\log\left(\mu_{\text{T}}\right) - \frac{\sigma_{\text{T}}^2}{2 \cdot \mu_{\text{T}}^2}} = \frac{1}{\log(\mu_{\text{T}})/\text{R} - \text{R}/2}; \quad R = \frac{\sigma_{\text{T}}}{\mu_{\text{T}}}$$

We can add small constants for numerical stability:

$$\hat{R} = \frac{\hat{\sigma}_{\text{M}} + \hat{\sigma}_{\text{U}} + \alpha}{\hat{\text{U}} + \hat{\text{M}} + \alpha}; \quad \widehat{\log(\mu_{\text{T}})} = \log\left(\hat{\text{U}} + \hat{\text{M}} + \alpha\right); \ \alpha = 100$$

CV_{logT} greatly increases when a probe retrieves solely background fluorescence (e.g. large noise-to-signal ratio). For probes where a genetic variant on the template DNA leads to probe failure, an ambivalence in signal-to-noise ratio is observed (high CV_{logT} for aa and low CV_{logT} for aA and AA). This translates into a highly bimodal empirical probability density distribution of CV_{logT} across samples for a given CpG, which can be quantified with a bimodality coefficient (BC). The sample bimodality coefficient estimator is computed as:

$$\text{BC}\left(\text{CV}_{\text{logT}}\right) = \frac{\hat{\gamma}_{\text{CV}_{\text{logT}}}^{\ 2} + 1}{\hat{\kappa}_{\text{CV}_{\text{logT}}} + 3 \cdot \frac{(\text{n}-1)^2}{(\text{n}-2)(\text{n}-3)}}$$

Where γ and κ are the sample skewness and excess kurtosis of a given CpG across individuals, respectively. As a rule of thumb, BCs higher than 5/9 (the expected value of BC in a uniform distribution), point towards a bimodal or a multimodal distribution [28].

Finally, probes may fail for other reasons rather than genetic artefacts. For this reason, it is important to have a genetic control. One way is to compute the Spearman/Pearson correlation of CV_{logT} between MZ twins. High correlation would be strong evidence that probe failure has a genetic basis.

4. K-calling

In the heart of the K-caller lies density-based spatial clustering of applications with noise (dbscan) algorithm [29]. Unlike bGMM, dbscan is an incredibly powerful non-parametric clustering algorithm robust to outliers that does not require the number of clusters beforehand. Dbscan localizes points associated to highly dense areas in sample space (core points) and performs subsequent sample aggregation towards these points. Dbscan employs two parameters: *eps*, maximum distance between two samples to be considered as in the same neighbourhood, and *minPts*, minimum number of samples in a

neighbourhood to be considered as a core point. Dbscan also requires a function to compute distance between points; in our case, Euclidean distance was employed.

To benefit from this algorithm, pre-processing is key. For example, in the presence of highly elliptical clusters, dbscan may detect regions of low density and hence, treat a long cluster as two distinct clusters oriented towards its poles. Hence, reducing the ellipticity of clusters can help improve the performance of dbscan. We first compute:

$$\beta = \frac{M}{M+U+100};\ \ R = log_2(M+U+100)$$

Then, R is transformed as:

$$R_{trans} = \frac{R - \min(R)}{\max(R)}$$

Please note that this is not a standard min-max normalisation as the denominator is simply max(R) instead of max(R)-min(R). The purpose of this transformation is to give importance to the R-dimension only when large variation is present in R across samples; like for example, in presence of probe failure. Finally, dbscan is deployed on the (β, R_{trans}) plane.

In order to find the parameters *eps* and *minPts* that show the best performance for the number of samples given at the E-risk cohort, we calibrated dbscan in a training set (943 CpGs forming from one to four clusters). This independent set of markers was built by manually curating U/M plots from random CpGs. We selected parameters to optimize K-calling, written as a multi-class classification machine learning task scored by a macro F_1-score.

5. Scoring benchmark performance

Evaluation of bGMM

In our benchmarking, we employed bGMM with a target cluster number of 2 for sex-chromosomes, which is the expected number of clusters if segregating by sex in the U/M plane and 3 for SNP-targeting probes, the expected number of genotypes. bGMM assigns samples to each cluster; the numbering of the clusters is arbitrary and random (but will be the same between twins if these are classified perfectly by genetics). The following properties are expected from a proper scoring system under the above conditions:

- Approaching zero when the confusion matrix tends to uniformity.
- Equal to one when the confusion matrix is diagonal.

- N/A when all predictions end up in the same cluster.

With all the above properties, we defined the twin assignment agreement as:

$$\frac{1}{n}\sum_{i=1}^{n} \rho{_i}^2_{\text{MZ assigned cluster}}$$

Where ρ^2 is the Pearson correlation coefficient squared between assigned clusters between MZ twins.

Evaluation of BC(CV_{logT}) and cor_{MZ}(CV_{logT})

The bimodality coefficient of a uniform distribution is of 5/9. Bimodality coefficients above 5/9 can serve as evidence for bimodality. However, bimodality in CV_{logT} can surge from spontaneous probe failure unrelated to genetic artefacts. In order to control for genetics, we also set-up a conservative threshold for $cor_{MZ}(CV_{logT})$ of 0.8. Hence, we defined proportion of genetics-related probe failure as:

$$\frac{1}{n}\sum_{i=1}^{n} \mathbf{1}_{\text{BC}_i(\text{CV}_{\text{logT}}) > 5/9} \cdot \mathbf{1}_{\text{cor}_i(\text{CV}_{\text{logT}}) > 0.8}$$

where $\mathbf{1}_{condition}$ is the indicator function, equal to one when condition is met and equal to zero, elsewise.

Evaluation of the K-caller

Here, we simply defined the proportion of correct cluster number prediction as:

$$\frac{1}{n}\sum_{i=1}^{n} \mathbf{1}_{\text{k}_\text{i} = \text{K}_{\text{Exp}}}$$

where $\mathbf{1}_{condition}$ is the indicator function, equal to one when condition is met and equal to zero, elsewise and where K_{Exp} is the expected number of clusters.

6. Annotation of 450K/850K probes dbSNP151

As for genetic variant annotation, we used the 00-common_all.vcf file contained at the National Center for Biotechnology and Information (NCBI) File Transfer Protocol (FTP) site ftp://ftp.ncbi.nlm.nih.gov/snp/organisms/human_9606_b151_GRCh37p13/VCF/. This file contains single-nucleotide variants and insertions and deletions of a germline

origin with a minor allele frequency higher or equal to 1 % in at least one major population, with at least two unrelated individuals having the minor allele. Bed files containing the locations of probes in the 450K and 850K were parsed in R from annotation R-packages IlluminaHumanMethylation450kanno.ilmn12.hg19 and IlluminaHumanMethylationEPICanno.ilm10b2.hg19, respectively. Three coordinate files were created: CpG sites, type I probe SBE sites and probe binding sites excluding the CpG site (see scheme below for more details). Probe Coordinates were intersected employing bedtools (v2.29.2) with subcommand intersect as in:

bedtools intersect -wa -wb -a 00-common_all.vcf -b coords.bed > intersect.bed

As bedtools uses intervals of the form (a, b], careful delimitation of the coordinates is required (more details below).

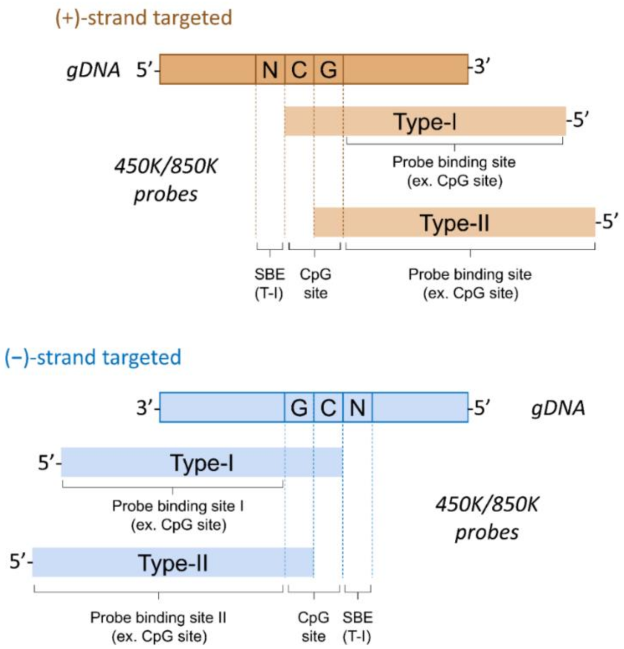

Defining p as the position of the targeted cytosine in the CpG and L, as the probe length we obtain the following coordinates:

Type I probe SBE sites			
		Closed Interval	*Open left Interval*
(−)	*T-I*	[p+2, p+2]	(p+1, p+2]
	T-II	NA	NA
(+)	*T-I*	[p-1, p-1]	(p-2, p-1]
	T-II	NA	NA

CpG sites			
		Closed Interval	*Open left Interval*
(−)	*T-I*	[p, p+1]	(p-1, p+1]
	T-II	[p, p+1]	(p-1, p+1]
(+)	*T-I*	[p, p+1]	(p-1, p+1]
	T-II	[p, p+1]	(p-1, p+1]

Probe binding sites excluding the CpG site			
		Closed Interval	*Open left Interval*
(−)	*T-I*	[p-L+2, p-1]	(p-L+1, p-1]
	T-II	[p-L+1, p-1]	(p-L, p-1]
(+)	*T-I*	[p+2, p+L-1]	(p+1, p+L-1]
	T-II	[p+2, p+L]	(p+1, p+L]

To verify coordinates of genetic variants in the output file, we additionally computed the distance between the variant and CpG coordinate. Expected results were:

Distance to the CpG site’s C					
Type I probe SBE sites	CpG sites	Probe binding sites excluding the CpG site			
		I (−)	II (−)	I (+)	II (+)
(1, 2)	(0, 1)	[1, 48]	[1, 49]	[2,49]	[2, 50]

7. Variant calling and minor allele frequency estimation from methylation data

Probe failure only occurs when both alleles are the probe-failing allele. Hence, two clusters are formed: aa and Aa/AA (dominance). To estimate allelic frequencies, we need to assume Hardy-Weinberg equilibrium (HWE), and hence:

$$q = \sqrt{f(aa)};\ \ p = 1 - q$$

K = 3, (AA, BB, AB)

when alleles are codominant, we can estimate allelic frequencies without assuming HWE as:

$$p = f(AA) + \frac{1}{2} \cdot f(AB); \quad q = 1 - p$$

K = 4, (A/a, U/M)

when two variants artefactually dominate the measured methylation state and these are dominant (A>a; A: active, a: inactive) and codominant (M/U; M: methylated, U: unmethylated), respectively, and with epistasis (aU = 0, aM = 0), we can write the following haplotypes:

Genotype	Cluster
AM, AM	Methylated
AM, AU	Half-Methylated
AM, aM	Methylated
AM, aU	Methylated
AU, AU	Unmethylated
AU, aM	Unmethylated
AU, aU	Unmethylated
aM, aM	Probe failure
aM, aU	Probe failure
aU, aU	Probe failure

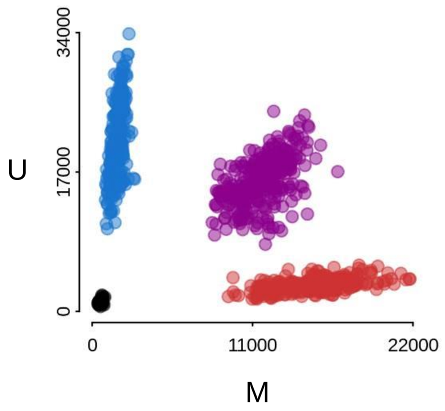

Defining haplotype frequencies as $\alpha = f(AM)$; $\beta = f(AU)$; $\gamma = f(aM)$; $\delta = f(aU)$, we get:

	AM	AU	aM	aU
AM	αα	αβ	αγ	αδ
AU	αβ	ββ	βγ	βδ
aM	αγ	βγ	γγ	γδ
aU	αδ	βδ	γδ	δδ

We can write the following equations:

$$\alpha + \beta + \gamma + \delta = 1$$

$$f(k_1) = \gamma^2 + \delta^2 + 2\gamma\delta = (\gamma + \delta)^2; \;\; \gamma + \delta = \sqrt{f(k_1)}$$

$$f(k_2) = \beta^2 + 2\beta\gamma + 2\beta\delta = \beta^2 + 2\beta(\gamma + \delta)$$

$$f(k_3) = \alpha^2 + 2\alpha\gamma + 2\alpha\delta = \alpha^2 + 2\alpha(\gamma + \delta)$$

$$f(k_4) = 2\alpha\beta$$

Although $f(k_1) + f(k_2) + f(k_3) + f(k_4) = 1$ could be thought of another equation, it can be derived from the five prior equations:

$$f(k_1) + f(k_2) + f(k_3) + f(k_4)$$
$$= \alpha^2 + \beta^2 + \gamma^2 + \delta^2 + 2\alpha\beta + 2\alpha\gamma + 2\alpha\delta + 2\beta\gamma + 2\beta\delta + 2\gamma\delta$$

$$= (\alpha + \beta + \gamma + \delta)^2 = 1$$

It is to be noted that γ = f(aB) and δ = f(aC) always appear as $(\gamma + \delta)$ (hence, confounded): both haplotypes give rise to probe failure and cannot be differentiated. Although the system of equations can be solved analytically, we rather deployed the Nelder-Mead gradient-free method. This option is often preferred in statistics for parameters estimation problems where functions are subject to noise. As a result, there are two variables two optimize; for example, (α, β) (γ+δ is determined to be equal to 1- α - β), given inputs $[f(k_1), f(k_2), f(k_3), f(k_4)]$. We can write the following cost function:

$$Cost_A = [\beta^2 + 2\beta\sqrt{f(k_1)} - f(k_2)]^2$$

$$Cost_B = [\alpha^2 + 2\alpha\sqrt{f(k_1)} - f(k_3)]^2$$

$$Cost_C = [2\alpha\beta - f(k_4)]^2$$

$$Cost_D = [\alpha + \beta + \gamma + \delta - 1]^2$$

$$Cost(\alpha, \beta \mid k_1, k_2, k_2, k_3) = Cost_A + Cost_B + Cost_C + Cost_D$$

We finally deployed the Nelder-Mead algorithm with initial conditions $\alpha_0 = \beta_0 = \frac{1-\sqrt{f(k_1)}}{2}$, with a cost function $Cost(\alpha, \beta \mid k_1, k_2, k_3)$ to optimize parameters (α, β) such that the cost function is minimized.

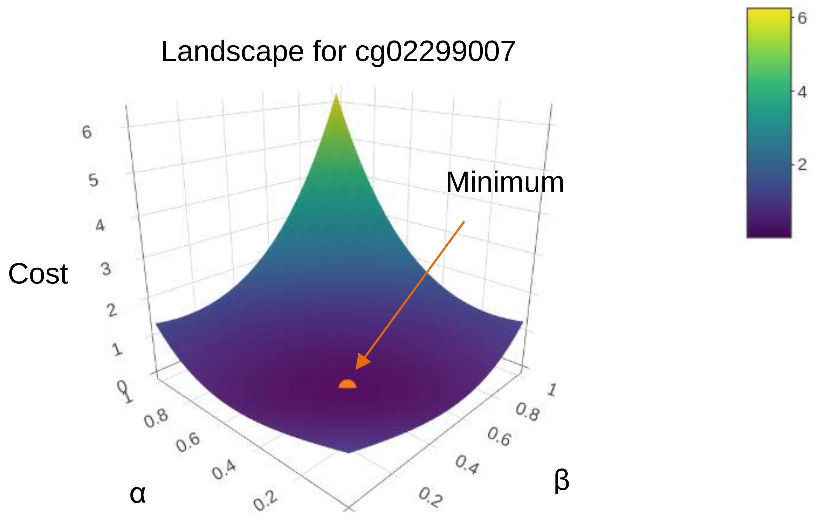

Interaction with imprinting gives rise to similar patterns to X-inactivation but whose clusters are not correlated with sex: no probe failure, apparent codominance but with a missing genotype that cannot be simply explained by a low minor allele frequency given the large excess of heterozygotes.

Maternal imprinting<br>*paternal allele is M/M’*<br>*maternal allele is U/M’*		Paternal imprinting<br>*maternal allele is M/M’*<br>*paternal allele is U/M’*	
$p^{M}m^{M'}$	Methylated	$m^{M}p^{U}$	Half-Methylated
$p^{M}m^{U}$	Half-Methylated	$m^{M}p^{M'}$	Methylated
$p^{M'}m^{M'}$	Methylated	$m^{M'}p^{U}$	Half-Methylated
$p^{M'}m^{U}$	Half-Methylated	$m^{M'}p^{M'}$	Methylated

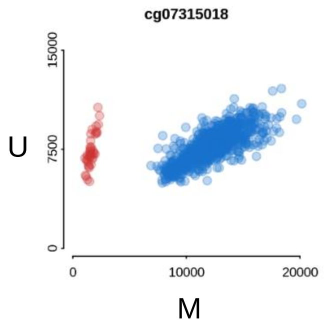

$$\alpha = f(M'M) = f(M'U);\;\; f(M'M') = \beta;\;\; f(MU) = \gamma$$

$$f(k_1) = f(M'M) + f(M'M') = \alpha + \beta$$

$$f(k_2) = f(MU) + f(M'U) = \gamma + \alpha$$

$$f(M') = f(M'M') + \frac{1}{2}f(M'M) + \frac{1}{2}f(M'U) = \beta + \frac{\alpha}{2} + \frac{\alpha}{2} = \alpha + \beta = f(k_1)$$

K = 2, variant = U’, with imprinting

Maternal imprinting<br>*paternal allele is M/U’*<br>*maternal allele is U/U’*		Paternal imprinting<br>*maternal allele is M/U’*<br>*paternal allele is U/U’*	
$p^{M}m^{U}$	Half-Methylated	$m^{M}p^{U}$	Half-Methylated
$p^{M}m^{U'}$	Half-Methylated	$m^{M}p^{U'}$	Half-Methylated
$p^{U'}m^{U}$	Unmethylated	$m^{U'}p^{U}$	Unmethylated
$p^{U'}m^{U'}$	Unmethylated	$m^{U'}p^{U'}$	Unmethylated

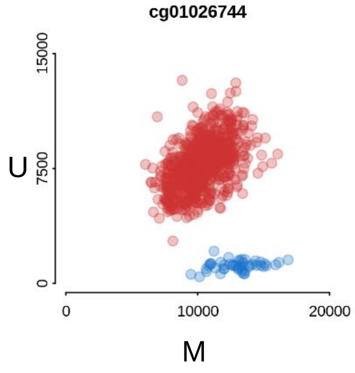

$$\alpha = f(U'U) = f(U'M);\;\; f(U'U') = \beta;\;\; f(MU) = \gamma$$

$$f(k_1) = f(U'U') + f(U'U) = \alpha + \beta$$

$$f(k_2) = f(U'M) + f(MU) = \alpha + \gamma$$

$$f(U') = f(U'U') + \frac{1}{2}f(U'M) + \frac{1}{2}f(U'U) = \beta + \frac{\alpha}{2} + \frac{\alpha}{2} = \alpha + \beta = f(k_1)$$

8. Identification of representative examples

Sex chromosomes and SNP controls probes

U/M plots for random sex-chromosome targeting probes were visualized. After looking at hundreds of U/M plots, representative examples were chosen. For the 65 SNP-targeting control probes, their aspect was very consistent; so, choosing a representative example was trivial.

CpG/SBE-SNPs

From the bedtools output, we annotated biallelic variants into 16 categories of CpG/SBE-SNPs. We ranked by MAF and selected the top markers for each category as those were easier to verify. Some logic applies to the highlighted example of type-I (+) SNP C↔T, type-I (−) SNP G↔A.

CpG/SBE/probe-indels

From the bedtools output, we restricted our analysis to indels. We ranked by MAF and scanned through hundreds of U/M plots to identify all patterns.

Non-annotated genetic artefacts

We searched for probes displaying BC(CVlogT) > 5/9, corMZ(CVlogT) > 0.8 and K_{pred} = 2, that were not included in any of our genetic artefact annotation. By examining probe localization, we found instances of several contiguous probe failure happened on the same samples for several probes, in agreement between MZ twins. This corresponded to unannotated large-indels, CNVs and SVs.

CpGs sites with two CpG/SBE-SNPs

From the 16-category classification of CpG/SBE-SNPs, we selected CpGs with more than one associated SNP. For the example, we scanned for CpGs whose both variants had high MAF.

Tri-allelic CpG/SBE-SNPs

In the making of the 16-category classification of CpG/SBE-SNPs, we had to previously localize tri-allelic SNPs for exclusion. At this stage, we focused on this list and search for variants whose three alleles had allelic frequencies high enough to be observed in E-risk.

Genetic artefacts interacting with imprinting

We firstly theorized how imprinting could impact a U/M plot. With knowledge at hand, we extracted a list of all CpGs associated to imprinted genes and examined one-by-one.

Genetic artefacts interacting with ChrX methylation biology

We focused on the X-probes within the 16-category classification of CpG/SBE-SNPs. By making U/M plots highlighting sex by colour we identified a set of unexpected patterns (clustering patterns different from X-inactivation/hypermethylation/escape). *Post-hoc*, we tried to explain how the U/M plot was formed in these cases.

Cross-reactive probes

We plotted hundreds of U/M plots of known cross-reactive probes [6, 7] and identified outlier patterns. We attempted to model the behaviour of these sites *post-hoc*.

Tissue-specific genetic artefacts.

With a dataset of matched saliva-blood samples, we searched for sites with high IQR in one tissue but not on the other. By visualizing U/M plots in both tissues, a set of examples could be easily located. Post-hoc and to exclude potential tissue-specific meQTLs, we identified the causal underlying genetic variant that explained the artefactual U/M plot.

Identification of a true-positive meQTL

With the help of the heritability ranking, we visualized co-methylation plots in decreasing order of heritability. The meQTL at NINJ2 intron was chosen for its availability of neighbour probes at a close distance and the strong presence of co-methylation across 4 probes. *A posteriori*, we searched whether these CpGs had been reported as meQTLs.

9. SNP imputation

In the validation of the NINJ2 meQTL, we had to impute our putative cis-causal SNP as it was not available in the SNP array. To do so, we transformed the file "HumanOmni5-4v1-1_A.csv" from GSE31438 (GEO) to GEN format with a custom script. We then ran Impute2 v2.3.2 [86], with the following arguments:

```
impute2 \
 -m $where/genetic_map_chr12_combined_b37.txt \
 -h $where/ ALL.chr12.phase3_shapeit2_mvncall_integrated_v4.20130502.genotypes.breakmulti.2504Samples.impute.hap \
 -l $where/1000GP_Phase3_chr12.legend \
 -g $where2/chr12.study.gens \
 -strand_g $where2/chr12.study.strand \
 -Ne 20000 \
 -int 673462 772945 \
 -o ./chr12.one.phased.impute2
```

10. Guidelines on how to repurpose UMtools

A wide range of R-packages have already been developed to analyze data from Illumina's DNA methylation microarray platforms such as minfi, wateRmelon, ENmix, ChAMP, lumi, methylumi, meffil, EWAStools, sva, etc. In brief, these standard tools cover parsing IDATs, QC, batch effect correction, pre-processing and normalizations, exploratory analysis and differential methylation analysis among other tasks (cell type composition, sex or age prediction, to name a few).

UMtools does not aim to replace any of the aforementioned packages; in fact, it uses minfi as a dependency. UMtools focuses on the low-level analysis of Illumina DNA methylation microarray data, at the level of fluorescence intensities and can hence supplement some QC tasks, exploratory and *post-hoc* analysis. DNA methylation microarrays are particularly popular in epigenome-wide association studies (EWAS) to discover novel biomarkers associated with a phenotype of interest. For this reason, we focus this guideline especially towards EWAS users.

1) Sample quality control

CV_{logT} serves as a quantitative alternative to detection p-values. For example, multi-dimensional scaling on the CV_{logT} matrix (for example, obtained by running minfi::mdsPlot) could reveal how noise-to-signal ratio varies between samples and hence answer questions like: do we see batch

effects in the noise-to-signal ratio? Samples subject to a high average CV_{logT} across CpGs can be filtered out via outlier analysis.

We always recommend predicting sex, comparing with reported sex and excluding samples when these two do not match to avoid sample mix-ups. This is normally done based on total fluorescence intensities in ChrX/Y (Bg noise of Y-probes and double total intensity of X-probes in females). However, cross-reactive probes interfere in this test. Cross-reactive Y-probes can be detected via $BC(CV_{logT}) < 5/9$ in a dataset that includes both males and females. This can be performed in R by choosing which indexes to include in the minfi:::.getSex function as in:

CN <- minfi::getCN(rgSet)
minfi:::.getSex(CN = CN, xIndex = xIndex, yIndex = yIndex, cutoff = -2)

The proposed features should not replace standard quality control such as excluding outlier samples based on control probes (with special attention to bisulfite conversion controls) and detection p-values or checking for batch effects at the DNA methylation scale.

2) Probe quality control

We advocate not to use *in silico* predicted probe-exclusion lists but rather a probe flagging system (more information available in the Discussion section of our manuscript). For example, $BC(CV_{logT}) > 5/9$ can be used to flag probes potentially displaying ambivalence in probe failure (due to genetic artefacts or simply unreliable performance). Also, predicted number of clusters (output by the K-caller) > 1, can be used to flag probes with higher order clusters in the U/M plane. These could be both genetic artefacts or meQTLs. Also, UMtools contains a wide range of annotations that may be useful to EWAS researchers such as:

i) data(annot_450K): Genetic variants associated to Illumina Infinium HumanMethylation450 Beadchip probes based on dbSNP151

ii) data(annot_EPIC): Genetic variants associated to Illumina Infinium MethylationEPIC Beadchip probes based on dbSNP151

iii) data(classification_CpG_SNP_450K): Classification of CpG/SBE-SNPs in the Illumina Infinium HumanMethylation450 Beadchip microarray

iv) data(classification_CpG_SNP_EPIC): Classification of CpG/SBE-SNPs in the Illumina Infinium MethylationEPIC Beadchip microarray

v) data(CR_probes): List of in silico-predicted cross-reactive probes in the Illumina Infinium HumanMethylation450 Beadchip microarray

vi) data(triallelic_CpG_SNP_450K): Tri-allelic SNPs associated to Illumina Infinium HumanMethylation450 Beadchip probes

vii) data(triallelic_CpG_SNP_EPIC): Tri-allelic SNPs associated to Illumina Infinium MethylationEPIC Beadchip probes

For more details, please see Tutorial at https://github.com/BenjaminPlanterose/UMtools. We envision *in silico* predictions and data-driven information side-by-side (and properly labelled as such) as in:

Probe			Data-driven information			*In silico* prediction				
CpG	Chr	Pos	K-calling	CV_{logT}	$BC(CV_{logT})$	CR	CpG/SBE-SNP	CpG/SBE-indel	probe-SNP	probe-indel
cg23411065	chr17	39705458	1	0.05 ±0.02	0.31	FALSE	NULL	NULL	NULL	NULL

3) *Post-hoc* analysis

After significance testing, the numbers become more manageable, and the targeted tools included in UMtools can hence be used. We recommend making U/M plots for each significant result coloring by the phenotype of interest for visual examination; strong effect sizes should be obvious in the U/M plane; for example, age-associated CpGs display a change in slope in the U/M plane. In general, it is good practice to go back to the raw data to check whether the obtained results are a consequence of data preprocessing. If not obvious on the raw data, it is very important to ensure that the preprocessing strategy is optimal to avoid false positive results.

Particularly, the formation of clusters in the U/M plane should alert the researcher that genetics may be playing a role in the variation observed, either artefactual or genuine. To distinguish between both scenarios, we firstly recommend consulting probe design and checking for potential genetic artefacts by looking at the current version of dbSNP and by cross-referencing with Table 1. Finding which sample belongs to which cluster can be performed by bGMM and may be helpful in finding out the underlying mechanism at a given probe (especially if genetic information is also available). For a data-driven approach, co-methylation plots can help see whether neighboring CpGs are co-methylated with the significant hit. Interpreting co-methylation plots requires care and details can be found in the Discussion of our manuscript. But even if no clusters are observed in the U/M plane, co-methylation with nearby CpGs should be observable for any CpG displaying inter-individual variation (as long as there are available probes within the co-methylation windows). Additionally, we also recommend visiting higher CpG resolution WGBS data to check whether the region presents inter-individual variation.

Finally, we recommend bearing in mind the possibility of genetic artefacts when using DNA methylation microarrays on specimens where the expected genetic profile differs from that considered in the design of the microarray. For example, in cancer-control EWAS genetic artefacts may be inherent to the genetic rearrangements associated to tumorigenesis; hence, it is possible that genetic differences translate into an observed change in the artefactual methylation read-out. If the genetic artefact is not correctly identified, it may be considered a differentially methylated site rather than simply a mutation. It is good practice to check whether any technical measures are associated to the phenotype of interest. For example, it is possible to test whether noise-to-signal ratio at a significant hit via CV_{logT} is correlated with the phenotype of interest. If so, it is possible that the DNA methylation differences are artefactual and that solely the underlying genetic template differs.
