# Supplementary material for: Revisiting genetic artifacts on DNA methylation microarrays exposes novel biological implications
Source: Genome Biol. 2021 Sep 21;22:274. doi: 10.1186/s13059-021-02484-y (PMC8454075; doi:10.1186/s13059-021-02484-y)
Supplement: Supplementary file 3 — Additional file 3. Review history. [file 13059_2021_2484_MOESM3_ESM.docx]

Review History

**First round of review**

**Reviewer 1**

**Are you able to assess all statistics in the manuscript, including the appropriateness of statistical tests used?**

No, I do not feel adequately qualified to assess the statistics.

**Comments to author:**

This is an interesting, partially novel approach to investigate genetic effects in Illumina microarray data. The authors describe UmTools a package building on the use of raw signal intensities obtained from the IDAT files. The use co-methylation as an approach to dissect MAF's from mQTLs.

The work represents a very comprehensive overview of potential artefacts generated in 450K/EPIC analyses. In this sense the paper lines up with a number of previous papers (some are cited). Their novel approach and R-package has a clear potential of being a novel and detailed application to identify and investigate genetic effects on DNA methylation. However the general practical usability of the R-package remains a bit unclear.

Overall the paper is written in a complicated way mixing results and interpretations. In its present form the paper does not convince the reader how to use the tools for his/her dataset/analysis questions.

Some of the claims in the paper are not yet underpinned by comparative analyses:

*	The authors should concentrate to show the effect of their correction package on standard epigenome-wide association studies. In the paper they only apply their tool to monozygotic twin data, for which the genetic dependencies are known. It would be interesting to see how this approach performs in a general design of a study and what will be the added value.

*	Furthermore it remains to be discussed how the tool relate/matches/extends (to) standard tools for the analysis of microarray data, such as RnBeads, ChAMP, minfi, or watermelon. This should at least be discussed.

*	The Code is not yet available on GitHub. To judge on the utility of the method, the code should be freely available and associated tutorials also accessible (https://github.com/BenjaminPlanterose/UMtools does not exist). Also, a submission to Bioconductor might increase the visibility of the tool.

*	Overall the aims of the study are extensively "outlined" - this can be shortened and streamlined.

*	The entire analysis/results part is heavily overloaded by interpretations - again more focus would help.

*	The text is full of sentences with commentary inserts and spiked with abbreviations which makes reading very difficult and blurs the messages.

**Reviewer 2**

**Are you able to assess all statistics in the manuscript, including the appropriateness of statistical tests used?**

Yes, and I have assessed the statistics in my report.

**Comments to author:**

This manuscript describes a new set of computational tools, along with comprehensive data analysis, for detecting and understanding the genetic artefacts on DNA methylation microarrays. The authors provided a set of tools, UMtools, for quantifying genetic artefacts based on fluorescence intensity signals, building upon U/M plots and bGMM clustering. The authors also performed in-depth analysis on multiple DNA methylation data including those on monozygotic twins and empirically validated the results. In addition, the authors perform an in-depth analysis on a methylation site at the first intron of NINJ2 gene and provides a mechanical model for understanding how the SNP rs34038797 influences the methylation there.

Overall, this a very nice study that provides important tools for detecting the genetic artefacts on the DNA methylation assay and challenges current practices that over-rely on probe exclusion lists in methylation analysis. The paper is well written, and the results are comprehensive. I only have a few minor comments:

1. Some of the genetic artefacts listed classified in Table 1 appears to represent genuine genetic effect. Specifically, if the SNP on the CpG site directly disrupt the CpG site, then the presence of the SNP would lead to a lack of methylation, thus representing genuine genetic effect. On the other hand, if the SNP on/near the CpG site does not disrupt the CpG site but only influence the hybridization of the probe, then the methylation level of the CpG site is not influence the SNP. Instead the SNP would only influence the ability to detect the methylation, thus representing genetic artefacts. It would be critical to distinguish these two types of SNPs in the Table and the paper. It would would be helpful to provide additional discussions on this important issue of genuine genetic effect vs genetic artefact.

2. In Figure 3C, the authors show that the SNPs beyond the CpG/SBE site may also manifest themselves as genetic artefacts via probe failure. I was wondering whether SNPs on the 5' of the CpG site would also influence the effectiveness of probe hybridization, thus leading to artefacts in methylation level measurements. A similar plot extending the x-axis towards 5' would be valuable.

3. It would be helpful to add additional details to the Figure legend, so that each Figure becomes readable without the readers going back to the main text. Right now, I have to go back to the Results section and even the Methods section in order to fully understand each Figure.

**Authors Response**

**Point-by-point responses to the reviewers’ comments:**

Reviewer #1:

This is an interesting, partially novel approach to investigate genetic effects in Illumina microarray data. The authors describe UmTools a package building on the use of raw signal intensities obtained from the IDAT files. The use co-methylation as an approach to dissect MAF's from mQTLs. The work represents a very comprehensive overview of potential artefacts generated in 450K/EPIC analyses. In this sense the paper lines up with a number of previous papers (some are cited). Their novel approach and R-package has a clear potential of being a novel and detailed application to identify and investigate genetic effects on DNA methylation.

*Reply: We thank the reviewer on these positive comments.*

1. However the general practical usability of the R-package remains a bit unclear. Overall the paper is written in a complicated way mixing results and interpretations. In its present form the paper does not convince the reader how to use the tools for his/her dataset/analysis questions.

*Reply: We appreciate the reviewer’s comment on how to improve the readability of our manuscript and increase the usability of our described findings and tools. Firstly, we would like to stress that some of our results may be hard to interpret. Aiming to ensure that all results are accessible and interpretable by the broad readership of Genome Biology, we found at times necessary to place some explanations alongside findings. Not doing so would grant the manuscript hard to comprehend by non-specialists. Secondly, given the nature of our manuscript (submitted as a research article rather than a method paper), we placed more emphasis on the uncovered science and understanding of genetic artefacts rather than the methods themselves, which drove our writing style. Of course, achieving our goal was only possible via the highly repurposable tools we developed, which we want to share with the community. To accommodate the reviewer’s demands, we have now provided a section under the revised Supplementary Methods on how the reader can practically use UMtools in their datasets and analysis questions (lines 438-524).*

2. Some of the claims in the paper are not yet underpinned by comparative analyses: The authors should concentrate to show the effect of their correction package on standard epigenome-wide association studies. In the paper they only apply their tool to monozygotic twin data, for which the genetic dependencies are known. It would be interesting to see how this approach performs in a general design of a study and what will be the added value.

*Reply: We respectfully disagree with the reviewer on that UMtools is a correction package aimed towards EWAS. We have envisioned UMtools as a set of tools that can qualify DNA methylation microarray probes for a wide range of purposes well beyond EWAS; thus, we do not see fit to include any EWAS specific follow up such as an association comparative analysis (EWAS employing current norm Vs UMtools). In this study, we aimed to build an atlas on how genetic artefacts interfere with the Infinium assay and to challenge current practices that over-rely on probe exclusion lists. To do so, we developed the UMtools R-package and firstly validated it by employing monozygotic twins as genetic controls. Although none of the tools actually require monozygotic twins, these were used to benchmark their performance exactly because of their genetic dependencies. In other words, validating UMtools on population data used in typical EWAS would grant a substandard experimental design, as it could not reveal potential issues in reproducibility. In fact, it is not true that we only applied our UMtools to monozygotic twin data. As shown at the datasets section under Methods (lines 657-700), we employed a total of ten 450K datasets, eight of which do not include monozygotic twins.*

*Nonetheless, given how particularly popular DNA methylation microarrays are in EWAS (to discover novel biomarkers associated with a phenotype of interest), we understand why the reviewer is particularly focused on EWAS applications. We did claim that the tools developed in our study may be re-purposed for other goals; in EWAS for example, these could be used to validate significant hits, although this was not the main goal of the study. In particular, we highlighted in the discussion the example of the methylation of cg01097406, which had been previously shown to be significantly associated to homocysteine levels via EWAS. UMtools assisted in revealing that this variant is in fact an meQTL that is probably significant in EWAS because it is in linkage disequilibrium with a genetic variant, that has been reported as significant in GWAS (so called proxy-GWAS). We hope that the new section under the revised Supplementary Methods on the UMtools guidelines (lines 438-524), especially focused for EWAS research, will make it clear to the reviewer how our approach performs in a general design of a study and what the added value is.*

3. Furthermore it remains to be discussed how the tool relate/matches/extends (to) standard tools for the analysis of microarray data, such as RnBeads, ChAMP, minfi, or watermelon. This should at least be discussed.

*Reply: The reviewer mentioned four popular R-packages employed for data analysis of DNA methylation microarray data, with the aim to question how UMtools adds to this complex ecosystem. We are happy to clarify this to the reviewer and readers, hence we added a comment on how UMtools related to other packages in the revised discussion section of our manuscript (lines 515-521).*

*In brief, these standard tools cover parsing IDATs, QC, pre-processing, exploratory analysis and differential methylation analysis among other tasks (cell type composition, sex or age prediction, to name a few). UMtools does not aim to replace any of the aforementioned packages; in fact, it uses minfi as a dependency. Instead, UMtools can serve as a supplement for some QC tasks, exploratory and post-hoc analysis. In the writing of this manuscript, we researched for similar features in the literature and we only found minfi::gaphunter, minfi::detectionP, EWAStools::detectionP and MethylToSNP::MethylToSNP. The results of the benchmarking are extensively described in the results section of our manuscript and shows how UMtools functions has an improved performance to those tools currently available (lines 226-243). Additionally, none of the mentioned R-packages include:*

*- tools to perform sample assignation in the U/M plane when the number of clusters in known. UMtools includes Bivariate Gaussian Mixture models (bGMM) for this goal.*

*- tools to perform sample assignation in the U/M plane when the number of clusters in not known. UMtools includes a K-caller for this goal.*

*- modules to compute the coefficient of variation of the logarithm of the total signal (CVlogT) nor the bimodality of Coefficient of variation of the logarithm of the total signal (BC(CVlogT)) as these concepts are completely novel which UMtools includes.*

4. The Code is not yet available on GitHub. To judge on the utility of the method, the code should be freely available and associated tutorials also accessible (https://eur01.safelinks.protection.outlook.com/?url=https%3A%2F%2Fgithub.com%2FBenjaminPlanterose%2FUMtools&data=04%7C01%7Cb.planterosejimenez%40erasmusmc.nl%7Ca723ab1fddcf473bf62308d93578da3c%7C526638ba6af34b0fa532a1a511f4ac80%7C0%7C0%7C637599615432802171%7CUnknown%7CTWFpbGZsb3d8eyJWIjoiMC4wLjAwMDAiLCJQIjoiV2luMzIiLCJBTiI6Ik1haWwiLCJXVCI6Mn0%3D%7C1000&sdata=iKu7MaPdOtoQmI5N0f1Xr1PXz5c7iZWBrL%2BjKfw9WKI%3D&reserved=0 does not exist). Also, a submission to Bioconductor might increase the visibility of the tool.

*Reply: The Github project for UMtools is now available, including a tutorial that walks through the installation and documentation of the functions included in the R-package (https://github.com/BenjaminPlanterose/UMtools). For now, we prefer to host the R-package on Github, as the Github issue portal (https://github.com/BenjaminPlanterose/UMtools/issues) provides fast, dynamic and convenient communication with end-users. This way, we may instantaneously add new features requested by UMtools users. This strategy has been followed before with other popular R-packages in epigenomics, such as EWAStools (https://github.com/hhhh5/ewastools) [Heiss, JA, Just, AC. (2019). Clin Epigenetics 11(1):15]. However, we will take upon the reviewer’s suggestion and consider submitting UMtools in the future also to Bioconductor, depending on the R-package’s demand.*

5. Overall the aims of the study are extensively "outlined" - this can be shortened and streamlined. The entire analysis/results part is heavily overloaded by interpretations - again more focus would help. The text is full of sentences with commentary inserts and spiked with abbreviations which makes reading very difficult and blurs the messages.

*Reply: We regret that the reviewer does not find that the aims of our manuscript have been streamlined concisely. As priorly discussed, we opted for including our interpretation of the findings alongside our results, as we believe that these at times required hard interpretations. We have also made an effort to use standard abbreviations when possible and have equipped the manuscript with a tool summary on Table 1 and an abbreviation section to ease the reading. We agree with the reviewer that some sections include many abbreviations that may result in the blurring of the message; in such paragraphs nonetheless, abbreviations make reference to tools/methods developed in this manuscript. However, we have attempted to increase the clarity of the manuscript in the best of our abilities despite that no specific examples or line numbers were included (lines 141-157, 174-179, 191-192). Moreover, we would like to point out to the editors that reviewer 2 finds our manuscript well written, which has encouraged us not to change the style of writing, contrary to reviewer 1. In the end, it should be up to the authors and their preference on style how to present a manuscript, and we hope the editors will agree with us.*

Reviewer #2:

This manuscript describes a new set of computational tools, along with comprehensive data analysis, for detecting and understanding the genetic artefacts on DNA methylation microarrays. The authors provided a set of tools, UMtools, for quantifying genetic artefacts based on fluorescence intensity signals, building upon U/M plots and bGMM clustering. The authors also performed in-depth analysis on multiple DNA methylation data including those on monozygotic twins and empirically validated the results. In addition, the authors perform an in-depth analysis on a methylation site at the first intron of NINJ2 gene and provides a mechanical model for understanding how the SNP rs34038797 influences the methylation there. Overall, this a very nice study that provides important tools for detecting the genetic artefacts on the DNA methylation assay and challenges current practices that over-rely on probe exclusion lists in methylation analysis. The paper is well written, and the results are comprehensive. I only have a few minor comments:

*Reply: We thank the reviewer on these very positive comments.*

1. Some of the genetic artefacts listed classified in Table 1 appears to represent genuine genetic effect. Specifically, if the SNP on the CpG site directly disrupt the CpG site, then the presence of the SNP would lead to a lack of methylation, thus representing genuine genetic effect. On the other hand, if the SNP on/near the CpG site does not disrupt the CpG site but only influence the hybridization of the probe, then the methylation level of the CpG site is not influence the SNP. Instead the SNP would only influence the ability to detect the methylation, thus representing genetic artefacts. It would be critical to distinguish these two types of SNPs in the Table and the paper. It would would be helpful to provide additional discussions on this important issue of genuine genetic effect vs genetic artefact.

*Reply: We appreciate the reviewer’s comment, and we are happy to elaborate on this topic. We agree that it is critical to distinguish between these terms. To account for the reviewer’s comment and to avoid any future confusions by our readers, we have now included a thorough definition of genetic artefacts in the revised results and discussion section of our manuscript (lines 141-155 and lines 575-585, respectively), which we also outline below.*

*The Infinium’s assay output is a methylation ratio computed as β=M/(M+U). With this in mind, disruption of the CpG site by a SNP can generate three possible outcomes:*

*i) SNP is interpreted as the methylated epiallele (artefactual under any perspective)*

*ii) Probe failure: If homozygous for the probe failing allele, fluorescence background is acquired in both channels of roughly the same scale, and hence, the reported β ≈ 0.5. As the CpG site does not exist in either of the alleles and given that this result does not necessarily correlate with the methylation status of the region, this outcome is uncontroversially artefactual.*

*iii) SNP is interpreted as the unmethylated epiallele: if the CpG does not exist because of the disruption caused by a SNP, the targeted cytosine cannot be methylated by definition. Thus, when the Infinium assay reports an unmethylated status for such a kind of allele, it could be considered to measure a real outcome rather than an artefact (it cannot be methylated if it does not exist). However, we cannot agree with this point of view: being unable to distinguish whether a cytosine of interest is unmethylated or inexistent should be of concern to any researcher and hence, be labelled as an artefact.*

*In the light of genetic diversity, we personally advocate for a DNA methylation locus-centric point of view, rather than simply focusing on targeted CpGs (which may exist for some individuals or not): do we always measure the methylation status of the genomic region correctly? If this is not the case because of a genetic artefact interfering with the Infinium assay one way or another, then this must be a genetic artefact. Finally, we have reserved the expression of genuine genetic effects to meQTLs: a genetic variant that affects the methylation status of a region (encompassing tens to hundreds of CpGs). This is clearly a different phenomenon than losing a CpG site to a SNP, and hence, we prefer to keep it this way. Though we do understand that losing CpG sites can affect the methylation status of a region, but this is not what is being measured in the Infinium assay.*

2. In Figure 3C, the authors show that the SNPs beyond the CpG/SBE site may also manifest themselves as genetic artefacts via probe failure. I was wondering whether SNPs on the 5' of the CpG site would also influence the effectiveness of probe hybridization, thus leading to artefacts in methylation level measurements. A similar plot extending the x-axis towards 5' would be valuable.

*Reply: Indeed, in our study we referenced distance with respect to the 3’-end when examining the influence of SNPs in probe-binding positions. In the Infinium assay, hybridization is not sufficient to generate a signal: a signal can only be generated if a single-base extension occurs in the 3’-end of the probe, which adds the fluorescently-labelled nucleotide. The reviewer is interested on whether SNPs in the 5’-end affect probe hybridization; however, we are unable to answer this question, as the Infinium assay does not detect hybridization per se. Given that the probes employed are rather long (50 bp), it possible that mismatches in the 5’-end do not affect hybridization in the 3’-end. As long as the 3’-end is correctly hybridized, single-base extension can occur independently on whether the 5’-end is fully hybridized. Finally, given that:*

*distance to the 5’-end = probe length – 1 – distance to the 3’-end*

*The resulting plot referencing distance from the 5’ end would simply be the mirror image of the plot shown in Figure 3C. What is clear on this figure is that SNPs closer to the 5’-end (further away from the 3’-end) do not affect single-base extension, though we cannot know whether it affects 5’-end hybridization. We hope that our explanation helps to clarify this topic.*

3. It would be helpful to add additional details to the Figure legend, so that each Figure becomes readable without the readers going back to the main text. Right now, I have to go back to the Results section and even the Methods section in order to fully understand each Figure.

*Reply: We do apologize for the conciseness of the Figure legends. To account for the reviewer’s comment, we have now extended all figure legends in the revised manuscript to include all necessary details, aiming to ease their comprehension.*

**Second round of review**

**Reviewer 1**

no further comments - congratulations to the great work

**Reviewer 2**

All my previous comments were well addressed.
